# CSF tau phosphorylation occupancies at T217 and T205 represent improved biomarkers of amyloid and tau pathology in Alzheimer’s disease

**DOI:** 10.1038/s43587-023-00380-7

**Published:** 2023-03-13

**Authors:** Nicolas R. Barthélemy, Benjamin Saef, Yan Li, Brian A. Gordon, Yingxin He, Kanta Horie, Erik Stomrud, Gemma Salvadó, Shorena Janelidze, Chihiro Sato, Vitaliy Ovod, Rachel L. Henson, Anne M. Fagan, Tammie L. S. Benzinger, Chengjie Xiong, John C. Morris, Oskar Hansson, Randall J. Bateman, Suzanne E. Schindler

**Affiliations:** 1 Department of Neurology, Washington University School of Medicine, St. Louis, MO USA; 2 Tracy Family SILQ Center for Neurodegenerative Biology, St. Louis, MO USA; 3 Department of Radiology, Washington University School of Medicine, St. Louis, MO USA; 4 Clinical Memory Research Unit, Department of Clinical Sciences Malmö, Lund University, Lund, Sweden; 5 Memory Clinic, Skåne University Hospital, Malmö, Sweden; 6 Knight Alzheimer Disease Research Center, Washington University School of Medicine, St. Louis, MO USA; 7 Division of Biostatistics, Washington University School of Medicine, St. Louis, MO USA

**Keywords:** Alzheimer's disease, Mass spectrometry, Ageing

## Abstract

Cerebrospinal fluid (CSF) amyloid-β peptide (Aβ)42/Aβ40 and the concentration of tau phosphorylated at site 181 (p-tau181) are well-established biomarkers of Alzheimer’s disease (AD). The present study used mass spectrometry to measure concentrations of nine phosphorylated and five nonphosphorylated tau species and phosphorylation occupancies (percentage phosphorylated/nonphosphorylated) at ten sites. In the present study we show that, in 750 individuals with a median age of 71.2 years, CSF pT217/T217 predicted the presence of brain amyloid by positron emission tomography (PET) slightly better than Aβ42/Aβ40 (*P* = 0.02). Furthermore, for individuals with positive brain amyloid by PET (*n* = 263), CSF pT217/T217 was more strongly correlated with the amount of amyloid (Spearman’s *ρ* = 0.69) than Aβ42/Aβ40 (*ρ* = −0.42, *P* < 0.0001). In two independent cohorts of participants with symptoms of AD dementia (*n* = 55 and *n* = 90), CSF pT217/T217 and pT205/T205 were better correlated with tau PET measures than CSF p-tau181 concentration. These findings suggest that CSF pT217/T217 and pT205/T205 represent improved CSF biomarkers of amyloid and tau pathology in AD.

## Abstract (web summary)

Cerebrospinal fluid (CSF) tests are used in the clinical diagnosis of Alzheimer’s disease. This study found that CSF tau phosphorylation at two sites (T217 and T205) is a better test for Alzheimer’s disease pathology than currently available tests.

## Main

AD is characterized by the aggregation of Aβ into amyloid plaques and the hyperphosphorylation and accumulation of tau into neurofibrillary tangles, which begins a decade or more before the onset of dementia symptoms. These neuropathological features can be visualized and quantified in living individuals using PET with radiotracers binding to amyloid and tau^1,2^. AD brain pathology is associated with a lower concentration of Aβ42 and a lower ratio of Aβ42:Aβ40 in the CSF, probably due to sequestration of Aβ42 into amyloid plaques^3^, but higher concentrations of CSF total tau (t-tau) and p-tau181 (refs. ^4,5^). Importantly, CSF t-tau and p-tau181 concentrations increase around the time of amyloid plaque deposition, when no neurofibrillary tangles are detected via tau PET, suggesting that elevated CSF t-tau and p-tau181 concentrations may reflect a response to amyloid plaques rather than neurofibrillary tangle burden^6,7^.

Most studies of CSF p-tau have examined only p-tau181, but tau is phosphorylated at many different sites and is truncated in the CSF and plasma^8–11^. Several studies have demonstrated that CSF and/or plasma p-tau217 and p-tau231 concentrations are strongly associated with imaging and clinical measures of AD^12–20^. Immunoassays are widely used to measure concentrations of different p-tau species including p-tau181, p-tau217 and p-tau231, but they employ different antibodies and assay conditions, so in comparative studies it is often unclear whether differences in biomarker associations are related to differences in the analyte or the assay^15,17,19,21,22^. In contrast, mass spectrometry (MS) enables simultaneous measurement of nonphosphorylated and phosphorylated tau species and has high specificity. MS has identified p-tau217, p-tau205 and a species of the microtubule-binding region of tau (MTBR-tau243) as candidate AD biomarkers^23,24^, revealed the degree of phosphorylation at different tau sites in response to AD^23,25^ and enabled comparisons between CSF p-tau species that demonstrate the order in which sites are phosphorylated over the course of AD^26^.

In the present study of older individuals with and without cognitive impairment, MS was used to evaluate 24 different measures of CSF tau: the concentrations of nine phosphorylated and five nonphosphorylated tau peptides, and the phosphorylation occupancy (percentage phosphorylated:nonphosphorylated) at ten sites. This comprehensive evaluation allowed analysis of which CSF tau measures are most strongly associated with amyloid plaques and neurofibrillary tangles as measured by PET, regional brain volumes, clinical status (cognitively unimpaired or impaired) and dementia severity.

## Results

### Characteristics of cohorts

The primary cohorts for the study were from the Knight Alzheimer Disease Research Center (Knight ADRC) at Washington University in St. Louis, MO, USA. The Knight ADRC amyloid PET cohort included 750 individuals with a median age of 71.2 years (interquartile range (IQR) 65.3–76.1 years); 55% were female, 90% self-identified as white, 39% carried at least one apolipoprotein E (*APOE*) ε4 allele and 16% were cognitively impaired as defined by a Clinical Dementia Rating (CDR) of ≥0.5, which includes mild cognitive impairment (MCI) and AD dementia (Extended Data Table 1). The overlapping Knight ADRC tau PET cohort included 371 individuals (Extended Data Table 2). Individuals in the tau PET cohort who were cognitively impaired (CDR ≥ 0.5) were included in Knight ADRC tau PET symptomatic AD subcohort (*n* = 55) (Supplementary Table 1). The validation cohort included 90 individuals enrolled in the BioFINDER-2 cohort at Skåne University Hospital in Sweden with a median age of 72 years (IQR 67–76 years); 47% were female, 71% carried at least one *APOE* ε4 allele, 83 were diagnosed with MCI and 7 were diagnosed with AD dementia (Extended Data Table 3).

### CSF measures and amyloid PET

MS was used to evaluate 24 CSF tau measures and automated Lumipulse assays were used to evaluate 5 measures including Aβ42/Aβ40. The coefficient of variation for each CSF tau measure was estimated by running three pooled CSF samples with each of the 26 batches. For the intermediately abnormal CSF pool, pT231/T231 had higher variance (17.5%) compared with pT217/T217 (3.0%), pT181/T181 (5.8%) and pT205/T205 (9.1%) (see Appendix 1 for methodology, quality control and performance details of the MS assay). Of note, the p-tau species served as the numerator and the corresponding nonphosphorylated tau species served as the denominator for the occupancy measures. Key findings were replicated in subcohorts with no missing CSF biomarker measures.

**Extended Data Fig. 1:**
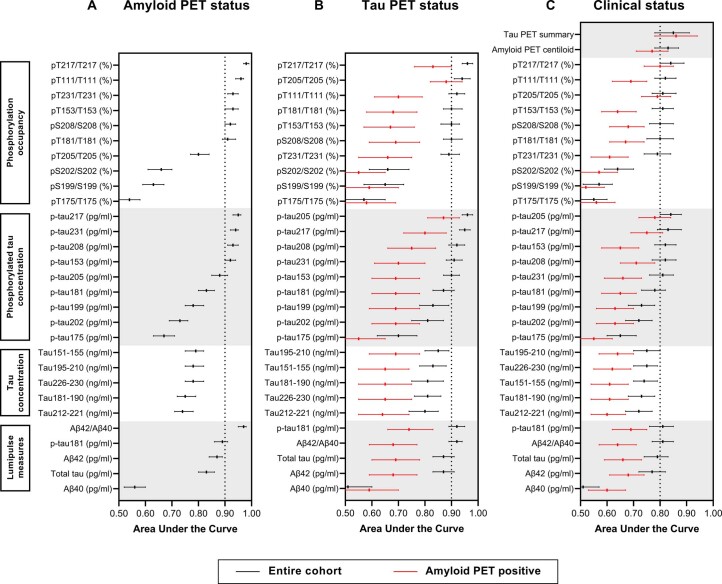
Correspondence of CSF measures with amyloid PET status, tau PET status, or clinical status. Correspondence of the CSF measures with amyloid PET status was evaluated in the amyloid PET cohort (n = 750 individuals) (**A**), and correspondence with tau PET status was evaluated in tau PET cohort (n = 371 individuals) (**B**). Correspondence of the CSF measures with clinical status (cognitively normal [CDR = 0] or cognitively impaired [CDR > 0]) was evaluated in the larger amyloid PET cohort (n = 750 individuals) (**C**). Lines represent the receiver operating characteristic area under the curve point estimate (middle point) and 95% confidence intervals. Black lines represent correspondence in the entire cohort and the red lines represent correspondence in amyloid PET positive individuals.

**Extended Data Fig. 2:**
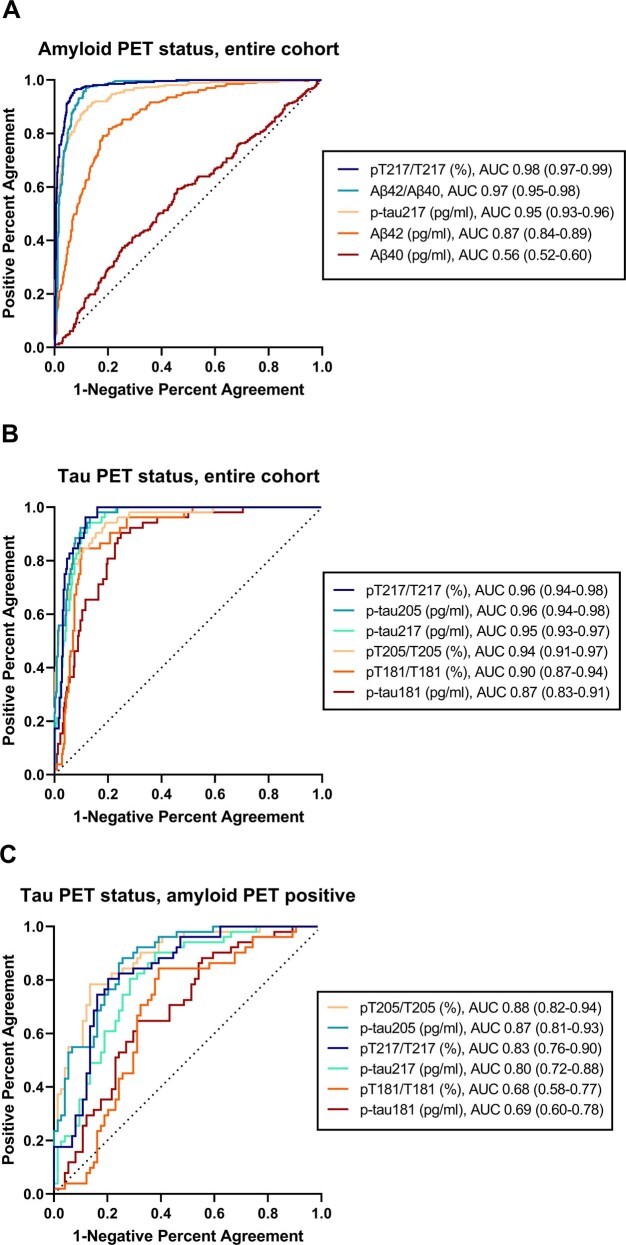
Receiver operating characteristic curves for selected CSF measures. Correspondence of selected CSF measures with amyloid PET status was evaluated in the amyloid PET cohort; n = 750 for all measures shown (**A**). Correspondence of selected CSF measures with tau PET status was evaluated in the tau PET cohort; n = 371 for all measures shown except for pT181/T181 and p-tau181 (n = 370) (**B**). Correspondence of selected CSF measures with tau PET status was evaluated in the amyloid PET positive individuals in the tau PET cohort; n = 125 for all measures shown (**C**). For each measure, the receiver operating characteristic area under the curve with 95% confidence intervals is shown.

The relationships of the CSF measures with amyloid PET status (positive or negative according to established cut-offs) were evaluated in the large (*n* = 750) Knight ADRC amyloid PET cohort (Extended Data Fig. 1a). Concentrations of all phosphorylated and nonphosphorylated CSF tau species were higher in amyloid PET-positive compared with amyloid PET-negative individuals (*P* < 0.0001 for all) and the fold difference was largest for CSF p-tau231 concentration (7.79-fold) (Extended Data Table 4 and Supplementary Table 2). The phosphorylation occupancy at six sites (T217, T111, T231, T153, S208 and T181) and the concentrations of four p-tau species (p-tau217, p-tau231, p-tau208 and p-tau153) distinguished amyloid PET status with a receiver operating characteristic (ROC) area under the curve (AUC) > 0.90 (Supplementary Table 3). CSF pT217/T217 had the highest correspondence with amyloid PET status of all the measures, with an ROC AUC of 0.98 (95% confidence interval (CI) 0.97–0.99), which was slightly superior to Lumipulse Aβ42/Aβ40 (AUC = 0.97 (0.95–0.98), *P* = 0.02) and p-tau217 concentration (AUC = 0.95 (0.93–0.96), *P* < 0.0001; Extended Data Fig. 2a).

**Fig. 1:**
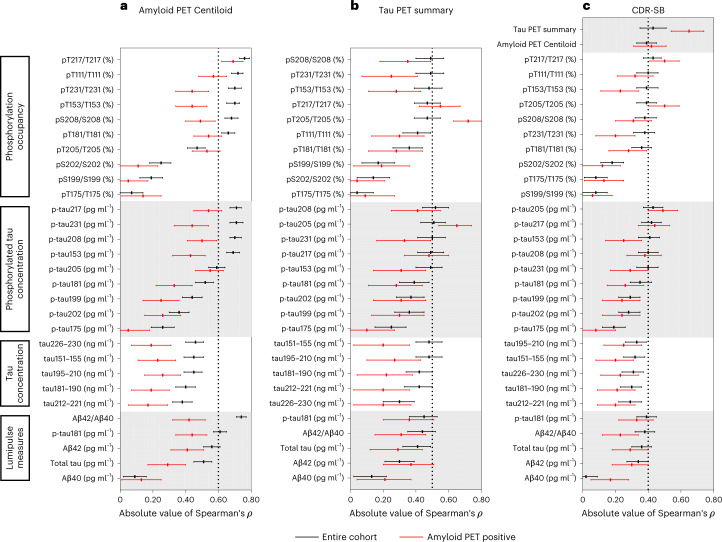
Correlations of CSF measures with amyloid PET Centiloid, the tau PET summary measure or dementia severity. **a**, Correlations of the CSF measures with amyloid PET Centiloid evaluated in the amyloid PET cohort (*n* = 750 individuals). **b**, Correlations with the tau PET summary measure evaluated in the tau PET cohort (*n* = 371 individuals). **c**, Correlations with dementia severity, as measured by the CDR-SB, evaluated in the larger amyloid PET cohort (*n* = 750 individuals). The lines represent Spearman’s correlation (middle point) with 95% CIs. The black lines represent correlations in the entire cohort and the red lines represent correlations in amyloid PET-positive individuals.

**Fig. 2:**
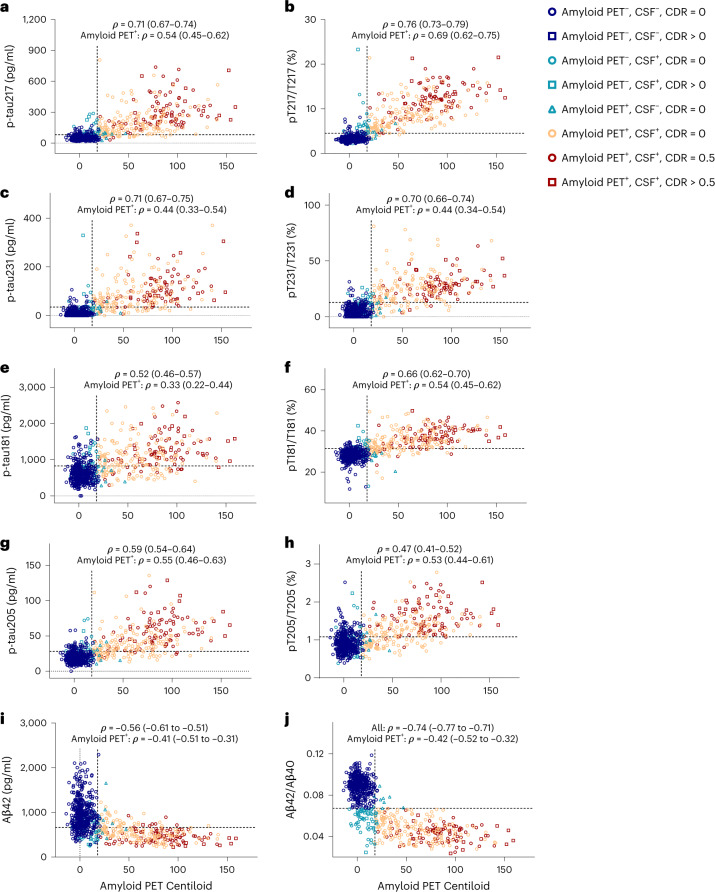
Correlations of selected CSF p-tau concentrations and tau phosphorylation occupancies with amyloid PET Centiloid. **a**–**j**, CSF concentrations of p-tau217 (**a**), p-tau231 (**c**), p-tau181 (**e**), p-tau205 (**g**) and Aβ42 (**i**), and tau phosphorylation occupancies at T217 (**b**), T231 (**d**), T181 (**f**) and T205 (**h**), as well as Aβ42/Aβ40 (**j**), plotted as a function of amyloid PET Centiloid. Spearman’s correlations with 95% CIs are shown for the entire amyloid PET cohort (*n* = 750 individuals) and amyloid PET-positive individuals in the amyloid PET cohort (*n* = 263). The horizontal dashed lines denote the cut-offs that best distinguish amyloid PET status, based on combined sensitivity and specificity (Supplementary Table 3). The vertical dashed lines represent the cut-off for amyloid PET positivity. Each symbol represents one individual: blue circle: amyloid PET negative, CSF negative, CDR = 0; blue square: amyloid PET negative, CSF negative, CDR > 0; green circle: amyloid PET negative, CSF positive, CDR = 0; green square: amyloid PET negative, CSF positive, CDR > 0; green triangle: amyloid PET positive, CSF negative, CDR = 0; orange circle: amyloid PET positive, CSF positive, CDR = 0; red circle: amyloid PET positive, CSF positive, CDR = 0.5; and red square: amyloid PET positive, CSF positive, CDR > 0.5.

The correlations of CSF measures with continuous amyloid PET Centiloid values were examined (Figs. 1a and 2). CSF pT217/T217 had the highest correlation with amyloid PET Centiloid of all the measures (Spearman’s *ρ* = 0.76 (95% CI 0.73–0.79), which was almost significantly higher than Lumipulse Aβ42/Aβ40 (*ρ* = −0.74 (−0.77 to −0.71), *P* = 0.08) and was significantly higher than p-tau217 concentration *(ρ* = 0.71 (0.67–0.74), *P* < 0.0001) (Supplementary Tables 4 and 5). Importantly, in amyloid PET-positive individuals, CSF pT217/T217 was strongly correlated with amyloid PET Centiloid (*ρ* = 0.69 (0.62–0.75)), whereas the correlation between Lumipulse Aβ42/Aβ40 and amyloid PET Centiloid was only modest (*ρ* = −0.42 (−0.52 to −0.32), *P* < 0.0001; Supplementary Table 6).

### CSF measures and tau PET

The overlapping Knight ADRC tau PET cohort was used to evaluate the relationships between CSF measures and tau PET status (Extended Data Fig. 1b). Concentrations of almost all CSF phosphorylated and nonphosphorylated tau species were higher in tau PET-positive compared with PET-negative individuals, with the highest fold difference again being in CSF p-tau231 concentration (7.16-fold) (Supplementary Tables 7 and 8).

The phosphorylation occupancy at six sites (T217, T111, T181, T153, S208 and T231) and the concentrations of four p-tau species (p-tau217, p-tau208, p-tau231and p-tau153) that were strongly associated with amyloid PET status also distinguished tau PET status with an AUC ≥ 0.89 (Supplementary Table 9). It is interesting that two measures that were not strongly associated with amyloid PET status, pT205/T205 and p-tau205 concentration, were among the best predictors of tau PET status (AUC = 0.94 and 0.96, respectively). The CSF measures with the highest correspondence with tau PET status included pT217/T217 (AUC = 0.96 (0.94–0.98), p-tau205 concentration (AUC = 0.96 (0.94–0.98)), p-tau217 concentration (AUC = 0.95 (0.93–0.97)) and T205/T205 (AUC = 0.94 (0.91–0.97) (Extended Data Fig. 2b).

In amyloid PET-positive individuals, only CSF measures related to T205 and T217 distinguished tau PET status with an AUC > 0.75: pT205/T205 (AUC = 0.88 (0.82–0.94), p-tau205 concentration (AUC = 0.87 (0.81–0.93)), pT217/T217 (AUC = 0.83 (0.76–0.90)) and p-tau217 concentration (AUC = 0.80 (0.72–0.88)) (Extended Data Fig. 2c and Supplementary Table 10). Individuals who were both amyloid PET positive and tau PET positive could be distinguished from all other individuals by single measures, including CSF pT217/T217 (AUC = 0.96 (0.94–0.98)) (Supplementary Table 11).

**Fig. 3:**
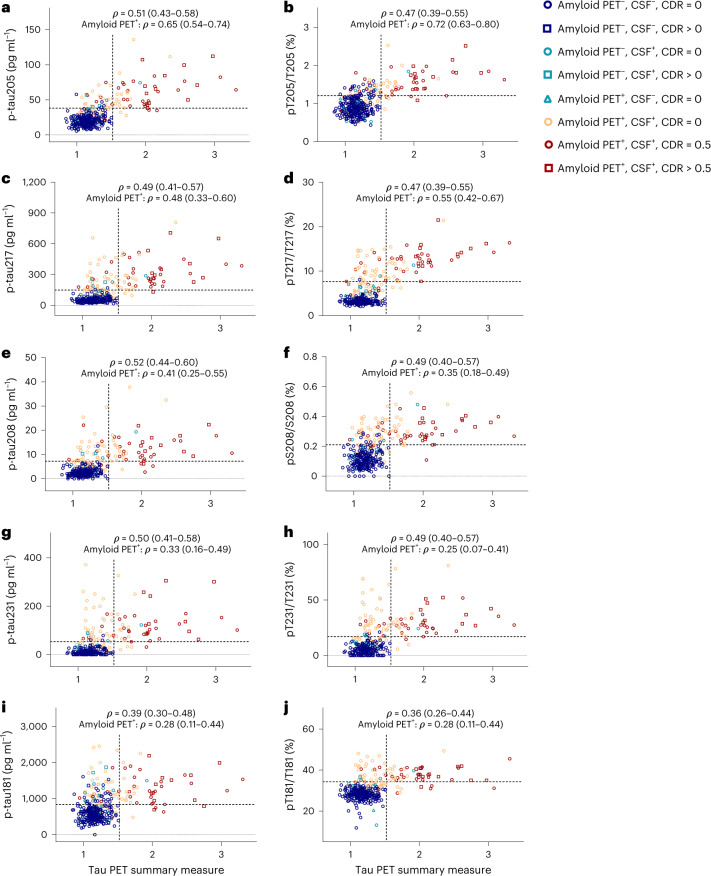
Correlations of selected CSF p-tau concentrations and tau phosphorylation occupancies with the tau PET summary measure. **a**–**j**, CSF concentrations of p-tau205 (**a**), p-tau217 (**c**), p-tau208 (**e**), p-tau231 (**g**) and p-tau181 (**i**), and tau phosphorylation occupancies at T205 (**b**), T217 (**d**), S208 (**f**), T231 (**h**), and T181 (**j**), plotted as a function of the tau PET summary measure. Spearman’s correlations with 95% CIs are shown for the entire tau PET cohort (*n* = 371) and amyloid PET-positive individuals in the tau PET cohort (*n* = 125). The horizontal dashed lines denote the cut-offs that best distinguish tau PET status, based on combined sensitivity and specificity (Supplementary Table 9). The vertical dashed lines represent the cut-off for tau PET positivity. Each symbol represents one individual: blue circle: amyloid PET negative, CSF negative, CDR = 0; blue square: amyloid PET negative, CSF negative, CDR > 0; green circle: amyloid PET negative, CSF positive, CDR = 0; green square: amyloid PET negative, CSF positive, CDR > 0; green triangle: amyloid PET positive, CSF negative, CDR = 0; orange circle: amyloid PET positive, CSF positive, CDR = 0; red circle: amyloid PET positive, CSF positive, CDR = 0.5; and red square: amyloid PET positive, CSF positive, CDR > 0.5.

Correlations of CSF measures with a continuous tau PET summary measure were evaluated (Figs. 1b and 3). In the Knight ADRC tau PET cohort, CSF concentrations of p-tau208 (*ρ* = 0.52 (0.44–0.60)), p-tau205 (*ρ* = 0.51 (0.43–0.58), pT217/T217 (*ρ* = 0.47 (0.39–0.55)) and pT205/T205 (*ρ* = 0.47 (0.39–0.55)) had among the highest correlations with the tau PET summary measure (Supplementary Tables 12 and 13). In amyloid PET-positive individuals (*n* = 125), CSF pT205/T205 had the highest correlation with the tau PET summary measure (*ρ* = 0.72 (0.63–0.80)), which was not superior statistically to p-tau205 concentration (*ρ* = 0.65 (0.54–0.74)), but was superior to pT217/T217 (*ρ* = 0.55 (0.42–0.67), *P* = 0.02) and p-tau217 concentration (*ρ* = 0.48 (0.33–0.60), *P* = 0.0002) (Supplementary Table 14).

### CSF measures and regional tau PET and brain volumes

**Fig. 4:**
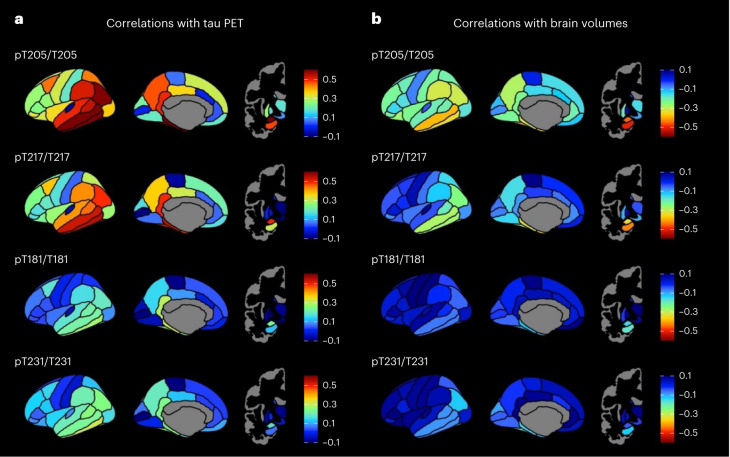
Correlations of selected CSF tau phosphorylation occupancies with regional tau PET and regional brain volumes for amyloid PET-positive individuals. **a**, Partial Spearman’s correlations of selected CSF measures with regional tau PET adjusted for age and sex shown for amyloid PET-positive individuals in the tau PET cohort (*n* = 125). **b**, Partial Spearman’s correlations of CSF measures with regional brain volumes adjusted for age and sex shown for amyloid PET-positive individuals in the larger amyloid PET cohort (*n* = 263).

The correlation of select CSF measures with regional tau PET in amyloid PET-positive individuals in the Knight ADRC tau PET cohort is shown in Fig. 4a. CSF pT205/T205 had the highest correlation with tau PET standardized uptake value ratio (SUVR) in the amygdala and temporal regions (for example, inferior temporal gyrus, fusiform gyrus, parahippocampal gyrus, middle temporal gyrus and entorhinal), with partial Spearman’s *ρ* values ranging from 0.68 to 0.61 for these regions (Supplementary Table 15). CSF pT217/T217 was also most highly correlated with regional tau PET in the amygdala and temporal regions, but the correlations were weaker, with partial Spearman’s *ρ* values ranging from 0.55 to 0.49 for the top six most correlated regions of interest (ROIs) (Supplementary Table 16). The pattern of regional correlations with tau PET were similar for CSF pT181/T181 and pT231/T231, but the maximum partial Spearman’s *ρ* value was 0.31 for both measures (Supplementary Tables 17 and 18).

The correlation of select CSF measures with regional brain volumes in amyloid PET-positive individuals in the Knight ADRC amyloid PET cohort is shown in Fig. 4b and is similar to the patterns seen with tau PET, but the correlations were more modest. CSF pT205/T205 had the highest correlation with regional brain volumes in the hippocampus, amygdala and temporal regions (for example, temporal pole, middle temporal gyrus, fusiform gyrus and entorhinal), with partial Spearman’s *ρ* values ranging from −0.50 to −0.37 for these regions (Supplementary Table 19). CSF pT217/T217 had the highest correlation with regional brain volumes in similar areas, with partial Spearman’s *ρ* values ranging from −0.43 to −0.28 for the top six most correlated ROIs (Supplementary Table 20). After correction for multiple comparisons, CSF pT181/T181 and pT231/T231 were not significantly correlated with regional brain volumes (Supplementary Tables 21 and 22).

### Biomarker measures and dementia

The relationship of CSF measures, amyloid PET and tau PET with clinical status were evaluated in the large Knight ADRC amyloid PET cohort. Clinical status (cognitively unimpaired (CDR = 0) or cognitively impaired (CDR > 0, includes both MCI and AD dementia)) was predicted by biomarkers with and without the covariates of age, sex and years of education (Extended Data Fig. 1c and Supplementary Table 23). The tau PET summary measure best distinguished cognitively impaired from cognitively unimpaired individuals (AUC = 0.85 (0.78–0.91)), but this was not superior statistically to several CSF measures including pT217/T217 (AUC = 0.84 (0.80–0.89), p-tau205 concentration (AUC = 0.84 (0.80–0.88)), p-tau217 concentration (AUC = 0.83 (0.79–0.88)) or pT205/T205 (AUC = 0.81 (0.77–0.86)). In amyloid PET-positive individuals, the tau PET summary measure also had the highest correspondence with clinical status (AUC = 0.86 (0.78–0.94)), but again this was not statistically superior to pT217/T217 (AUC = 0.80 (0.74–0.85)) or pT205/T205 (AUC = 0.79 (0.73–0.84)) (Supplementary Table 24). Notably, CSF Lumipulse measures (for example, Aβ42/Aβ40, p-tau181 concentration) had relatively low performance in predicting clinical status (AUC = 0.60–0.69) in amyloid PET-positive individuals.

Correlations of amyloid PET, tau PET and CSF biomarkers with dementia severity as measured by the CDR sum of boxes (CDR-SB) were also examined in the large Knight ADRC amyloid PET cohort (Fig. 1c). The tau PET summary measure (*ρ* = 0.43 (0.35–0.51), CSF pT217/T217 (*ρ* = 0.43 (0.37–0.48)) and pT205/T205 (*ρ* = 0.39 (0.33–0.45)) had some of the highest correlations with CDR-SB (Supplementary Table 25). In amyloid PET-positive individuals, the CDR-SB had the highest correlations with the tau PET summary measure (*ρ* = 0.65 (0.54–0.74)), CSF pT217/T217 (*ρ* = 0.50 (0.41–0.59)) and pT205/T205 (*ρ* = 0.50 (0.40–0.59)), whereas correlations with Lumipulse Aβ42/Aβ40 were lower (*ρ* = −0.23 (−0.34 to −0.12); Supplementary Table 26).

### Biomarker measures in a symptomatic AD cohort

To evaluate the performance of these measures in a more clinically relevant cohort, CSF tau measures were examined in cognitively impaired (CDR ≥ 0.5) individuals from the Knight ADRC tau PET cohort (*n* = 55; Supplementary Table 27). There was a trend toward a higher correlation of amyloid PET Centiloid with CSF pT217/T217 (*ρ* = 0.77 (0.63–0.86)) compared with Aβ42/Aβ40 (*ρ* = −0.58 (−0.73 to −0.37), *P* = 0.06; Supplementary Table 28). CSF pT217/T217 (*ρ* = 0.71 (0.55–0.82) and/or pT205/T205 (*ρ* = 0.67 (0.50–0.80)) had higher correlations with a tau PET summary measure compared with pT181/T181 (*ρ* = 0.42 (0.18–0.62), *P* = 0.006 and *P* = 0.04) or Lumipulse p-tau181 concentration (*ρ* = 0.44 (0.20–0.63), *P* = 0.003 and *P* = 0.07; Supplementary Table 29).

### Biomarker measures in a confirmatory symptomatic AD cohort

**Extended Data Fig. 3:**
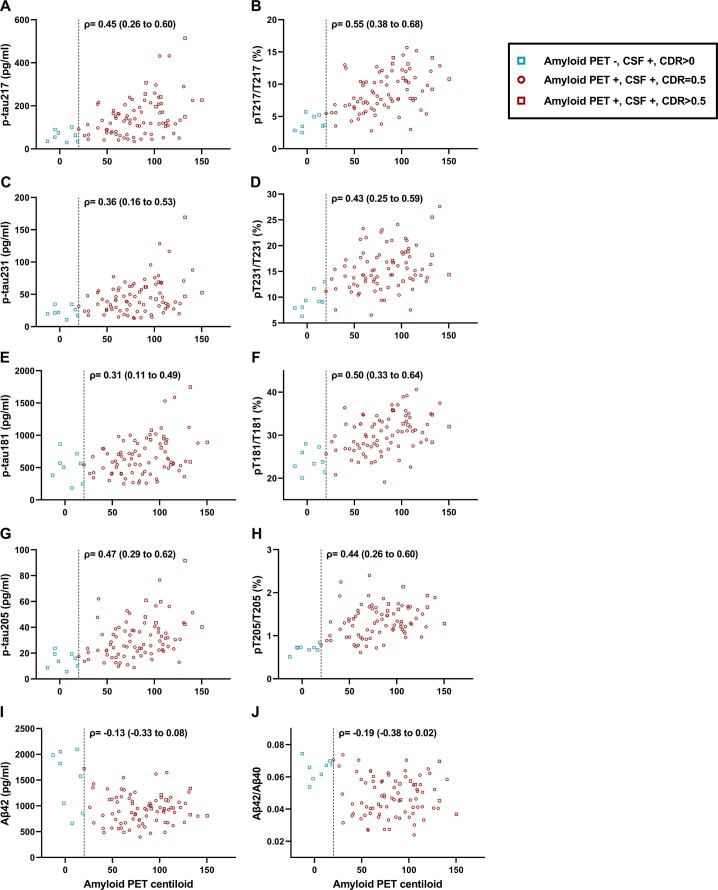
Correlations of selected CSF p-tau concentrations and tau phosphorylation occupancies with amyloid PET Centiloid in the BioFINDER-2 cohort. CSF concentrations of p-tau217 (**A**), p-tau231 (**C**), p-tau181 by MS (**E**), p-tau205 (**G**), and Aβ42 (**I**) and tau phosphorylation occupancies at T217 (**B**), T231 (**D**), T181 (**F**), T205 (**H**), as well as Aβ42/Aβ40 (**J**), are plotted as a function of amyloid PET Centiloid. Spearman correlations with 95% confidence intervals are shown. Vertical dashed lines represent the cut-off for amyloid PET positivity. Each symbol represents one individual: green square: amyloid PET negative, CSF positive, CDR > 0; red circle: amyloid PET positive, CSF positive, CDR = 0.5; red square: amyloid PET positive, CSF positive, CDR > 0.5.

**Extended Data Fig. 4:**
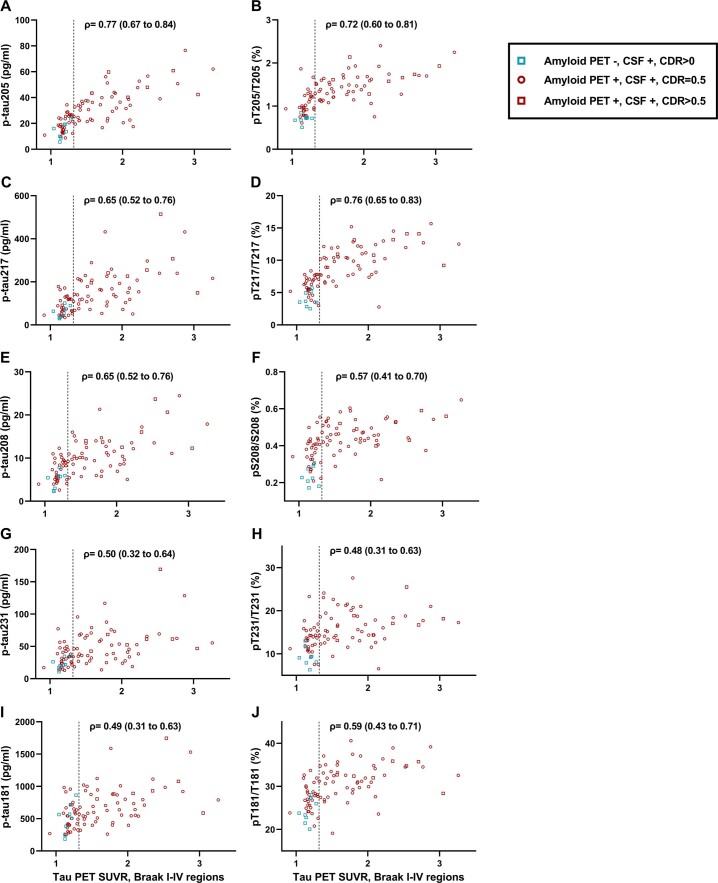
Correlations of selected CSF p-tau concentrations and tau phosphorylation occupancies with tau PET SUVR for Braak I-IV regions in the BioFINDER-2 cohort. CSF concentrations of p-tau205 (**A**), p-tau217 (**C**), p-tau208 (**E**), p-tau231 (**G**), and p-tau181 by MS (**I**) and tau phosphorylation occupancies at T205 (**B**), T217 (**D**), S208 (**F**), T231 (**H**), as well as T181 (**J**), are plotted as a function of the tau PET SUVR for Braak I-IV regions. Spearman correlations with 95% confidence intervals are shown. Vertical dashed lines represent the cut-off for tau PET positivity (SUVR > 1.32 for Braak I-IV regions). Each symbol represents one individual: green square: amyloid PET negative, CSF positive, CDR > 0; red circle: amyloid PET positive, CSF positive, CDR = 0.5; red square: amyloid PET positive, CSF positive, CDR > 0.5.

**Extended Data Fig. 5:**
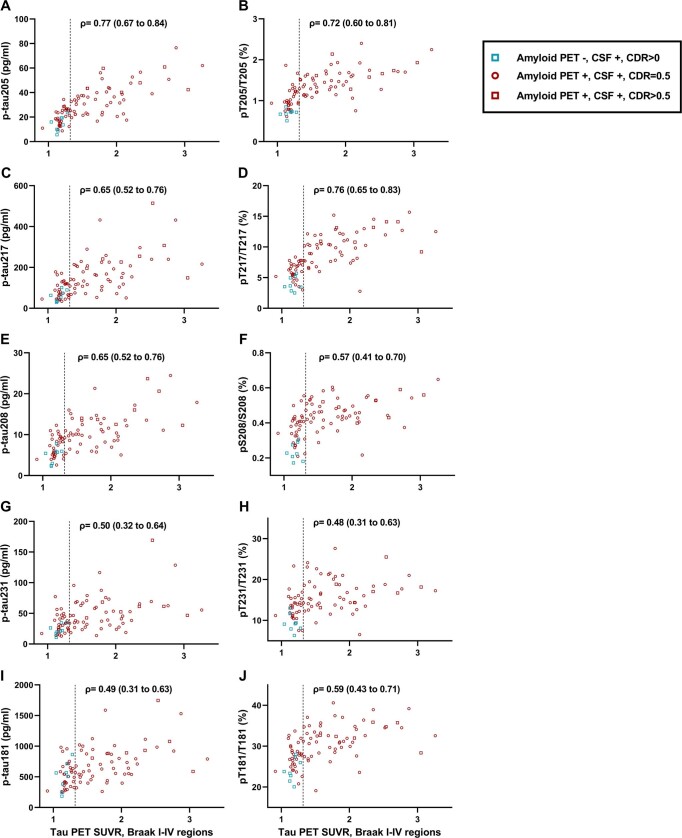
Correlations of selected CSF p-tau concentrations and tau phosphorylation occupancies with tau PET SUVR for Braak V-VI regions in the BioFINDER-2 cohort. CSF concentrations of p-tau205 (**A**), p-tau217 (**C**), p-tau208 (**E**), p-tau231 (**G**), and p-tau181 by MS (**I**) and tau phosphorylation occupancies at T205 (**B**), T217 (**D**), S208 (**F**), T231 (**H**), as well as T181 (**J**), are plotted as a function of the tau PET SUVR for Braak V-VI regions. Spearman correlations with 95% confidence intervals are shown. Each symbol represents one individual: green square: amyloid PET negative, CSF positive, CDR > 0; red circle: amyloid PET positive, CSF positive, CDR = 0.5; red square: amyloid PET positive, CSF positive, CDR > 0.5.

The CSF tau measures were further examined in participants with symptomatic AD (MCI or AD dementia) from the BioFINDER-2 cohort (*n* = 90; Extended Data Table 5). CSF pT217/T217 (*ρ* = 0.55 (0.38–0.68)) was better correlated with amyloid PET Centiloid than CSF Aβ42/Aβ40 (*ρ* = −0.19 (−0.38 to 0.02), *P* = 0.0007; Extended Data Fig. 3 and Supplementary Table 30). CSF pT217/T217 (*ρ* = 0.76 (0.65–0.83)) and/or pT205/T205 (*ρ* = 0.72 (0.60–0.81)) had higher correlations with a tau PET measure for the Braak I–IV region compared with pT181/T181 (*ρ* = 0.59 (0.43–0.71), *P* = 0.002 and *P* = 0.14) and CSF p-tau181 concentration by immunoassay (*ρ* = 0.42 (0.23–0.57), *P* = 0.0004 and *P* = 0.02, respectively; Extended Data Fig. 4 and Supplementary Table 31). CSF pT217/T217 (*ρ* = 0.59 (0.43–0.71)) and pT205/T205 (*ρ* = 0.66 (0.52–0.76)) were better correlated with a tau PET measure for the Braak V–VI region compared with pT181/T181 (*ρ* = 0.44 (0.25–0.59), *P* = 0.004 and 0.02) and p-tau181 concentration by immunoassay (*ρ* = 0.23 (0.02–0.42), *P* = 0.0003 and *P* = 0.0007; Extended Data Fig. 5 and Supplementary Table 32).

## Discussion

In the present study, we examined the relationships of 24 CSF tau MS measures and 5 CSF Lumipulse automated immunoassay measures with amyloid PET, tau PET, regional brain volumes, clinical status and dementia severity. The CSF measure that best distinguished amyloid PET status was not Aβ42, Aβ42/Aβ40 or even p-tau217 concentration, but pT217/T217. Moreover, CSF pT217/T217 was strongly correlated with amyloid PET Centiloid in amyloid PET-positive individuals in whom the correlation between Aβ42/Aβ40 and amyloid PET Centiloid was only modest. Overall, although six of the ten sites investigated (T217, T111, T231, T153, S208 and T181) were strongly associated with amyloid PET status, only CSF pT217/T217 was also strongly associated with tau PET status. In contrast, CSF pT205/T205 was not strongly associated with amyloid status, but, in amyloid PET-positive individuals, pT205/T205 had the highest correlations with the tau PET summary measure, regional tau PET measures and regional brain volumes. In amyloid PET-positive individuals, the tau PET summary measure best distinguished cognitively impaired from cognitively unimpaired individuals, but it was not statistically superior to CSF pT217/T217 or pT205/T205. Overall, these data demonstrate that CSF pT217/T217 is a superior biomarker of amyloid plaque burden and pT205/T205 is a promising biomarker of neurofibrillary tangle burden in individuals with brain amyloidosis.

The amyloid–tau–neurodegeneration (ATN) framework has posited that CSF Aβ42 or Aβ42/Aβ40 is a measure of amyloid plaques (A) and that CSF p-tau (especially p-tau181) is a measure of neurofibrillary tangles (T)^27^. However, our study found that CSF pT217/T217 was superior to Aβ42/Aβ40 in reflecting amyloid plaque burden and was also strongly associated with tau PET status, demonstrating that a single measure may reflect the presence of both amyloid and tau (A and T). Notably, CSF pT217/T217 was not well correlated with tau PET in amyloid PET-positive individuals, suggesting that the association of pT217/T217 with tau PET status could be partially driven by the high amyloid levels in individuals who are tau PET positive. Concentrations and/or phosphorylation occupancies associated with sites T111, T231, T153, S208 and T181 were also highly associated with amyloid PET, but unlike T217 they were not strongly associated with tau PET, suggesting that these biomarkers may more specifically reflect amyloid (A). Our findings agree with the conclusions of other studies that p-tau217, p-tau181 and p-tau231 change early in AD in response to amyloid^16,28–31^. In contrast, CSF pT205/T205 was only modestly associated with amyloid PET, but was strongly associated with tau PET in amyloid PET-positive individuals, indicating that pT205/T205 is a relatively specific biomarker of tau (T). CSF pT205/T205 had the highest correlations with brain volumes, suggesting that pT205/T205 may also be a biomarker of neurodegeneration (N). The relationships between CSF tau measures and other biomarkers probably reflect the timing of biomarker changes in AD: previous work suggests that T217 is phosphorylated early in AD around the time of amyloid plaque deposition, whereas T205 is phosphorylated later and closer to neurofibrillary tangle accumulation and symptom onset^26^. The present study demonstrates the complexity of the relationship between tau phosphorylation and AD pathology, which may defy simple categorization.

An advantage of MS over immunoassays is that different protein species can be specifically and simultaneously identified^32^. MS enables assessment of measures such as phosphorylation occupancy, which may be difficult to evaluate via immunoassay. In the present study, CSF pT217/T217 had slightly but significantly higher performance compared with p-tau217 in prediction of amyloid PET status, which could be meaningful when accuracy is highly valued (for example, clinical diagnosis). It is possible that phosphorylation occupancies attenuate interindividual variability unrelated to AD pathology which affects the concentrations of both phosphorylated and nonphosphorylated tau species^33,34^. Notably, the CSF biomarker assays that are currently available for clinical AD diagnosis do not include p-tau217, p-tau205, pT217/T217 or pT205/T205. Although immunoassays are more widely available than specialized MS assays, MS may potentially provide unique and better performing measures than immunoassays.

The study cohorts consisted of participants enrolled in research studies and, therefore, the findings are most relevant to research studies and clinical trials and less generalizable to clinical populations. The cohorts were relatively young (early to mid-70s). Older cohorts, especially clinical cohorts, would be expected to have a higher prevalence of neurological comorbidities, which could complicate biomarker relationships. The cohorts included relatively few individuals with symptomatic AD and/or moderate or high levels of tau pathology. In addition, minoritized populations were not well represented in the study cohorts and studies have found racial differences in CSF t-tau and p-tau181 concentrations^35,36^. It is unknown whether other CSF tau measures such as pT217/T217 or pT205/T205 perform similarly across racial and ethnic groups, and further studies of these measures in diverse and clinically relevant cohorts are needed.

Technical limitations include that tau was immunopurified for analysis, which may result in some species of tau not being recovered well. Data for some CSF tau measures (particularly those related to p-tau231) did not meet quality control criteria for a subset of the samples and were thus excluded from analyses. The variance for p-tau231 measures was higher than for p-tau217 measures, which could have resulted in underestimating the performance of p-tau231 measures. Measures associated with p-tau231 had the largest fold-change between amyloid PET-negative and amyloid PET-positive individuals, and between tau PET-negative and tau PET-positive individuals, but the higher variance led to reduced associations of p-tau231 measures with outcomes. A limitation of the comparisons between CSF Aβ42/Aβ40 and tau measures is that CSF Aβ peptides were measured with immunoassays rather than MS, and it is possible that MS measurements of CSF Aβ42/Aβ40 could perform better. Overall, it is important to consider that the observed relationship between a biomarker and AD depends not only on the biological relationship, but also on the technical characteristics of the assay.

Future directions for this work include evaluating longitudinal trajectories of CSF tau measures to define how these measures change over the time course of sporadic AD, and whether they predict progression from cognitive normality to symptomatic AD. It will also be important to evaluate these CSF tau measures in non-AD dementias, including primary tauopathies^37,38^. Comprehensive evaluation of corresponding tau species in blood may yield improved AD biomarkers that are more accessible to researchers and patients. In particular, plasma pT217/T217 is highly correlated with CSF pT217/T217 (ref. ^14^) and a recent head-to-head study of ten plasma tau assays (including key p-tau181, p-tau217 and p-tau231 assays) found that plasma pT217/T217, as measured by MS, had the best performance in predicting amyloid PET status and progression to AD dementia^17^. Studies of the correspondence of plasma pT205/T205, tau PET and clinical AD symptoms are needed. Overall, the present study demonstrates that different CSF tau species represent different aspects of AD, and that pT217/T217 and pT205/T205 may be superior to the CSF biomarkers of AD currently in widespread use.

## Methods

### Participants

Participants in the Knight Alzheimer Disease Research Center (ADRC) at Washington University in St. Louis were community-dwelling volunteers enrolled in studies of memory and aging. Most participants were recruited from memory clinics or self-referred due to interest in dementia. Individuals were excluded from enrollment if they were diagnosed with a non-AD dementia at their initial assessment (for example, Parkinson’s disease), had conditions that might interfere with study procedures (for example, a pacemaker that would make the participant ineligible for magnetic resonance imaging (MRI)) or had medical issues that might affect long-term participation (for example, metastatic cancer). All procedures were approved by the Washington University Human Research Protection Office (HRPO) and written informed consent was obtained from each participant or their legally authorized representative when appropriate (protocol no. 201109100). Participants received compensation for time spent undergoing procedures, as approved by the HRPO. All participants underwent a comprehensive clinical assessment that included a detailed interview of a collateral source, a neurological examination of the participant, the CDR^39^, the CDR-SB and the Mini-Mental State Examination. Individuals with a CDR ≥ 0.5 were considered to have a dementia syndrome, and the probable etiology of the dementia syndrome was formulated by clinicians based on clinical features in accordance with standard criteria and methods^40^. *APOE* genotype was determined as previously described^41^. Sex and race were self-identified. Participants included in the present study underwent a clinical assessment and lumbar puncture (LP) within 2 years of an amyloid PET scan (amyloid PET cohort). An overlapping cohort was selected in which participants underwent both an amyloid PET and a tau PET scan within 2 years of an LP (tau PET cohort). Previous work has demonstrated that the relationship between CSF biomarkers and amyloid PET is stable if CSF is collected up to 6 years before or 2 years after amyloid PET^42^. If more than one CSF sample from a participant met the criteria, the most recently obtained sample was included. No data points were excluded from analyses and outliers were not removed.

Participants in the Swedish BioFINDER-2 confirmatory cohort (NCT03174938) included individuals diagnosed with MCI due to AD or AD dementia. Details on recruitment, exclusion and inclusion criteria have been described previously^12^. All participants included underwent an LP and cognitive testing within 2 years of both an amyloid PET scan and a tau PET scan. All participants gave written informed consent and ethics approval was granted by the Regional Ethical Committee in Lund, Sweden.

### CSF collection and immunoassays

For studies in the Knight ADRC cohorts, 20–30 ml of CSF was collected via LP at approximately 8am after overnight fasting. CSF was collected in a 50-ml poly(propylene) tube via a gravity drip using an atraumatic Sprotte 22-gauge spinal needle. The entire sample was gently inverted to disrupt potential gradient effects and centrifuged at low speed to pellet any cellular debris. CSF was then aliquoted into poly(propylene) tubes and stored at −80 °C. Aβ42, Aβ40, t-tau and p-tau181 concentrations were measured with a single lot of reagents for all samples with an automated immunoassay platform (LUMIPULSE G1200, Fujirebio) according to the manufacturer’s specifications.

For the BioFINDER-2 confirmatory cohort, CSF was collected as previously described^12^. Aβ42, Aβ40, t-tau and p-tau181 concentrations were measured using fully automated Elecsys immunoassays^43^.

### Measurement of CSF tau peptides

All samples from an individual were run in the same batch. CSF tau was immunopurified and digested, then phosphorylated and nonphosphorylated peptides were quantified using high-resolution MS (HRMS) as previously described and detailed in Appendix 1 (ref. ^25^). Briefly, tau was immunopurified by incubating 450 µl of CSF with tau1 and HJ8.5 antibodies covalently attached to Sepharose beads at room temperature for 4 h. Immunopurified tau was digested for 16 h at 37 °C with 400 ng of trypsin (Promega). AQUA peptides (Life Technologies) were added to a final sample concentration of 5 fmol per labeled phosphorylated peptide and 50 fmol per labeled unmodified peptide. Samples were subjected to liquid chromatography–tandem HRMS (LC-MS/HRMS) analysis on a nanoAcquity ultra-performance LC system (Waters) coupled to an Orbitrap Tribrid Eclipse mass spectrometer (Thermo Fisher Scientific) operating in parallel reaction monitoring mode. MS/HRMS transitions were extracted using Skyline v.22.2.2.278 (MacCoss lab, University of Washington). Data were aggregated using Tableau v.2022.2.2 (Tableau Software) to calculate concentrations and phosphorylation occupancies. All assays and data extraction steps were performed by operators blinded to any clinical or biomarker information.

### Amyloid and tau PET imaging

Participants at the Knight ADRC underwent amyloid PET using either [^18^F]AV45 (florbetapir) or [^11^C]Pittsburgh Compound B (PiB). An overlapping cohort additionally underwent tau PET with [^18^F]AV1451 (flortaucipir). Both amyloid PET and tau PET scans were performed in coordination with a 3-T structural MRI scan. T1-weighted MRIs were processed using Freesurfer 5.3 to generate ROIs used for the processing of PET data. Estimates of regional volumes were adjusted for intracranial volume using a regression approach. Data from the 30- to 60-min post-injection window for PiB, the 50- to 70-min window for florbetapir or the 80- to 100-min window for flortaucipir were converted to SUVRs using the cerebellar gray as a reference and partial volume corrected using a geometric transfer matrix^44^. Values from the following regions were averaged together to represent mean cortical SUVR for florbetapir or PiB: bilateral orbitofrontal, medial orbitofrontal, rostral middle frontal, superior frontal, superior temporal, middle temporal and precuneus. Amyloid PET positivity was previously defined as a mean cortical SUVR > 1.42 for PiB and >1.19 for florbetapir^45^. Mean cortical SUVRs were converted to Centiloid units to combine data from the two tracers^45,46^. Values from the bilateral entorhinal cortex, amygdala, lateral occipital cortex and inferior temporal cortex regions were averaged together as a summary measure of tau PET^47^. Tau PET positivity was defined as a tau PET summary measure >1.52, based on Gaussian mixture modeling (Supplementary Fig. 1).

Participants in BioFINDER-2 underwent amyloid PET using [^18^F]flutemetamol as previously described^12^. Participants underwent tau PET using [^18^F]RO948 as previously described^12^. For tau PET, SUVRs for the brain regions with early change (Braak I–IV region) and later change (Braak V–VI region) were calculated. The cut-off for positivity in the Braak I–IV region was SUVR > 1.32 (ref. ^48^).

### Statistics and reproducibility

No statistical methods were used to predetermine sample sizes but our sample sizes are similar to or larger than those used for similar studies^16,26,31^. Measured values for many biomarkers were not normally distributed and therefore nonparametric analyses were performed. The significance of differences by biomarker status (amyloid PET or tau PET status) were evaluated with Wilcoxon’s rank-sum tests for continuous variables and χ^2^ or Fisher’s exact test for categorical variables. ROC analyses were used to evaluate the correspondence of CSF biomarker measures with amyloid PET status, tau PET status or clinical status (cognitively unimpaired (CDR = 0) or cognitively impaired (CDR > 0)). Cut-offs that best distinguished amyloid PET or tau PET status were found based on the highest combined sensitivity and specificity (Youden’s index). Differences between ROC AUCs were evaluated using DeLong tests^49^. Spearman’s correlations were used to evaluate the continuous relationships of CSF biomarker measures with amyloid PET Centiloid, the tau PET summary measure or the CDR-SB. For partial Spearman’s correlations with amyloid PET, tau PET and brain volumes, analyses included covariates of age and sex; for correlations with CDR-SB, analyses included covariates of age, sex and years of education. Comparisons between Spearman’s correlations were performed by bootstrapping. When multiple measures were compared, the significance was adjusted using the Benjamini–Hochberg procedure^50^. Analyses were replicated in the subcohort of individuals with no missing data. Statistical analyses were implemented using SAS 9.4. Plots were created with GraphPad Prism v.9.2.0. All *P* values were from two-sided tests and results were deemed statistically significant at *P* < 0.05.

For visualization of the associations between CSF measures and regional tau PET or brain volumes, partial Spearman’s correlations, including age and sex, were calculated with partial.r from the R psych toolbox v.2.1.9. The ggseg package v.1.6.5 was used to visualize correlations and results from the left hemisphere are shown. The significance of correlations was adjusted for multiple comparisons using the Benjamini–Hochberg procedure^50^.

### Reporting summary

Further information on research design is available in the Nature Portfolio Reporting Summary linked to this article.

### Supplementary information

Supplementary Information Supplementary Fig. 1, Tables 1–32 and Appendix 1.

Reporting Summary

## Figures and Tables

**Extended Data Table 1:**
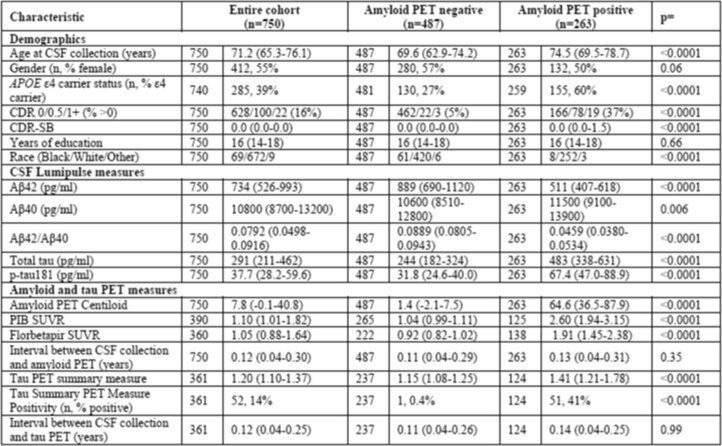
Participant characteristics for the Knight ADRC amyloid PET cohort

Continuous values are presented as the median with the IQR. The significance of differences by amyloid PET status were evaluated with Wilcoxon’s rank-sum tests for continuous variables and χ^2^ or Fisher’s exact test for categorical variables. All tests were two sided and not adjusted for multiple comparisons.

**Extended Data Table 2:**
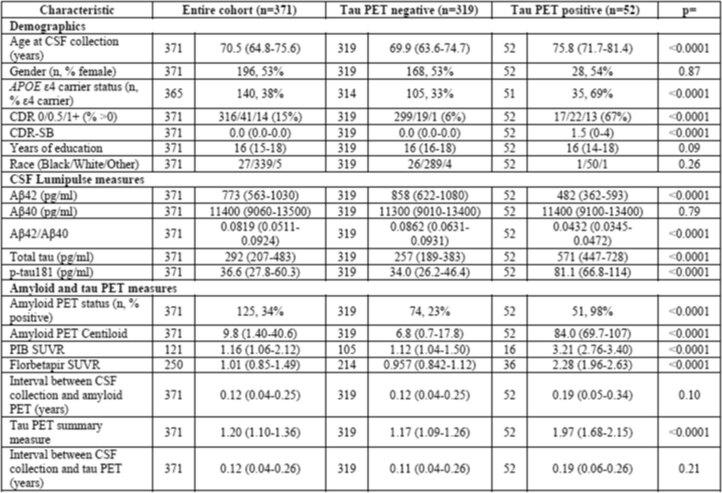
Participant characteristics for the Knight ADRC tau PET cohort

Continuous values are presented as the median with the IQR. The significance of differences by tau PET status were evaluated with Wilcoxon’s rank-sum tests for continuous variables and χ^2^ and Fisher’s exact test for categorical variables. All tests were two sided and not adjusted for multiple comparisons.

**Extended Data Table 3:**
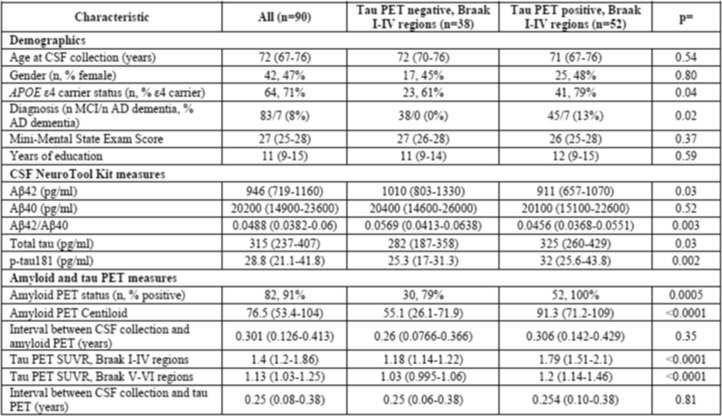
Participant characteristics for the BioFINDER-2 cohort

Continuous values are presented as the median with the IQR. The significance of differences by tau PET status were evaluated with Wilcoxon’s ranked sum tests for continuous variables and χ^2^ or Fisher’s exact test for categorical variables. The cut-off for tau PET positivity was set at SUVR > 1.32 for the Braak I–IV region. All tests were two sided and not adjusted for multiple comparisons.

**Extended Data Table 4:**
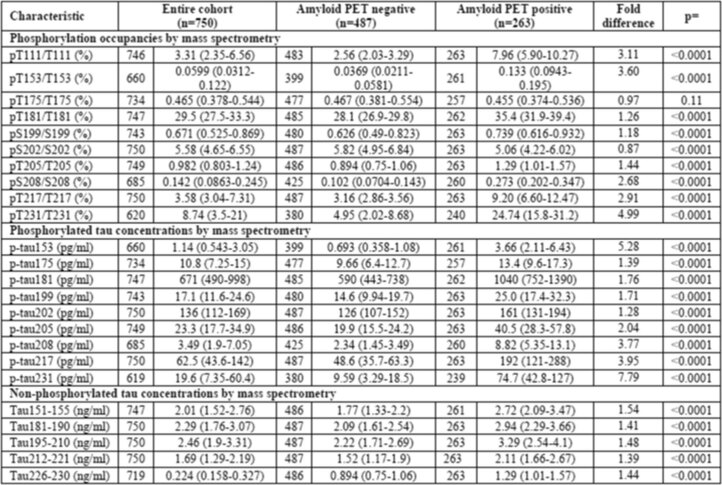
CSF tau measures for the Knight ADRC amyloid PET cohort

Continuous values are presented as the median with the IQR. The significance of differences by amyloid PET status was evaluated with Wilcoxon’s rank-sum tests for continuous variables and χ^2^ or Fisher’s exact test for categorical variables. The fold difference is the median biomarker value in the amyloid PET-positive group divided by the median value in the amyloid PET-negative group. All tests were two sided and not adjusted for multiple comparisons.

**Extended Data Table 5:**
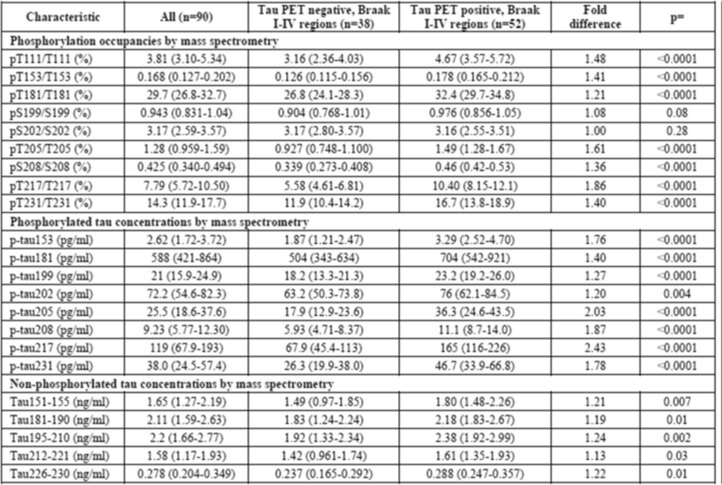
CSF tau measures for the BioFINDER-2 cohort

Continuous values are presented as the median with the IQR. The significance of differences by tau PET status for the Braak I–IV region was evaluated with Wilcoxon’s rank-sum tests for continuous variables and χ^2^ or Fisher’s exact test for categorical variables. The cut-off for tau PET positivity was set at SUVR > 1.32 for the Braak I–IV region. The fold difference is the median biomarker value in the tau PET-positive group divided by the median value in the tau PET-negative group. All tests were two sided and not adjusted for multiple comparisons.

## Data Availability

Knight ADRC data are available to qualified investigators who have a proposal approved by an institutional committee (https://knightadrc.wustl.edu/Research/ResourceRequest.htm) that meets monthly. The study must be approved by an institutional review board to ensure ethical research practices and investigators must agree to the terms and conditions of the data use agreement, which includes not distributing the data without permission. For BioFINDER-2 data, anonymized data will be shared by request from a qualified academic investigator for the sole purpose of replicating procedures and results presented in the article and as long as data transfer is in agreement with EU legislation on the general data protection regulation and decisions by the Ethical Review Board of Sweden and Region Skåne, which should be regulated in a material transfer agreement.
