# Supplementary material for: CSF tau phosphorylation occupancies at T217 and T205 represent improved biomarkers of amyloid and tau pathology in Alzheimer’s disease
Source: Nat Aging. 2023 Mar 13;3(4):391–401. doi: 10.1038/s43587-023-00380-7 (PMC10154225; doi:10.1038/s43587-023-00380-7)
Supplement: Supplementary file 1 — Supplementary Fig. 1, Tables 1–32 and Appendix 1. [file 43587_2023_380_MOESM1_ESM.pdf]

nature aging

Letter

https://doi.org/10.1038/s43587-023-00380-7

CSF tau phosphorylation occupancies at T217 and T205 represent improved biomarkers of amyloid and tau pathology in Alzheimer's disease

In the format provided by the authors and unedited

Supplementary information
Table of Contents

Supplementary figures	page
Supplemental Figure 1. Distribution of tau PET summary measure values.	3
**Participant characteristics**	
Supplemental Table 1. Participant characteristics for the Knight ADRC tau PET symptomatic AD sub-cohort.	4
**Associations with amyloid PET**	
Supplemental Table 2. CSF tau measures for individuals in the Knight ADRC amyloid PET cohort with no missing CSF biomarker measures.	5
Supplemental Table 3. Correspondence of CSF measures with the amyloid PET status in the Knight ADRC amyloid PET cohort.	6
Supplemental Table 4. Correlations between CSF biomarkers and amyloid PET Centiloid in the Knight ADRC amyloid PET cohort.	7
Supplemental Table 5. Correlations between CSF biomarkers and amyloid PET Centiloid for individuals in the Knight ADRC amyloid PET cohort with no missing CSF biomarker measures.	8
Supplemental Table 6. Correlations between CSF biomarkers and amyloid PET Centiloid for amyloid PET positive individuals in the Knight ADRC amyloid PET cohort.	9
**Associations with the tau PET summary measure**	
Supplemental Table 7. CSF tau measures for the Knight ADRC tau PET cohort.	10
Supplemental Table 8. CSF tau measures for individuals in the Knight ADRC tau PET cohort with no missing CSF biomarker measures.	11
Supplemental Table 9. Correspondence of CSF measures with the tau PET summary measure in the Knight ADRC tau PET cohort.	12
Supplemental Table 10. Correspondence of CSF measures with the tau PET summary measure for amyloid PET positive individuals in the Knight ADRC tau PET cohort.	13
Supplemental Table 11. Identification of individuals in the Knight ADRC tau PET cohort who are both tau PET positive and amyloid PET positive.	14
Supplemental Table 12. Correlations between CSF biomarkers and the tau PET summary measure in the Knight ADRC tau PET cohort.	15
Supplemental Table 13. Correlations between CSF biomarkers and the tau PET summary measure for individuals in the Knight ADRC tau PET cohort with no missing CSF biomarker measures.	16
Supplemental Table 14. Correlations between CSF biomarkers and the tau PET summary measure for amyloid PET positive individuals in the Knight ADRC tau PET cohort.	17
**Associations with regional tau PET**	
Supplemental Table 15. Correlations between CSF pT205/T205 and regional tau PET for amyloid PET positive individuals in the Knight ADRC tau PET cohort.	18
Supplemental Table 16. Correlations between CSF pT217/T217 and regional tau PET for amyloid PET positive individuals in the Knight ADRC tau PET cohort.	19
Supplemental Table 17. Correlations between CSF pT181/T181 and regional tau PET for amyloid PET positive individuals in the Knight ADRC tau PET cohort.	20
Supplemental Table 18. Correlations between CSF pT231/T231 and regional tau PET for amyloid PET positive individuals in the Knight ADRC tau PET cohort.	21
**Associations with regional brain volumes**	
Supplemental Table 19. Correlations between CSF pT205/T205 and regional brain volumes for amyloid PET positive individuals in the Knight ADRC amyloid PET cohort.	22
Supplemental Table 20. Correlations between CSF pT217/T217 and regional brain volumes	23

for amyloid PET positive individuals in the Knight ADRC amyloid PET cohort.
Supplemental Table 21. Correlations between CSF pT181/T181 and regional brain volumes for amyloid PET positive individuals in the Knight ADRC amyloid PET cohort. 24
Supplemental Table 22. Correlations between CSF pT231/T231 and regional brain volumes for amyloid PET positive individuals in the Knight ADRC amyloid PET cohort. 25

Associations with clinical dementia symptoms

Supplemental Table 23. Correspondence of CSF measures with clinical status in the Knight ADRC amyloid PET cohort. 26
Supplemental Table 24. Correspondence of CSF measures with clinical status in the amyloid PET positive individuals in the Knight ADRC amyloid PET cohort. 27
Supplemental Table 25. Correlations between biomarker measures and Clinical Dementia Rating Sum of Boxes for the Knight ADRC amyloid PET cohort. 28
Supplemental Table 26. Correlations between biomarker measures and Clinical Dementia Rating Sum of Boxes for amyloid PET positive individuals in the Knight ADRC amyloid PET cohort. 29

Associations in cognitively impaired individuals

Supplemental Table 27. CSF tau measures for cognitively impaired individuals in the Knight ADRC tau PET cohort. 30
Supplemental Table 28. Correlations between CSF biomarkers and amyloid PET Centiloid for cognitively impaired individuals in the Knight ADRC tau PET cohort. 31
Supplemental Table 29. Correlations between CSF biomarkers and the tau PET summary measure for cognitively impaired individuals in the Knight ADRC tau PET cohort. 32
Supplemental Table 30. Correlations between CSF biomarkers and amyloid PET Centiloid for individuals in the BioFINDER-2 cohort. 33
Supplemental Table 31. Correlations between CSF biomarkers and tau PET for Braak I-IV regions for individuals in BioFINDER-2 cohort. 34
Supplemental Table 32. Correlations between CSF biomarkers and tau PET for Braak V-VI regions for individuals in BioFINDER-2 cohort. 35

Appendix

Appendix 1. Assay methodology, quality control, and performance. 36

Supplemental Figure 1. Distribution of tau PET summary measure values. Data from the baseline tau PET scan were examined from individuals enrolled in studies at the Knight Alzheimer Disease Research Center. This data overlaps with tau PET cohort (**Supplemental Table 1**), but two additional individuals were included in this analysis who did not have an amyloid PET scan and/or CSF data. At the time of the baseline tau PET scan, the individuals had the following Clinical Dementia Rating (CDR): CDR 0, n=314; CDR 0.5, n=46; CDR 1, n=12; CDR 2, n=1. The tau PET summary measure was calculated as described in the methods. Gaussian mixture modeling was implemented in R using the mclust software package and found a normal (blue dashed line) and abnormal (red dashed line) cluster. The value at which there was equal probability of belonging to either group was 1.52 (black dashed line), which was used as the cut-off value for tau PET positivity.

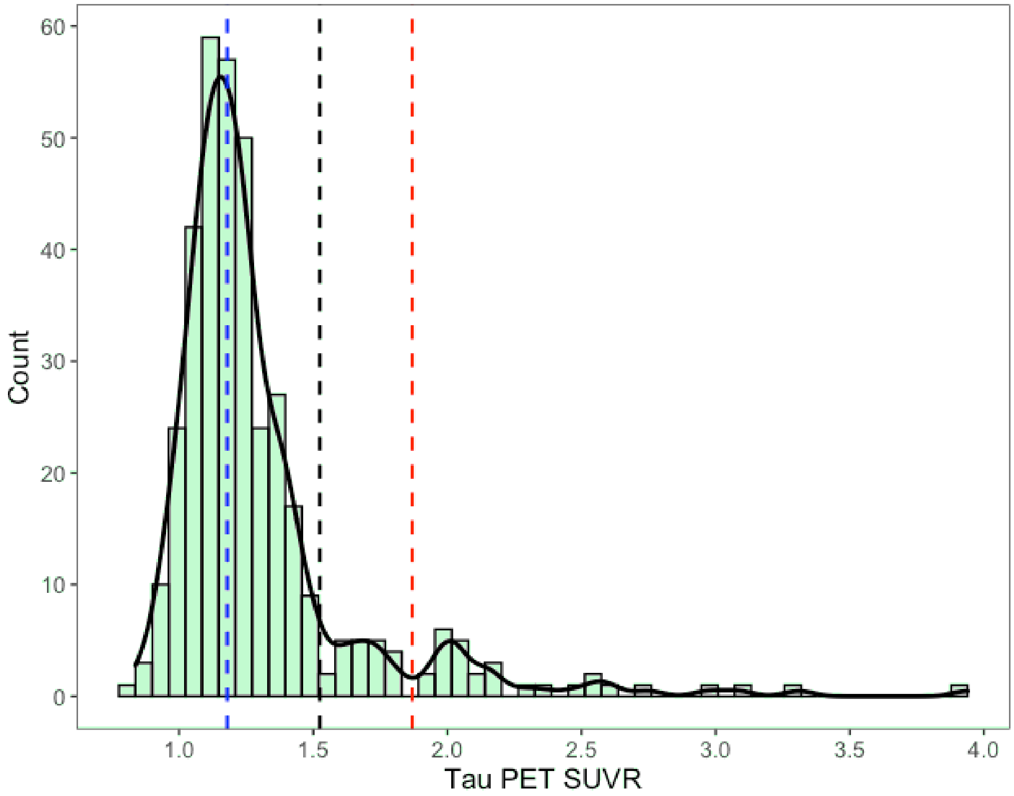

Supplemental Table 1. Participant characteristics for the Knight ADRC tau PET symptomatic AD sub-cohort. Continuous values are presented as the median with the interquartile range. The significance of differences by tau PET status were evaluated with Wilcoxon ranked sum tests for continuous variables and Chi-Square or Fisher exact tests for categorical variables. All tests were two-sided and were not adjusted for multiple comparisons.

Characteristic	All (n=55)	Tau PET Negative (n=20)	Tau PET Positive (n=35)	p=
**Demographics**				
Age at CSF collection (years)	75.5 (70.9-79.2)	72.4 (71.3-77.2)	75.9 (70.6-79.2)	0.58
Gender (n, % female)	23, 42%	5, 25%	18, 51%	0.06
APOE ε4 carrier status (n, % ε4 carrier)	30, 55%	8, 40%	22, 63%	0.14
CDR (0/0.5/1+)	0/41/14	0/22/13	0/19/1	0.03
CDR-SB	2.5 (1.5-4)	2 (1.5-2.5)	3.5 (1.5-5)	0.02
Years of education	16 (13-18)	16 (12-18)	16 (13-18)	0.91
**CSF Lumipulse measures**				
Aβ42 (pg/ml)	526 (365-640)	738 (528-927)	471 (323-535)	<0.0001
Aβ40 (pg/ml)	11600 (8870-13400)	12700 (9950-14200)	11200 (8800-13200)	0.08
Aβ42/Aβ40	0.0457 (0.0383-0.0539)	0.0575 (0.0457-0.0703)	0.0438 (0.0359-0.0468)	<0.0001
Total tau (pg/ml)	509 (414-686)	443 (320-506)	623 (453-710)	0.006
p-tau181 (pg/ml)	74.9 (55.2-98.3)	55.2 (35.3-71.4)	92.6 (68.5-109)	0.0002
**Amyloid and tau PET measures**				
Amyloid PET status (n, % positive)	45, 82%	10, 50%	35, 100%	<0.0001
Amyloid PET Centiloid	78.7 (47.1-100)	19.2 (3.4-55)	88.2 (77.1-107)	<0.0001
Interval between CSF collection and amyloid PET (years)	0.19 (0.05-0.31)	0.15 (0.05-0.29)	0.19 (0.04-0.33)	0.98
Tau PET Summary Measure	1.82 (1.29-2.13)	1.23 (1.14-1.33)	2.04 (1.95-2.28)	<0.0001
Interval between CSF collection and tau PET (years)	0.20 (0.06-0.29)	0.20 (0.05-0.35)	0.194 (0.06-0.25)	0.42

Abbreviations: CSF, cerebrospinal fluid; CDR, Clinical Dementia Rating; CDR-SB, Clinical Dementia Rating Sum of Boxes; PET, positron emission tomography; PIB, ^{11}C-Pittsburgh Compound B; SUVR, standardized uptake value ratio.

Supplemental Table 2. CSF tau measures for individuals in the Knight ADRC amyloid PET cohort with no missing CSF biomarker measures. Continuous values are presented as the median with the interquartile range. The significance of differences by amyloid PET status were evaluated with Wilcoxon ranked sum tests for continuous variables and Chi-Square or Fisher exact tests for categorical variables. The fold difference is the median biomarker value in the amyloid PET positive group divided by the median value in the amyloid PET negative group. All tests were two-sided and were not adjusted for multiple comparisons.

Characteristic	Entire cohort (n=554)	Amyloid PET negative (n=323)	Amyloid PET positive (n=231)	Fold difference	p=
**Phosphorylation occupancies by mass spectrometry**					
pT111/T111 (%)	3.73 (2.46-7.49)	2.72 (2.13-3.37)	8.13 (6.15-10.4)	2.99	<0.0001
pT153/T153 (%)	0.0634 (0.0335-0.129)	0.0371 (0.0216-0.0599)	0.139 (0.0996-0.195)	3.75	<0.0001
pT175/T175 (%)	0.473 (0.395-0.552)	0.491 (0.415-0.559)	0.456 (0.376-0.533)	0.93	0.0001
pT181/T181 (%)	30.1 (27.9-34.6)	28.5 (27.1-29.9)	35.5 (32.2-39.6)	1.25	<0.0001
pS199/S199 (%)	0.682 (0.551-0.877)	0.639 (0.51-0.808)	0.741 (0.626-0.932)	1.16	<0.0001
pS202/S202 (%)	5.44 (4.51-6.39)	5.66 (4.74-6.65)	5.02 (4.22-6)	0.89	<0.0001
pT205/T205 (%)	1.01 (0.829-1.29)	0.896 (0.757-1.06)	1.29 (1.02-1.59)	1.44	<0.0001
pS208/S208 (%)	0.15 (0.092-0.263)	0.106 (0.0752-0.145)	0.276 (0.208-0.35)	2.60	<0.0001
pT217/T217 (%)	4.05 (3.12-8.41)	3.23 (2.91-3.59)	9.4 (6.68-12.7)	2.91	<0.0001
pT231/T231 (%)	9.86 (4.38-22.8)	5.7 (2.37-9.23)	24.9 (15.6-31.3)	4.37	<0.0001
**Phosphorylated tau concentrations by mass spectrometry**					
p-tau153 (pg/ml)	1.3 (0.622-3.35)	0.726 (0.378-1.17)	4.02 (2.16-6.58)	5.54	<0.0001
p-tau175 (pg/ml)	11.7 (8.59-15.9)	10.5 (8.09-14.2)	13.6 (9.79-17.3)	1.30	<0.0001
p-tau181 (pg/ml)	747 (554-1110)	629 (478-787)	1080 (783-1400)	1.72	<0.0001
p-tau199 (pg/ml)	18.5 (13.3-26)	15.9 (10.9-20.2)	25.5 (18.3-32.8)	1.60	<0.0001
p-tau202 (pg/ml)	145 (118-181)	132 (111-158)	164 (136-201)	1.24	<0.0001
p-tau205 (pg/ml)	25.4 (19.4-38.9)	21.2 (17.3-25.9)	41 (29.4-58.3)	1.93	<0.0001
p-tau208 (pg/ml)	3.77 (2.16-7.82)	2.49 (1.62-3.58)	8.98 (5.69-13.3)	3.61	<0.0001
p-tau217 (pg/ml)	79.3 (49.7-180)	53.6 (41.7-71.8)	200 (124-294)	3.73	<0.0001
p-tau231 (pg/ml)	23.2 (8.59-68.8)	10.7 (4.14-21.2)	75.6 (43.9-130)	7.07	<0.0001
**Non-phosphorylated tau concentrations by mass spectrometry**					
Tau151-155 (ng/ml)	2.17 (1.7-3.03)	1.94 (1.55-2.38)	2.81 (2.15-3.49)	1.45	<0.0001
Tau181-190 (ng/ml)	2.48 (1.92-3.27)	2.2 (1.75-2.77)	3.02 (2.33-3.7)	1.37	<0.0001
Tau195-210 (ng/ml)	2.65 (2.08-3.53)	2.38 (1.87-2.89)	3.36 (2.58-4.17)	1.41	<0.0001
Tau212-221 (ng/ml)	1.8 (1.43-2.38)	1.65 (1.31-2.01)	2.14 (1.69-2.69)	1.30	<0.0001
Tau226-230 (ng/ml)	0.239 (0.18-0.339)	0.203 (0.158-0.259)	0.325 (0.239-0.442)	1.60	<0.0001

Supplemental Table 3. Correspondence of CSF measures with the amyloid PET status in the Knight ADRC amyloid PET cohort. The receiver operating characteristic area under the curve of CSF measures with amyloid PET status is shown with 95% confidence intervals. The cut-off for the CSF measures that best distinguished amyloid PET status, as well as the positive percent agreement (PPA) and negative percent agreement (NPA) of the cut-off for amyloid PET status, are shown. CSF measures are listed in order of correspondence with amyloid PET status, stratified by measure type.

Analyte	AUC (95% CI)	Cut-off	PPA	NPA
**CSF Lumipulse measures**				
Aβ42/Aβ40	0.97 (0.95-0.98)	0.0673	0.97	0.88
p-tau181 (pg/ml)	0.89 (0.86-0.91)	42.9	0.83	0.82
Aβ42 (pg/ml)	0.87 (0.84-0.89)	661	0.82	0.80
Total tau (pg/ml)	0.83 (0.80-0.86)	338	0.75	0.79
Aβ40 (pg/ml)	0.56 (0.52-0.60)	10801	0.59	0.54
**Phosphorylation occupancies by mass spectrometry**				
pT217/T217 (%)	0.98 (0.97-0.99)	4.51	0.96	0.93
pT111/T111 (%)	0.96 (0.94-0.97)	4.41	0.84	0.94
pT231/T231 (%)	0.93 (0.91-0.95)	12.8	0.84	0.92
pT153/T153 (%)	0.93 (0.90-0.95)	0.0770	0.84	0.90
pS208/S208 (%)	0.92 (0.90-0.94)	0.178	0.82	0.89
pT181/T181 (%)	0.91 (0.89-0.94)	31.4	0.79	0.91
pT205/T205 (%)	0.80 (0.77-0.84)	1.08	0.72	0.77
pS202/S202 (%)	0.66 (0.61-0.70)	5.19	0.56	0.70
pS199/S199 (%)	0.63 (0.59-0.67)	0.611	0.76	0.48
pT175/T175 (%)	0.54 (0.49-0.58)	0.486	0.64	0.46
**Phosphorylated tau concentrations by mass spectrometry**				
p-tau217 (pg/ml)	0.95 (0.93-0.96)	81.8	0.90	0.87
p-tau231 (pg/ml)	0.94 (0.92-0.95)	34.9	0.81	0.93
p-tau208 (pg/ml)	0.93 (0.91-0.95)	3.76	0.90	0.82
p-tau153 (pg/ml)	0.92 (0.90-0.94)	1.72	0.82	0.89
p-tau205 (pg/ml)	0.88 (0.85-0.91)	28.3	0.75	0.87
p-tau181 (pg/ml)	0.83 (0.80-0.86)	825	0.70	0.84
p-tau199 (pg/ml)	0.78 (0.75-0.82)	18.2	0.73	0.70
p-tau202 (pg/ml)	0.73 (0.69-0.76)	139	0.73	0.64
p-tau175 (pg/ml)	0.67 (0.63-0.71)	11.6	0.65	0.67
**Non-phosphorylated tau concentrations by mass spectrometry**				
Tau151-155 (ng/ml)	0.79 (0.75-0.82)	2.18	0.72	0.74
Tau195-210 (ng/ml)	0.78 (0.75-0.82)	2.75	0.68	0.77
Tau226-230 (ng/ml)	0.78 (0.75-0.82)	0.236	0.74	0.71
Tau181-190 (ng/ml)	0.75 (0.72-0.79)	2.53	0.67	0.74
Tau212-221 (ng/ml)	0.74 (0.71-0.78)	1.82	0.67	0.72

Supplemental Table 4. Correlations between CSF biomarkers and amyloid PET Centiloid in the Knight ADRC amyloid PET cohort. The Spearman correlation and partial Spearman correlation after adjusting for age and sex are shown. CSF measures are shown in order of the absolute value of their correlation with amyloid PET Centiloid, stratified by measure type. All tests were two-sided and were not adjusted for multiple comparisons.

Measure	n=	Spearman ρ	p=	Partial Spearman ρ	p=
**CSF Lumipulse measures**					
Aβ42/Aβ40	750	-0.74 (-0.77 to -0.71)	<0.0001	-0.71 (-0.74 to -0.67)	<0.0001
p-tau181 (pg/ml)	750	0.61 (0.57 to 0.65)	<0.0001	0.56 (0.51 to 0.61)	<0.0001
Aβ42 (pg/ml)	750	-0.56 (-0.61 to -0.51)	<0.0001	-0.54 (-0.59 to -0.49)	<0.0001
Total tau (pg/ml)	750	0.51 (0.45 to 0.56)	<0.0001	0.44 (0.38 to 0.50)	<0.0001
Aβ40 (pg/ml)	750	0.09 (0.02 to 0.16)	0.01	0.05 (-0.02 to 0.12)	0.15
**Phosphorylation occupancies by mass spectrometry**					
pT217/T217 (%)	750	0.76 (0.73 to 0.79)	<0.0001	0.74 (0.70 to 0.77)	<0.0001
pT111/T111 (%)	746	0.72 (0.68 to 0.75)	<0.0001	0.69 (0.65 to 0.73)	<0.0001
pT231/T231 (%)	620	0.70 (0.66 to 0.74)	<0.0001	0.67 (0.63 to 0.71)	<0.0001
pT153/T153 (%)	660	0.70 (0.65 to 0.73)	<0.0001	0.66 (0.62 to 0.70)	<0.0001
pS208/S208 (%)	685	0.68 (0.64 to 0.72)	<0.0001	0.66 (0.61 to 0.70)	<0.0001
pT181/T181 (%)	747	0.66 (0.62 to 0.70)	<0.0001	0.63 (0.59 to 0.67)	<0.0001
pT205/T205 (%)	749	0.47 (0.41 to 0.52)	<0.0001	0.42 (0.35 to 0.47)	<0.0001
pS202/S202 (%)	750	-0.25 (-0.31 to -0.18)	<0.0001	0.16 (0.09 to 0.23)	<0.0001
pS199/S199 (%)	743	0.19 (0.12 to 0.26)	<0.0001	-0.22 (-0.28 to -0.15)	<0.0001
pT175/T175 (%)	734	-0.07 (-0.14 to 0.00)	0.05	-0.07 (-0.14 to 0.00)	0.05
**Phosphorylated tau concentrations by mass spectrometry**					
p-tau217 (pg/ml)	750	0.71 (0.67 to 0.74)	<0.0001	0.67 (0.62 to 0.70)	<0.0001
p-tau231 (pg/ml)	619	0.71 (0.67 to 0.75)	<0.0001	0.67 (0.63 to 0.71)	<0.0001
p-tau208 (pg/ml)	685	0.70 (0.67 to 0.74)	<0.0001	0.67 (0.62 to 0.71)	<0.0001
p-tau153 (pg/ml)	660	0.69 (0.65 to 0.73)	<0.0001	0.65 (0.60 to 0.69)	<0.0001
p-tau205 (pg/ml)	749	0.59 (0.54 to 0.64)	<0.0001	0.53 (0.48 to 0.58)	<0.0001
p-tau181 (pg/ml)	747	0.52 (0.46 to 0.57)	<0.0001	0.46 (0.40 to 0.51)	<0.0001
p-tau199 (pg/ml)	743	0.44 (0.38 to 0.50)	<0.0001	0.37 (0.31 to 0.44)	<0.0001
p-tau202 (pg/ml)	750	0.36 (0.30 to 0.42)	<0.0001	0.29 (0.22 to 0.35)	<0.0001
p-tau175 (pg/ml)	734	0.26 (0.19 to 0.33)	<0.0001	0.20 (0.13 to 0.27)	<0.0001
**Non-phosphorylated tau concentrations by mass spectrometry**					
Tau226-230 (ng/ml)	719	0.46 (0.40 to 0.51)	<0.0001	0.40 (0.34 to 0.46)	<0.0001
Tau151-155 (ng/ml)	747	0.45 (0.39 to 0.51)	<0.0001	0.38 (0.32 to 0.44)	<0.0001
Tau195-210 (ng/ml)	750	0.45 (0.39 to 0.50)	<0.0001	0.38 (0.32 to 0.44)	<0.0001
Tau181-190 (ng/ml)	750	0.40 (0.34 to 0.46)	<0.0001	0.32 (0.26 to 0.39)	<0.0001
Tau212-221 (ng/ml)	750	0.38 (0.32 to 0.44)	<0.0001	0.38 (0.32 to 0.44)	<0.0001

Supplemental Table 5. Correlations between CSF biomarkers and amyloid PET Centiloid for individuals in the Knight ADRC amyloid PET cohort with no missing CSF biomarker measures. The Spearman correlation and partial Spearman correlation after adjusting for age and sex are shown. CSF measures are shown in order of the absolute value of their correlation with amyloid PET Centiloid, stratified by measure type. All tests were two-sided and were not adjusted for multiple comparisons.

Measure	Spearman ρ	p=	Partial Spearman ρ	p=
**CSF Lumipulse measures**				
Aβ42/Aβ40	-0.78 (-0.81 to -0.75)	<0.0001	-0.76 (-0.79 to -0.72)	<0.0001
Aβ42 (pg/ml)	-0.66 (-0.70 to -0.60)	<0.0001	-0.63 (-0.68 to -0.58)	<0.0001
p-tau181 (pg/ml)	0.63 (0.57 to 0.67)	<0.0001	0.58 (0.52 to 0.63)	<0.0001
Total tau (pg/ml)	0.50 (0.43 to 0.56)	<0.0001	0.43 (0.36 to 0.50)	<0.0001
Aβ40 (pg/ml)	0.01 (-0.07 to 0.09)	0.79	-0.02 (-0.10 to 0.06)	0.63
**Phosphorylation occupancies by mass spectrometry**				
pT217/T217 (%)	0.81 (0.78 to 0.83)	<0.0001	0.78 (0.75 to 0.81)	<0.0001
pT111/T111 (%)	0.76 (0.73 to 0.80	<0.0001	0.74 (0.70 to 0.78)	<0.0001
pT153/T153 (%)	0.72 (0.68 to 0.76)	<0.0001	0.69 (0.65 to 0.74)	<0.0001
pT231/T231 (%)	0.72 (0.67 to 0.76)	<0.0001	0.69 (0.64 to 0.73)	<0.0001
pS208/S208 (%)	0.71 (0.67 to 0.75)	<0.0001	0.68 (0.63 to 0.72)	<0.0001
pT181/T181 (%)	0.70 (0.66 to 0.74)	<0.0001	0.68 (0.64 to 0.72)	<0.0001
pT205/T205 (%)	0.54 (0.47 to 0.59)	<0.0001	0.49 (0.43 to 0.55)	<0.0001
pS199/S199 (%)	0.21 (0.13 to 0.29)	<0.0001	0.18 (0.09 to 0.26)	<0.0001
pS202/S202 (%)	-0.20 (-0.28 to -0.12)	<0.0001	-0.18 (-0.26 to -0.09)	<0.0001
pT175/T175 (%)	-0.17 (-0.25 to -0.09)	<0.0001	-0.15 (-0.23 to -0.07)	0.0004
**Phosphorylated tau concentrations by mass spectrometry**				
p-tau217 (pg/ml)	0.74 (0.70 to 0.77)	<0.0001	0.70 (0.66 to 0.74)	<0.0001
p-tau231 (pg/ml)	0.72 (0.68 to 0.76)	<0.0001	0.69 (0.64 to 0.73)	<0.0001
p-tau208 (pg/ml)	0.72 (0.67 to 0.76)	<0.0001	0.68 (0.63 to 0.72)	<0.0001
p-tau153 (pg/ml)	0.71 (0.66 to 0.74)	<0.0001	0.67 (0.62 to 0.71)	<0.0001
p-tau205 (pg/ml)	0.62 (0.57 to 0.67)	<0.0001	0.56 (0.50 to 0.62)	<0.0001
p-tau181 (pg/ml)	0.51 (0.45 to 0.57)	<0.0001	0.45 (0.39 to 0.52)	<0.0001
p-tau199 (pg/ml)	0.45 (0.38 to 0.51)	<0.0001	0.38 (0.31 to 0.45)	<0.0001
p-tau202 (pg/ml)	0.36 (0.29 to 0.43)	<0.0001	0.29 (0.21 to 0.36)	<0.0001
p-tau175 (pg/ml)	0.20 (0.12 to 0.28)	<0.0001	0.15 (0.07 to 0.23)	0.0004
**Non-phosphorylated tau concentrations by mass spectrometry**				
Tau226-230 (ng/ml)	0.48 (0.41 to 0.54)	<0.0001	0.41 (0.34 to 0.48)	<0.0001
Tau151-155 (ng/ml)	0.42 (0.35 to 0.49)	<0.0001	0.36 (0.28 to 0.43)	<0.0001
Tau195-210 (ng/ml)	0.42 (0.35 to 0.49)	<0.0001	0.36 (0.28 to 0.43)	<0.0001
Tau181-190 (ng/ml)	0.36 (0.29 to 0.43)	<0.0001	0.29 (0.22 to 0.37)	<0.0001
Tau212-221 (ng/ml)	0.34 (0.26 to 0.41)	<0.0001	0.26 (0.19 to 0.34)	<0.0001

Supplemental Table 6. Correlations between CSF biomarkers and amyloid PET Centiloid for amyloid PET positive individuals in the Knight ADRC amyloid PET cohort. The Spearman correlation and partial Spearman correlation after adjusting for age and sex are shown. CSF measures are shown in order of the absolute value of their correlation with amyloid PET Centiloid, stratified by measure type. All tests were two-sided and were not adjusted for multiple comparisons.

Measure	n=	Spearman ρ	p=	Partial Spearman ρ	p=
**CSF Lumipulse measures**					
p-tau181 (pg/ml)	263	0.44 (0.34 to 0.53)	<0.0001	0.43 (0.32 to 0.52)	<0.0001
Aβ42/Aβ40	263	-0.42 (-0.52 to -0.32)	<0.0001	-0.41 (-0.50 to -0.30)	<0.0001
Aβ42 (pg/ml)	263	-0.41 (-0.51 to -0.31)	<0.0001	-0.41 (-0.51 to -0.31)	<0.0001
Total tau (pg/ml)	263	0.29 (0.17 to 0.40)	<0.0001	0.28 (0.16 to 0.39)	<0.0001
Aβ40 (pg/ml)	263	-0.13 (-0.25 to -0.01)	0.03	-0.15 (-0.26 to -0.02)	0.02
**Phosphorylation occupancies by mass spectrometry**					
pT217/T217 (%)	263	0.69 (0.62 to 0.75)	<0.0001	0.68 (0.61 to 0.74)	<0.0001
pT111/T111 (%)	263	0.57 (0.48 to 0.65)	<0.0001	0.57 (0.48 to 0.64)	<0.0001
pT181/T181 (%)	262	0.54 (0.45 to 0.62)	<0.0001	0.54 (0.45 to 0.62)	<0.0001
pT205/T205 (%)	263	0.53 (0.44 to 0.61)	<0.0001	0.53 (0.44 to 0.61)	<0.0001
pS208/S208 (%)	260	0.49 (0.40 to 0.58)	<0.0001	0.49 (0.39 to 0.58)	<0.0001
pT231/T231 (%)	240	0.44 (0.34 to 0.54)	<0.0001	0.44 (0.34 to 0.54)	<0.0001
pT153/T153 (%)	261	0.44 (0.34 to 0.53)	<0.0001	0.45 (0.34 to 0.54)	<0.0001
pT175/T175 (%)	257	-0.14 (-0.25 to -0.01)	0.03	-0.17 (-0.28 to -0.04)	0.007
pS202/S202 (%)	263	-0.11 (-0.23 to 0.01)	0.07	-0.11 (-0.23 to 0.01)	0.07
pS199/S199 (%)	263	0.05 (-0.07 to 0.17)	0.42	0.06 (-0.07 to 0.18)	0.36
**Phosphorylated tau concentrations by mass spectrometry**					
p-tau205 (pg/ml)	263	0.55 (0.46 to 0.63)	<0.0001	0.54 (0.45 to 0.62)	<0.0001
p-tau217 (pg/ml)	263	0.54 (0.45 to 0.62)	<0.0001	0.53 (0.44 to 0.62)	<0.0001
p-tau208 (pg/ml)	260	0.50 (0.41 to 0.59)	<0.0001	0.49 (0.40 to 0.58)	<0.0001
p-tau231 (pg/ml)	239	0.44 (0.33 to 0.54)	<0.0001	0.44 (0.33 to 0.54)	<0.0001
p-tau153 (pg/ml)	261	0.43 (0.32 to 0.52)	<0.0001	0.42 (0.32 to 0.52)	<0.0001
p-tau181 (pg/ml)	262	0.33 (0.22 to 0.44)	<0.0001	0.32 (0.20 to 0.42)	<0.0001
p-tau202 (pg/ml)	263	0.26 (0.15 to 0.37)	<0.0001	0.25 (0.13 to 0.36)	<0.0001
p-tau199 (pg/ml)	263	0.25 (0.14 to 0.36)	<0.0001	0.25 (0.13 to 0.36)	<0.0001
p-tau175 (pg/ml)	257	0.05 (-0.07 to 0.18)	0.39	0.03 (-0.09 to 0.15)	0.63
**Non-phosphorylated tau concentrations by mass spectrometry**					
Tau195-210 (ng/ml)	263	0.26 (0.15 to 0.37)	<0.0001	0.25 (0.13 to 0.36)	<0.0001
Tau151-155 (ng/ml)	261	0.23 (0.11 to 0.34)	0.0002	0.21 (0.09 to 0.32)	0.0006
Tau181-190 (ng/ml)	263	0.19 (0.07 to 0.30)	0.002	0.18 (0.06 to 0.29)	0.004
Tau226-230 (ng/ml)	259	0.19 (0.07 to 0.31)	0.0016	0.19 (0.07 to 0.31)	0.002
Tau212-221 (ng/ml)	263	0.17 (0.05 to 0.29)	0.005	0.16 (0.04 to 0.27)	0.01

Supplemental Table 7. CSF tau measures for the Knight ADRC tau PET cohort. Continuous values are presented as the median with the interquartile range. The significance of differences by tau PET status were evaluated with Wilcoxon ranked sum tests for continuous variables and Chi-Square or Fisher exact tests for categorical variables. The fold difference is the median biomarker value in the tau PET positive group divided by the median value in the tau PET negative group. All tests were two-sided and were not adjusted for multiple comparisons.

**Characteristic**	**Entire cohort (n=371)**		**Tau PET negative (n=319)**		**Tau PET positive (n=52)**		**Fold change**	**p=**
**Phosphorylation occupancies by mass spectrometry**								
pT111/T111 (%)	369	3.30 (2.35-6.51)	317	3.01 (2.25-4.45)	52	9.73 (8.44-11.4)	3.23	<0.0001
pT153/T153 (%)	326	0.0601 (0.0319-0.118)	274	0.048 (0.0277-0.0803)	52	0.163 (0.119-0.21)	3.40	<0.0001
pT175/T175 (%)	363	0.458 (0.377-0.554)	311	0.465 (0.381-0.559)	52	0.427 (0.367-0.516)	0.92	0.11
pT181/T181 (%)	370	29.6 (27.4-32.4)	318	28.9 (27.1-31)	52	36.8 (35-40.3)	1.27	<0.0001
pS199/S199 (%)	368	0.674 (0.528-0.867)	316	0.663 (0.515-0.829)	52	0.774 (0.649-0.975)	1.17	0.0007
pS202/S202 (%)	371	5.61 (4.68-6.54)	319	5.66 (4.86-6.67)	52	4.76 (4.22-5.95)	0.84	0.0002
pT205/T205 (%)	371	0.959 (0.786-1.21)	319	0.907 (0.763-1.12)	52	1.54 (1.37-1.81)	1.70	<0.0001
pS208/S208 (%)	332	0.143 (0.0888-0.247)	280	0.126 (0.0812-0.185)	52	0.301 (0.259-0.361)	2.39	<0.0001
pT217/T217 (%)	371	3.52 (3.03-7.02)	319	3.32 (2.97-4.64)	52	12.1 (10.6-13.6)	3.64	<0.0001
pT231/T231 (%)	298	8.54 (3.83-21.2)	249	6.75 (2.88-12.7)	49	27.4 (24.3-33.7)	4.06	<0.0001
**Phosphorylated tau concentrations by mass spectrometry**								
p-tau153 (pg/ml)	326	1.08 (0.537-3.26)	274	0.912 (0.482-1.79)	52	4.81 (3.89-7.57)	5.27	<0.0001
p-tau175 (pg/ml)	363	11.0 (6.96-14.7)	311	10.5 (6.5-13.8)	52	13.9 (10.9-17.2)	1.32	<0.0001
p-tau181 (pg/ml)	370	661 (477-1010)	318	631 (463-833)	52	1210 (957-1530)	1.92	<0.0001
p-tau199 (pg/ml)	368	16.9 (11.5-24)	316	15.7 (10.6-21.1)	52	27.7 (23.1-39.6)	1.76	<0.0001
p-tau202 (pg/ml)	371	138 (111-168)	319	131 (109-158)	52	185 (144-224)	1.41	<0.0001
p-tau205 (pg/ml)	371	22.6 (16.9-34.3)	319	21.3 (16.1-27.1)	52	59.1 (44.4-74.3)	2.77	<0.0001
p-tau208 (pg/ml)	332	3.51 (1.94-7.28)	280	2.90 (1.71-4.96)	52	11.3 (9.04-15.6)	3.90	<0.0001
p-tau217 (pg/ml)	371	59.5 (43.2-143)	319	54.5 (39.7-87.9)	52	270 (212-385)	4.95	<0.0001
p-tau231 (pg/ml)	297	19.6 (7.18-64.7)	248	14.1 (5.98-33.1)	49	101 (74.7-147)	7.16	<0.0001
**Non-phosphorylated tau concentrations by mass spectrometry**								
Tau151-155 (ng/ml)	369	1.97 (1.5-2.72)	317	1.88 (1.41-2.44)	52	3.10 (2.44-3.67)	1.65	<0.0001
Tau181-190 (ng/ml)	371	2.27 (1.76-3.09)	319	2.17 (1.66-2.79)	52	3.37 (2.59-3.95)	1.55	<0.0001
Tau195-210 (ng/ml)	371	2.44 (1.87-3.32)	319	2.31 (1.76-2.94)	52	3.78 (2.95-4.53)	1.64	<0.0001
Tau212-221 (ng/ml)	371	1.69 (1.28-2.23)	319	1.59 (1.20-2.06)	52	2.35 (1.85-2.79)	1.48	<0.0001
Tau226-230 (ng/ml)	351	0.228 (0.158-0.327)	299	0.206 (0.147-0.286)	52	0.343 (0.291-0.506)	1.67	<0.0001

Supplemental Table 8. CSF tau measures for individuals in the Knight ADRC tau PET cohort with no missing CSF biomarker measures. Continuous values are presented as the median with the interquartile range. The significance of differences by tau PET status were evaluated with Wilcoxon ranked sum tests for continuous variables and Chi-Square or Fisher exact tests for categorical variables. The fold difference is the median biomarker value in the amyloid PET positive group divided by the median value in the amyloid PET negative group. All tests were two-sided and were not adjusted for multiple comparisons.

Characteristic	Entire cohort (n=264)	Tau PET negative (n=215)	Tau PET positive (n=49)	Fold difference	p=
**Phosphorylation occupancies by mass spectrometry**					
pT111/T111 (%)	3.8 (2.53-8.13)	3.21 (2.4-5.07)	9.65 (8.44-11.1)	3.01	<0.0001
pT153/T153 (%)	0.0626 (0.0338-0.124)	0.0498 (0.0303-0.0848)	0.163 (0.123-0.21)	3.27	<0.0001
pT175/T175 (%)	0.47 (0.398-0.566)	0.491 (0.405-0.572)	0.427 (0.367-0.532)	0.87	0.007
pT181/T181 (%)	30.2 (28.2-35)	29.5 (27.8-31.8)	36.8 (35-40.3)	1.25	<0.0001
pS199/S199 (%)	0.693 (0.554-0.887)	0.678 (0.545-0.828)	0.774 (0.649-0.976)	1.14	0.003
pS202/S202 (%)	5.44 (4.62-6.41)	5.57 (4.7-6.62)	4.76 (4.22-5.98)	0.85	0.004
pT205/T205 (%)	1.01 (0.819-1.3)	0.94 (0.787-1.14)	1.54 (1.37-1.81)	1.64	<0.0001
pS208/S208 (%)	0.15 (0.0962-0.269)	0.132 (0.0884-0.193)	0.301 (0.265-0.373)	2.28	<0.0001
pT217/T217 (%)	4.03 (3.17-8.93)	3.46 (3.06-5.4)	12.1 (10.6-13.8)	3.50	<0.0001
pT231/T231 (%)	9.78 (4.24-23.7)	7.66 (3.61-13.6)	27.4 (24.3-33.7)	3.58	<0.0001
p-tau153 (pg/ml)	1.22 (0.602-3.71)	0.997 (0.535-2.12)	4.81 (3.89-7.67)	4.82	<0.0001
p-tau175 (pg/ml)	11.9 (8.74-15.9)	11.6 (8.39-15)	14.0 (11.6-18.1)	1.21	0.0005
p-tau181 (pg/ml)	747 (556-1130)	676 (521-937)	1210 (957-1530)	1.79	<0.0001
p-tau199 (pg/ml)	19.4 (13.5-26.6)	17.3 (12.3-23.0)	28.4 (23.8-40.0)	1.64	<0.0001
p-tau202 (pg/ml)	146 (121-180)	142 (114-164)	185 (144-227)	1.30	<0.0001
p-tau205 (pg/ml)	25 (19.8-40.1)	22.7 (18.5-30.1)	59.1 (44.9-74.4)	2.60	<0.0001
p-tau208 (pg/ml)	3.73 (2.29-8.59)	3.21 (1.94-5.48)	11.3 (9.24-15.6)	3.52	<0.0001
p-tau217 (pg/ml)	78.1 (49.6-188)	63.3 (45.8-111)	270 (226-401)	4.27	<0.0001
p-tau231 (pg/ml)	23.2 (8.61-74.5)	15.9 (6.86-35.0)	101 (74.7-147)	6.35	<0.0001
Tau151-155 (ng/ml)	2.16 (1.70-3.04)	2.05 (1.60-2.67)	3.10 (2.46-3.67)	1.51	<0.0001
Tau181-190 (ng/ml)	2.50 (1.97-3.37)	2.29 (1.87-3.08)	3.37 (2.74-3.95)	1.47	<0.0001
Tau195-210 (ng/ml)	2.69 (2.10-3.64)	2.46 (1.99-3.22)	3.78 (2.96-4.53)	1.54	<0.0001
Tau212-221 (ng/ml)	1.83 (1.43-2.47)	1.73 (1.37-2.24)	2.38 (1.90-2.82)	1.38	<0.0001
Tau226-230 (ng/ml)	0.243 (0.182-0.337)	0.228 (0.175-0.306)	0.343 (0.291-0.506)	1.50	<0.0001

Supplemental Table 9. Correspondence of CSF measures with the tau PET summary measure in the Knight ADRC tau PET cohort. The receiver operating characteristic area under the curve of CSF measures with tau PET status is shown with 95% confidence intervals. The cut-off for the CSF measures that best distinguished tau PET status, as well as the positive percent agreement (PPA) and negative percent agreement (NPA) of the cut-off for tau PET status, are shown. CSF measures are listed in order of correspondence with tau PET status, stratified by measure type.

Analyte	AUC (95% CI)	Cut-off	PPA	NPA
**CSF Lumipulse measures**				
p-tau181 (pg/ml)	0.92 (0.89 to 0.95)	51.8	0.96	0.80
Aβ42/Aβ40	0.92 (0.89 to 0.94)	0.055	0.96	0.82
Total tau (pg/ml)	0.87 (0.83 to 0.91)	358	0.94	0.71
Aβ42 (pg/ml)	0.87 (0.83 to 0.91)	625	0.90	0.75
Aβ40 (pg/ml)	0.51 (0.43 to 0.60)	11072	0.62	0.49
**Phosphorylation occupancies by mass spectrometry**				
pT217/T217 (%)	0.96 (0.94 to 0.98)	7.65	0.96	0.88
pT205/T205 (%)	0.94 (0.91 to 0.97)	1.21	0.90	0.85
pT111/T111 (%)	0.92 (0.89 to 0.95)	6.15	0.96	0.83
pT181/T181 (%)	0.90 (0.87 to 0.94)	34.26	0.85	0.89
pT153/T153 (%)	0.90 (0.86 to 0.93)	0.11	0.88	0.82
pS208/S208 (%)	0.90 (0.87 to 0.94)	0.21	0.96	0.80
pT231/T231 (%)	0.89 (0.86 to 0.93)	17	0.94	0.83
pS202/S202 (%)	0.66 (0.59 to 0.74)	5.02	0.58	0.71
pS199/S199 (%)	0.65 (0.57 to 0.72)	0.64	0.81	0.47
pT175/T175 (%)	0.57 (0.49 to 0.65)	0.46	0.67	0.51
**Phosphorylated tau concentrations by mass spectrometry**				
p-tau205 (pg/ml)	0.96 (0.94 to 0.98)	38.11	0.92	0.90
p-tau217 (pg/ml)	0.95 (0.93 to 0.97)	149	0.94	0.87
p-tau208 (pg/ml)	0.92 (0.89 to 0.95)	7.28	0.90	0.87
p-tau231 (pg/ml)	0.91 (0.88 to 0.94)	53.5	0.92	0.83
p-tau153 (pg/ml)	0.90 (0.87 to 0.93)	2.53	0.88	0.83
p-tau181 (pg/ml)	0.87 (0.83 to 0.91)	837	0.90	0.75
p-tau199 (pg/ml)	0.83 (0.78 to 0.89)	23.0	0.77	0.80
p-tau202 (pg/ml)	0.81 (0.75 to 0.87)	170	0.67	0.83
p-tau175 (pg/ml)	0.70 (0.62 to 0.77)	12.4	0.69	0.68
**Non-phosphorylated tau concentrations by mass spectrometry**				
Tau195-210 (ng/ml)	0.85 (0.80 to 0.89)	2.75	0.88	0.70
Tau151-155 (ng/ml)	0.83 (0.78 to 0.88)	2.27	0.85	0.70
Tau181-190 (ng/ml)	0.81 (0.75 to 0.87)	2.74	0.75	0.74
Tau226-230 (ng/ml)	0.81 (0.76 to 0.86)	0.29	0.79	0.76
Tau212-221 (ng/ml)	0.80 (0.74 to 0.85)	1.66	0.92	0.55

Supplemental Table 10. Correspondence of CSF measures with the tau PET summary measure for amyloid PET positive individuals in the Knight ADRC tau PET cohort. The receiver operating characteristic area under the curve of CSF measures with tau PET status is shown with 95% confidence intervals. The cut-off for the CSF measures that best distinguished tau PET status, as well as the positive percent agreement (PPA) and negative percent agreement (NPA) of the cut-off for tau PET status, are shown. CSF measures are listed in order of correspondence with tau PET status, stratified by measure type.

Analyte	AUC (95% CI)	Cut-off	PPA	NPA
**CSF Lumipulse measures**				
p-tau181 (pg/ml)	0.74 (0.66 to 0.83)	58.9	0.86	0.53
Aβ42/Aβ40	0.68 (0.59 to 0.77)	0.0477	0.78	0.54
Total tau (pg/ml)	0.69 (0.60 to 0.78)	589	0.49	0.82
Aβ42 (pg/ml)	0.68 (0.59 to 0.77)	430	0.41	0.86
Aβ40 (pg/ml)	0.59 (0.49 to 0.70)	11889	0.61	0.64
**Phosphorylation occupancies by mass spectrometry**				
pT205/T205 (%)	0.88 (0.82 to 0.94)	1.37	0.76	0.86
pT217/T217 (%)	0.83 (0.76 to 0.90)	10.4	0.80	0.80
pT111/T111 (%)	0.70 (0.61 to 0.79)	8.44	0.76	0.65
pS208/S208 (%)	0.69 (0.59 to 0.78)	0.25	0.82	0.53
pT181/T181 (%)	0.68 (0.58 to 0.77)	34.7	0.84	0.61
pT153/T153 (%)	0.67 (0.57 to 0.76)	0.139	0.73	0.64
pT231/T231 (%)	0.66 (0.55 to 0.75)	17.0	0.94	0.43
pS199/S199 (%)	0.59 (0.49 to 0.70)	0.85	0.45	0.74
pT175/T175 (%)	0.58 (0.49 to 0.69)	0.46	0.69	0.51
pS202/S202 (%)	0.55 (0.45 to 0.65)	5.00	0.59	0.61
**Phosphorylated tau concentrations by mass spectrometry**				
p-tau205 (pg/ml)	0.87 (0.81 to 0.93)	41.0	0.86	0.76
p-tau217 (pg/ml)	0.80 (0.72 to 0.88)	205	0.80	0.72
p-tau208 (pg/ml)	0.75 (0.66 to 0.84)	8.09	0.82	0.65
p-tau231 (pg/ml)	0.70 (0.61 to 0.80)	74.7	0.77	0.61
p-tau181 (pg/ml)	0.69 (0.60 to 0.78)	1142	0.65	0.69
p-tau202 (pg/ml)	0.69 (0.60 to 0.78)	171	0.67	0.68
p-tau153 (pg/ml)	0.69 (0.60 to 0.78)	3.71	0.78	0.63
p-tau199 (pg/ml)	0.69 (0.59 to 0.78)	23.1	0.76	0.59
p-tau175 (pg/ml)	0.55 (0.45 to 0.65)	12.3	0.71	0.42
**Non-phosphorylated tau concentrations by mass spectrometry**				
Tau195-210 (ng/ml)	0.69 (0.59 to 0.78)	2.67	0.90	0.41
Tau181-190 (ng/ml)	0.65 (0.55 to 0.75)	3.22	0.59	0.65
Tau151-155 (ng/ml)	0.65 (0.55 to 0.74)	2.44	0.76	0.48
Tau226-230 (ng/ml)	0.65 (0.55 to 0.75)	0.30	0.76	0.54
Tau212-221 (ng/ml)	0.64 (0.55 to 0.74)	1.66	0.92	0.31

Supplemental Table 11. Identification of individuals in the Knight ADRC tau PET cohort who are both tau PET positive and amyloid PET positive. The receiver operating characteristic area under the curve for individuals who are either both tau PET positive and amyloid PET positive, or any other status, is shown with 95% confidence intervals. The cut-off for the CSF measures that best distinguished individuals who were both tau PET positive and amyloid PET positive status, versus individuals in all other categories, as well as the positive percent agreement (PPA) and negative percent agreement (NPA) of the cut-off, are shown. CSF measures are listed in order of the best prediction of individuals who are both tau PET positive and amyloid PET positive, stratified by measure type.

Analyte	AUC (95% CI)	Cut-off	PPA	NPA
**CSF Lumipulse measures**				
p-tau181 (pg/ml)	0.92 (0.89 to 0.95)	51.8	0.96	0.79
Aβ42/Aβ40	0.91 (0.88 to 0.94)	0.0549	0.96	0.82
Aβ42 (pg/ml)	0.87 (0.82 to 0.91)	625	0.90	0.75
Total tau (pg/ml)	0.86 (0.83 to 0.91)	358	0.94	0.70
Aβ40 (pg/ml)	0.51 (0.43 to 0.60)	11072	0.61	0.48
**Phosphorylation occupancies by mass spectrometry**				
pT217/T217 (%)	0.96 (0.94 to 0.98)	7.65	0.96	0.88
pT205/T205 (%)	0.94 (0.91 to 0.97)	1.21	0.90	0.84
pT111/T111 (%)	0.92 (0.89 to 0.95)	6.15	0.96	0.83
pS208/S208 (%)	0.90 (0.86 to 0.94)	0.21	0.98	0.79
pT181/T181 (%)	0.90 (0.86 to 0.94)	34.3	0.84	0.89
pT153/T153 (%)	0.90 (0.86 to 0.93)	0.107	0.88	0.81
pT231/T231 (%)	0.89 (0.86 to 0.93)	17.0	0.94	0.82
pS202/S202 (%)	0.66 (0.59 to 0.74)	5.00	0.59	0.72
pS199/S199 (%)	0.64 (0.56 to 0.72)	0.639	0.80	0.47
pT175/T175 (%)	0.57 (0.50 to 0.65)	0.459	0.69	0.52
**Phosphorylated tau concentrations by mass spectrometry**				
p-tau205 (pg/ml)	0.96 (0.94 to 0.98)	38.1	0.92	0.90
p-tau217 (pg/ml)	0.95 (0.93 to 0.97)	149	0.94	0.87
p-tau208 (pg/ml)	0.92 (0.89 to 0.95)	7.27	0.90	0.86
p-tau231 (pg/ml)	0.91 (0.87 to 0.94)	53.5	0.92	0.83
p-tau153 (pg/ml)	0.90 (0.86 to 0.93)	2.53	0.88	0.83
p-tau181 (pg/ml)	0.87 (0.82 to 0.91)	837	0.90	0.75
p-tau199 (pg/ml)	0.83 (0.77 to 0.89)	23.0	0.76	0.79
p-tau202 (pg/ml)	0.80 (0.74 to 0.86)	170	0.67	0.82
p-tau175 (pg/ml)	0.69 (0.62 to 0.76)	12.38	0.69	0.68
**Non-phosphorylated tau concentrations by mass spectrometry**				
Tau195-210 (ng/ml)	0.84 (0.80 to 0.89)	2.75	0.88	0.69
Tau151-155 (ng/ml)	0.83 (0.77 to 0.88)	2.27	0.84	0.70
Tau226-230 (ng/ml)	0.81 (0.75 to 0.86)	0.289	0.78	0.76
Tau181-190 (ng/ml)	0.80 (0.75 to 0.86)	2.74	0.75	0.73
Tau212-221 (ng/ml)	0.79 (0.74 to 0.85)	1.66	0.92	0.55

Supplemental Table 12. Correlations between CSF biomarkers and the tau PET summary measure in the Knight ADRC tau PET cohort. The Spearman correlation and partial Spearman correlation after adjusting for age and sex are shown. CSF measures are shown in order of the absolute value of their correlation with the tau PET summary measure, stratified by measure type. All tests were two-sided and were not adjusted for multiple comparisons.

Measure	n=	Spearman ρ	p=	Partial Spearman ρ	p=
**CSF Lumipulse measures**					
p-tau181 (pg/ml)	371	0.45 (0.36 to 0.53)	<0.0001	0.37 (0.28 to 0.46)	<0.0001
Aβ42/Aβ40	371	-0.44 (-0.52 to -0.35)	<0.0001	-0.37 (-0.45 to -0.27)	<0.0001
Total tau (pg/ml)	371	0.41 (0.32 to 0.49)	<0.0001	0.34 (0.24 to 0.43)	<0.0001
Aβ42 (pg/ml)	371	-0.30 (-0.39 to -0.21)	<0.0001	-0.29 (-0.38 to -0.19)	<0.0001
Aβ40 (pg/ml)	371	0.13 (0.02 to 0.22)	0.02	0.04 (-0.06 to 0.14)	0.46
**Phosphorylation occupancies by mass spectrometry**					
pS208/S208 (%)	332	0.49 (0.40 to 0.57)	<0.0001	0.47 (0.38 to 0.55)	<0.0001
pT231/T231 (%)	298	0.49 (0.40 to 0.57)	<0.0001	0.44 (0.34 to 0.53)	<0.0001
pT153/T153 (%)	326	0.48 (0.39 to 0.56)	<0.0001	0.42 (0.33 to 0.51)	<0.0001
pT217/T217 (%)	371	0.47 (0.39 to 0.55)	<0.0001	0.44 (0.35 to 0.51)	<0.0001
pT205/T205 (%)	371	0.47 (0.39 to 0.55)	<0.0001	0.44 (0.36 to 0.52)	<0.0001
pT111/T111 (%)	369	0.41 (0.32 to 0.49)	<0.0001	0.39 (0.30 to 0.47)	<0.0001
pT181/T181 (%)	370	0.36 (0.26 to 0.44)	<0.0001	0.32 (0.22 to 0.41)	<0.0001
pS199/S199 (%)	368	0.17 (0.07 to 0.27)	0.0009	0.16 (0.06 to 0.26)	0.003
pS202/S202 (%)	371	-0.14 (-0.24 to -0.04)	0.006	-0.10 (-0.20 to 0.01)	0.06
pT175/T175 (%)	363	-0.04 (-0.14 to 0.06)	0.46	-0.05 (-0.16 to 0.05)	0.31
**Phosphorylated tau concentrations by mass spectrometry**					
p-tau208 (pg/ml)	332	0.52 (0.44 to 0.60)	<0.0001	0.48 (0.39 to 0.56)	<0.0001
p-tau205 (pg/ml)	371	0.51 (0.43 to 0.58)	<0.0001	0.45 (0.36 to 0.53)	<0.0001
p-tau231 (pg/ml)	297	0.50 (0.41 to 0.58)	<0.0001	0.45 (0.36 to 0.54)	<0.0001
p-tau217 (pg/ml)	371	0.49 (0.41 to 0.57)	<0.0001	0.43 (0.34 to 0.51)	<0.0001
p-tau153 (pg/ml)	326	0.49 (0.40 to 0.56)	<0.0001	0.42 (0.33 to 0.51)	<0.0001
p-tau181 (pg/ml)	370	0.39 (0.30 to 0.48)	<0.0001	0.31 (0.21 to 0.40)	<0.0001
p-tau202 (pg/ml)	371	0.37 (0.28 to 0.45)	<0.0001	0.28 (0.18 to 0.37)	<0.0001
p-tau199 (pg/ml)	368	0.36 (0.27 to 0.45)	<0.0001	0.29 (0.20 to 0.38)	<0.0001
p-tau175 (pg/ml)	363	0.25 (0.15 to 0.34)	<0.0001	0.17 (0.07 to 0.27)	0.001
**Non-phosphorylated tau concentrations by mass spectrometry**					
Tau151-155 (ng/ml)	369	0.48 (0.40 to 0.56)	<0.0001	0.27 (0.17 to 0.36)	<0.0001
Tau195-210 (ng/ml)	371	0.48 (0.40 to 0.56)	<0.0001	0.28 (0.19 to 0.37)	<0.0001
Tau181-190 (ng/ml)	371	0.42 (0.34 to 0.50)	<0.0001	0.25 (0.15 to 0.34)	<0.0001
Tau212-221 (ng/ml)	371	0.42 (0.33 to 0.50)	<0.0001	0.24 (0.14 to 0.33)	<0.0001
Tau226-230 (ng/ml)	351	0.30 (0.20 to 0.39)	<0.0001	0.24 (0.14 to 0.34)	<0.0001

Supplemental Table 13. Correlations between CSF biomarkers and the tau PET summary measure for individuals in the Knight ADRC tau PET cohort with no missing CSF biomarker measures (n=264). The Spearman correlation and partial Spearman correlation after adjusting for age and sex are shown. CSF measures are shown in order of the absolute value of their correlation with the tau PET summary measure, stratified by measure type. All tests were two-sided and were not adjusted for multiple comparisons.

Measure	Spearman ρ	p=	Partial Spearman ρ	p=
**CSF Lumipulse measures**				
Aβ42/Aβ40	-0.53 (-0.61 to -0.43)	<0.0001	-0.46 (-0.55 to -0.36)	<0.0001
p-tau181 (pg/ml)	0.52 (0.43 to 0.60)	<0.0001	0.46 (0.36 to 0.55)	<0.0001
Total tau (pg/ml)	0.45 (0.35 to 0.54)	<0.0001	0.39 (0.29 to 0.49)	<0.0001
Aβ42 (pg/ml)	-0.42 (-0.51 to -0.31)	<0.0001	-0.38 (-0.48 to -0.27)	<0.0001
Aβ40 (pg/ml)	0.08 (-0.04 to 0.20)	0.19	0.03 (-0.09 to 0.15)	0.62
**Phosphorylation occupancies by mass spectrometry**				
pT217/T217 (%)	0.59 (0.51 to 0.66)	<0.0001	0.56 (0.47 to 0.64)	<0.0001
pS208/S208 (%)	0.58 (0.50 to 0.66)	<0.0001	0.56 (0.47 to 0.64)	<0.0001
pT205/T205 (%)	0.52 (0.43 to 0.61)	<0.0001	0.51 (0.41 to 0.59)	<0.0001
pT111/T111 (%)	0.51 (0.42 to 0.60)	<0.0001	0.48 (0.38 to 0.57)	<0.0001
pT231/T231 (%)	0.51 (0.42 to 0.59)	<0.0001	0.47 (0.37 to 0.56)	<0.0001
pT153/T153 (%)	0.49 (0.39 to 0.57)	<0.0001	0.45 (0.35 to 0.54)	<0.0001
pT181/T181 (%)	0.42 (0.32 to 0.51)	<0.0001	0.39 (0.29 to 0.49)	<0.0001
pS202/S202 (%)	-0.18 (-0.30 to -0.07)	0.003	-0.15 (-0.27 to -0.03)	0.01
pS199/S199 (%)	0.17 (0.05 to 0.29)	0.005	0.17 (0.05 to 0.29)	0.005
pT175/T175 (%)	-0.13 (-0.24 to -0.01)	0.04	-0.10 (-0.22 to 0.02)	0.10
**Phosphorylated tau concentrations by mass spectrometry**				
p-tau208 (pg/ml)	0.60 (0.52 to 0.68)	<0.0001	0.57 (0.48 to 0.64)	<0.0001
p-tau205 (pg/ml)	0.59 (0.50 to 0.66)	<0.0001	0.54 (0.45 to 0.62)	<0.0001
p-tau217 (pg/ml)	0.59 (0.50 to 0.66)	<0.0001	0.54 (0.45 to 0.62)	<0.0001
p-tau231 (pg/ml)	0.53 (0.44 to 0.61)	<0.0001	0.48 (0.39 to 0.57)	<0.0001
p-tau153 (pg/ml)	0.50 (0.40 to 0.59)	<0.0001	0.46 (0.35 to 0.55)	<0.0001
p-tau181 (pg/ml)	0.45 (0.34 to 0.54)	<0.0001	0.38 (0.27 to 0.48)	<0.0001
p-tau199 (pg/ml)	0.41 (0.31 to 0.51)	<0.0001	0.37 (0.26 to 0.47)	<0.0001
p-tau202 (pg/ml)	0.38 (0.27 to 0.48)	<0.0001	0.31 (0.19 to 0.41)	<0.0001
p-tau175 (pg/ml)	0.24 (0.13 to 0.35)	<0.0001	0.19 (0.07 to 0.31)	0.0016
**Non-phosphorylated tau concentrations by mass spectrometry**				
Tau195-210 (ng/ml)	0.42 (0.32 to 0.52)	<0.0001	0.35 (0.24 to 0.45)	<0.0001
Tau151-155 (ng/ml)	0.40 (0.30 to 0.50)	<0.0001	0.33 (0.22 to 0.44)	<0.0001
Tau226-230 (ng/ml)	0.40 (0.29 to 0.50)	<0.0001	0.34 (0.23 to 0.44)	<0.0001
Tau181-190 (ng/ml)	0.38 (0.27 to 0.48)	<0.0001	0.31 (0.19 to 0.41)	<0.0001
Tau212-221 (ng/ml)	0.37 (0.26 to 0.47)	<0.0001	0.29 (0.18 to 0.40)	<0.0001

Supplemental Table 14. Correlations between CSF biomarkers and the tau PET summary measure for amyloid PET positive individuals in the Knight ADRC tau PET cohort. The Spearman correlation and partial Spearman correlation after adjusting for age and sex are shown. CSF measures are shown in order of the absolute value of their correlation with the tau PET summary measure, stratified by measure type. All tests were two-sided and were not adjusted for multiple comparisons.

Measure	n=	Spearman ρ	p=	Partial Spearman ρ	p=
**CSF Lumipulse measures**					
Aβ42 (pg/ml)	125	-0.37 (-0.51 to -0.20)	<0.0001	-0.37 (-0.51 to -0.21)	<0.0001
p-tau181 (pg/ml)	125	0.36 (0.20 to 0.51)	<0.0001	0.32 (0.15 to 0.47)	0.0003
Aβ42/Aβ40	125	-0.31 (-0.46 to -0.15)	0.0003	-0.28 (-0.43 to -0.11)	0.002
Total tau (pg/ml)	125	0.29 (0.12 to 0.44)	0.001	0.25 (0.08 to 0.41)	0.004
Aβ40 (pg/ml)	125	-0.21 (-0.37 to -0.04)	0.02	-0.25 (-0.41 to -0.07)	0.005
**Phosphorylation occupancies by mass spectrometry**					
pT205/T205 (%)	125	0.72 (0.63 to 0.80)	<0.0001	0.72 (0.62 to 0.80)	<0.0001
pT217/T217 (%)	125	0.55 (0.42 to 0.67)	<0.0001	0.54 (0.41 to 0.66)	<0.0001
pS208/S208 (%)	123	0.35 (0.18 to 0.49)	<0.0001	0.33 (0.17 to 0.48)	0.0001
pT111/T111 (%)	125	0.30 (0.13 to 0.45)	0.0006	0.27 (0.10 to 0.43)	0.002
pT181/T181 (%)	125	0.28 (0.11 to 0.44)	0.001	0.26 (0.08 to 0.41)	0.004
pT153/T153 (%)	124	0.28 (0.11 to 0.43)	0.002	0.26 (0.09 to 0.42)	0.004
pT231/T231 (%)	115	0.25 (0.07 to 0.41)	0.007	0.23 (0.04 to 0.40)	0.01
pS199/S199 (%)	125	0.19 (0.02 to 0.36)	0.03	0.19 (0.01 to 0.35)	0.04
pT175/T175 (%)	123	-0.09 (-0.27 to 0.09)	0.30	-0.13 (-0.30 to 0.05)	0.15
pS202/S202 (%)	125	-0.04 (-0.21 to 0.14)	0.65	-0.03 (-0.21 to 0.15)	0.75
**Phosphorylated tau concentrations by mass spectrometry**					
p-tau205 (pg/ml)	125	0.65 (0.54 to 0.74)	<0.0001	0.63 (0.51 to 0.73)	<0.0001
p-tau217 (pg/ml)	125	0.48 (0.33 to 0.60)	<0.0001	0.45 (0.29 to 0.58)	<0.0001
p-tau208 (pg/ml)	123	0.41 (0.25 to 0.55)	<0.0001	0.38 (0.21 to 0.52)	<0.0001
p-tau231 (pg/ml)	114	0.33 (0.16 to 0.49)	0.0003	0.31 (0.13 to 0.47)	0.0009
p-tau153 (pg/ml)	124	0.31 (0.14 to 0.46)	0.0004	0.28 (0.10 to 0.43)	0.002
p-tau202 (pg/ml)	125	0.31 (0.14 to 0.46)	0.0003	0.26 (0.09 to 0.42)	0.003
p-tau199 (pg/ml)	125	0.30 (0.13 to 0.45)	0.0006	0.26 (0.09 to 0.42)	0.003
p-tau181 (pg/ml)	125	0.28 (0.11 to 0.44)	0.001	0.23 (0.06 to 0.39)	0.009
p-tau175 (pg/ml)	123	0.10 (-0.08 to 0.27)	0.28	0.05 (-0.13 to 0.22)	0.61
**Non-phosphorylated tau concentrations by mass spectrometry**					
Tau195-210 (ng/ml)	125	0.27 (0.10 to 0.43)	0.002	0.22 (0.05 to 0.38)	0.01
Tau181-190 (ng/ml)	125	0.22 (0.04 to 0.38)	0.01	0.16 (-0.02 to 0.33)	0.07
Tau226-230 (ng/ml)	122	0.20 (0.02 to 0.37)	0.03	0.18 0.00 to 0.35)	0.05
Tau151-155 (ng/ml)	124	0.20 (0.02 to 0.36)	0.03	0.14 (-0.03 to 0.31)	0.11
Tau212-221 (ng/ml)	125	0.20 (0.02 to 0.36)	0.03	0.14 (-0.03 to 0.31)	0.11

Supplemental Table 15. Correlations between CSF pT205/T205 and regional tau PET for amyloid PET positive individuals in the Knight ADRC tau PET cohort. The partial Spearman correlation after adjusting for age and sex are shown. All tests were two-sided and the significance of correlations was adjusted for comparisons with multiple regions using the Benjamini-Hochberg procedure.

Region of interest	Freesurfer label	Group	Partial Spearman ρ	Adjusted p-value
inferior temporal gyrus	INFRTMP	Temporal	0.68 (0.59 to 0.75)	<0.0001
fusiform gyrus	FUSIFORM	Temporal	0.65 (0.56 to 0.73)	<0.0001
amygdala	AMYGDALA	Subcortical	0.64 (0.55 to 0.71)	<0.0001
parahippocampal gyrus	PARAHPCMPL	Temporal	0.62 (0.52 to 0.70)	<0.0001
entorhinal	ENTORHINAL	Temporal	0.61 (0.51 to 0.69)	<0.0001
middle temporal gyrus	MIDTMP	Temporal	0.61 (0.52 to 0.70)	<0.0001
inferior parietal cortex	INFRPRTL	Parietal	0.60 (0.50 to 0.68)	<0.0001
banks of the superior temporal sulcus	SSTSBANK	Temporal	0.53 (0.43 to 0.63)	<0.0001
supramarginal gyrus	SUPRAMRGNL	Parietal	0.52 (0.41 to 0.61)	<0.0001
superior temporal cortex	SUPERTMP	Temporal	0.50 (0.38 to 0.60)	<0.0001
isthmus of the cingulate gyrus	ISTHMUSCNG	Parietal	0.49 (0.38 to 0.59)	<0.0001
temporal pole	TMPPOLE	Temporal	0.49 (0.37 to 0.59)	<0.0001
precuneus	PRECUNEUS	Parietal	0.47 (0.35 to 0.57)	<0.0001
hippcampus	HIPPOCAMPUS	Subcortical	0.47 (0.36 to 0.57)	<0.0001
caudal middle frontal gyrus	CAUDMIDFRN	Frontal	0.44 (0.32 to 0.55)	<0.0001
superior parietal cortex	SUPERPRTL	Parietal	0.44 (0.32 to 0.54)	<0.0001
lateral occipital cortex	LATOCC	Occipital	0.37 (0.25 to 0.49)	0.0001
postcentral gyrus	POSTCNTRL	Parietal	0.36 (0.23 to 0.48)	0.0001
insula	INSULA	Insula	0.35 (0.22 to 0.47)	0.0001
pars orbitalis	PARSORBLS	Frontal	0.33 (0.20 to 0.45)	0.0004
lateral orbitofrontal cortex	LATORBFRN	Frontal	0.32 (0.18 to 0.44)	0.0007
precentral gyrus	PRECNTRL	Frontal	0.31 (0.18 to 0.43)	0.0008
superior frontal cortex	SUPERFRN	Frontal	0.31 (0.17 to 0.43)	0.001
rostral middle frontal cortex	ROSMIDFRN	Frontal	0.27 (0.13 to 0.39)	0.005
putamen	PUTAMEN	Subcortical	0.27 (0.13 to 0.39)	0.005
pars opercularus	PARAOPRCLRS	Frontal	0.26 (0.13 to 0.39)	0.005
pars triangularus	PARSTRNGLRS	Frontal	0.25 (0.11 to 0.37)	0.009
cuneus	CUNEUS	Occipital	0.22 (0.08 to 0.35)	0.02
lingual gyrus	LINGUAL	Occipital	0.19 (0.05 to 0.32)	0.05
medial orbitalfrontal cortex	MEDORBFRN	Frontal	0.19 (0.05 to 0.32)	0.05
posterior cingulate gyrus	POSTCNG	Parietal	0.19 (0.05 to 0.32)	0.05
caudate	CAUD	Subcortical	0.18 (0.04 to 0.31)	0.06
thalamus	THALAMUS	Subcortical	0.18 (0.04 to 0.31)	0.06
pallidum	PALLIDUM	Subcortical	0.17 (0.03 to 0.31)	0.07
ventral diencephalon	VENTRALDC	Subcortical	0.09 (-0.05 to 0.23)	0.38
transverse temporal gyrus	TRANSTMP	Temporal	-0.07 (-0.21 to 0.07)	0.51
paracentral gyrus	PARACNTRL	Frontal	0.06 (-0.08 to 0.20)	0.55
rostral anterior cingulate	ROSANTCNG	Frontal	0.04 (-0.10 to 0.18)	0.69
frontal pole	FRNPOLE	Frontal	0.01 (-0.13 to 0.15)	0.92
caudal anterior cingulate gyrus	CAUDANTCNG	Frontal	0.00 (-0.14 to 0.14)	0.98
pericalcarine cortex	PERICLCRN	Occipital	0.00 (-0.14 to 0.14)	1.00

Supplemental Table 16. Correlations between CSF pT217/T217 and regional tau PET for amyloid PET positive individuals in the Knight ADRC tau PET cohort. The partial Spearman correlation after adjusting for age and sex are shown. All tests were two-sided and the significance of correlations was adjusted for comparisons with multiple regions using the Benjamini-Hochberg procedure.

Region of interest	Freesurfer label	Group	Partial Spearman ρ	Adjusted p-value
inferior temporal gyrus	INFRTMP	Temporal	0.55 (0.44 to 0.64)	<0.0001
parahippocampal gyrus	PARAHPCMPL	Temporal	0.54 (0.43 to 0.63)	<0.0001
middle temporal gyrus	MIDTMP	Temporal	0.53 (0.42 to 0.62)	<0.0001
fusiform gyrus	FUSIFORM	Temporal	0.50 (0.39 to 0.60)	<0.0001
amygdala	AMYGDALA	Subcortical	0.49 (0.38 to 0.59)	<0.0001
isthmus of the cingulate gyrus	ISTHMUSCNG	Parietal	0.49 (0.37 to 0.59)	<0.0001
inferior parietal cortex	INFRPRTL	Parietal	0.48 (0.37 to 0.58)	<0.0001
entorhinal	ENTORHINAL	Temporal	0.48 (0.36 to 0.58)	<0.0001
banks of the superior temporal sulcus	SSTSBANK	Temporal	0.45 (0.33 to 0.55)	<0.0001
supramarginal gyrus	SUPRAMRGNL	Parietal	0.43 (0.30 to 0.53)	<0.0001
superior temporal cortex	SUPERTMP	Temporal	0.42 (0.30 to 0.53)	<0.0001
caudal middle frontal gyrus	CAUDMIDFRN	Frontal	0.38 (0.25 to 0.49)	0.0001
precuneus	PRECUNEUS	Parietal	0.37 (0.24 to 0.49)	0.0001
temporal pole	TMPPOLE	Temporal	0.33 (0.20 to 0.45)	0.0005
postcentral gyrus	POSTCNTRL	Parietal	0.31 (0.18 to 0.43)	0.001
hippcampus	HIPPOCAMPUS	Subcortical	0.30 (0.17 to 0.43)	0.002
superior parietal cortex	SUPERPRTL	Parietal	0.30 (0.16 to 0.42)	0.002
lateral occipital cortex	LATOCC	Occipital	0.27 (0.13 to 0.39)	0.006
insula	INSULA	Insula	0.27 (0.13 to 0.39)	0.006
rostral middle frontal cortex	ROSMIDFRN	Frontal	0.25 (0.11 to 0.37)	0.01
lateral orbitofrontal cortex	LATORBFRN	Frontal	0.23 (0.09 to 0.36)	0.02
superior frontal cortex	SUPERFRN	Frontal	0.21 (0.07 to 0.34)	0.03
pars opercularus	PARAOPRCLRS	Frontal	0.19 (0.05 to 0.32)	0.06
precentral gyrus	PRECNTRL	Frontal	0.17 (0.03 to 0.30)	0.10
medial orbitalfrontal cortex	MEDORBFRN	Frontal	0.17 (0.03 to 0.30)	0.10
pars triangularus	PARSTRNGLRS	Frontal	0.16 (0.03 to 0.30)	0.10
pars orbitalis	PARSORBLS	Frontal	0.16 (0.02 to 0.30)	0.10
cuneus	CUNEUS	Occipital	0.16 (0.02 to 0.29)	0.11
pericalcarine cortex	PERICLCRN	Occipital	-0.15 (-0.28 to -0.01)	0.14
ventral diencephalon	VENTRALDC	Subcortical	-0.11 (-0.25 to 0.03)	0.28
thalamus	THALAMUS	Subcortical	-0.11 (-0.24 to 0.03)	0.32
paracentral gyrus	PARACNTRL	Frontal	-0.07 (-0.21 to 0.07)	0.55
lingual gyrus	LINGUAL	Occipital	0.06 (-0.08 to 0.20)	0.62
rostral anterior cingulate	ROSANTCNG	Frontal	0.06 (-0.09 to 0.19)	0.63
caudal anterior cingulate gyrus	CAUDANTCNG	Frontal	0.05 (-0.09 to 0.19)	0.63
posterior cingulate gyrus	POSTCNG	Parietal	0.05 (-0.09 to 0.19)	0.63
transverse temporal gyrus	TRANSTMP	Temporal	-0.04 (-0.18 to 0.10)	0.71
pallidum	PALLIDUM	Subcortical	-0.02 (-0.16 to 0.12)	0.86
frontal pole	FRNPOLE	Frontal	0.02 (-0.12 to 0.16)	0.86
caudate	CAUD	Subcortical	0.02 (-0.12 to 0.16)	0.86
putamen	PUTAMEN	Subcortical	0.00 (-0.14 to 0.14)	0.99

Supplemental Table 17. Correlations between CSF pT181/T181 and regional tau PET for amyloid PET positive individuals in the Knight ADRC tau PET cohort. The partial Spearman correlation after adjusting for age and sex are shown. All tests were two-sided and the significance of correlations was adjusted for comparisons with multiple regions using the Benjamini-Hochberg procedure.

Region of interest	Freesurfer label	Group	Partial Spearman ρ	Adjusted p-value
parahippocampal gyrus	PARAHPCMPL	Temporal	0.31 (0.18 to 0.43)	0.02
isthmus of the cingulate gyrus	ISTHMUSCNG	Parietal	0.29 (0.16 to 0.42)	0.02
inferior temporal gyrus	INFRTMP	Temporal	0.27 (0.14 to 0.40)	0.03
entorhinal	ENTORHINAL	Temporal	0.27 (0.13 to 0.39)	0.03
fusiform gyrus	FUSIFORM	Temporal	0.25 (0.11 to 0.38)	0.04
amygdala	AMYGDALA	Subcortical	0.23 (0.09 to 0.36)	0.07
middle temporal gyrus	MIDTMP	Temporal	0.22 (0.08 to 0.35)	0.09
banks of the superior temporal sulcus	SSTSBANK	Temporal	0.21 (0.08 to 0.34)	0.09
inferior parietal cortex	INFRPRTL	Parietal	0.19 (0.05 to 0.32)	0.16
superior temporal cortex	SUPERTMP	Temporal	0.18 (0.04 to 0.31)	0.20
caudal middle frontal gyrus	CAUDMIDFRN	Frontal	0.17 (0.03 to 0.31)	0.20
ventral diencephalon	VENTRALDC	Subcortical	-0.17 (-0.30 to -0.03)	0.20
pericalcarine cortex	PERICLCRN	Occipital	-0.17 (-0.30 to -0.03)	0.20
supramarginal gyrus	SUPRAMRGNL	Parietal	0.16 (0.02 to 0.29)	0.23
precuneus	PRECUNEUS	Parietal	0.15 (0.01 to 0.28)	0.30
temporal pole	TMPPOLE	Temporal	0.13 (-0.01 to 0.27)	0.37
hippcampus	HIPPOCAMPUS	Subcortical	0.12 (-0.02 to 0.25)	0.46
paracentral gyrus	PARACNTRL	Frontal	-0.09 (-0.23 to 0.05)	0.68
postcentral gyrus	POSTCNTRL	Parietal	0.09 (-0.05 to 0.23)	0.68
pallidum	PALLIDUM	Subcortical	-0.09 (-0.23 to 0.05)	0.68
lateral occipital cortex	LATOCC	Occipital	0.08 (-0.06 to 0.22)	0.68
thalamus	THALAMUS	Subcortical	-0.08 (-0.22 to 0.06)	0.68
putamen	PUTAMEN	Subcortical	-0.08 (-0.22 to 0.06)	0.68
rostral middle frontal cortex	ROSMIDFRN	Frontal	0.07 (-0.07 to 0.20)	0.79
insula	INSULA	Insula	0.06 (-0.08 to 0.20)	0.81
lingual gyrus	LINGUAL	Occipital	-0.05 (-0.18 to 0.10)	0.90
superior frontal cortex	SUPERFRN	Frontal	0.04 (-0.10 to 0.18)	0.90
cuneus	CUNEUS	Occipital	-0.04 (-0.18 to 0.10)	0.90
pars opercularus	PARAOPRCLRS	Frontal	0.04 (-0.10 to 0.18)	0.90
pars orbitalis	PARSORBLS	Frontal	0.04 (-0.10 to 0.18)	0.90
transverse temporal gyrus	TRANSTMP	Temporal	-0.04 (-0.18 to 0.10)	0.90
lateral orbitofrontal cortex	LATORBFRN	Frontal	0.04 (-0.11 to 0.17)	0.90
pars triangularus	PARSTRNGLRS	Frontal	0.03 (-0.11 to 0.17)	0.91
rostral anterior cingulate	ROSANTCNG	Frontal	0.03 (-0.11 to 0.17)	0.91
frontal pole	FRNPOLE	Frontal	-0.02 (-0.16 to 0.12)	0.91
superior parietal cortex	SUPERPRTL	Parietal	0.02 (-0.12 to 0.16)	0.91
posterior cingulate gyrus	POSTCNG	Parietal	0.02 (-0.12 to 0.16)	0.91
caudal anterior cingulate gyrus	CAUDANTCNG	Frontal	-0.01 (-0.15 to 0.13)	0.96
caudate	CAUD	Subcortical	-0.01 (-0.15 to 0.13)	0.98
precentral gyrus	PRECNTRL	Frontal	0.00 (-0.14 to 0.14)	1.00
medial orbitalfrontal cortex	MEDORBFRN	Frontal	0.00 (-0.14 to 0.14)	1.00

Supplemental Table 18. Correlations between CSF pT231/T231 and regional tau PET for amyloid PET positive individuals in the Knight ADRC tau PET cohort. The partial Spearman correlation after adjusting for age and sex are shown. All tests were two-sided and the significance of correlations was adjusted for comparisons with multiple regions using the Benjamini-Hochberg procedure.

Region of interest	Freesurfer label	Group	Partial Spearman ρ	Adjusted p-value
inferior temporal gyrus	INFRTMP	Temporal	0.31 (0.17 to 0.44)	0.03
fusiform gyrus	FUSIFORM	Temporal	0.27 (0.13 to 0.40)	0.08
banks of the superior temporal sulcus	SSTSBANK	Temporal	0.25 (0.11 to 0.39)	0.08
inferior parietal cortex	INFRPRTL	Parietal	0.25 (0.10 to 0.38)	0.08
isthmus of the cingulate gyrus	ISTHMUSCNG	Parietal	0.24 (0.10 to 0.37)	0.08
parahippocampal gyrus	PARAHPCMPL	Temporal	0.24 (0.09 to 0.37)	0.08
middle temporal gyrus	MIDTMP	Temporal	0.23 (0.09 to 0.36)	0.09
supramarginal gyrus	SUPRAMRGNL	Parietal	0.21 (0.06 to 0.34)	0.15
superior temporal cortex	SUPERTMP	Temporal	0.20 (0.05 to 0.34)	0.16
precuneus	PRECUNEUS	Parietal	0.19 (0.05 to 0.33)	0.16
entorhinal	ENTORHINAL	Temporal	0.18 (0.03 to 0.32)	0.22
amygdala	AMYGDALA	Subcortical	0.17 (0.02 to 0.31)	0.25
pericalcarine cortex	PERICLCRN	Occipital	-0.17 (-0.31 to -0.02)	0.25
lateral occipital cortex	LATOCC	Occipital	0.16 (0.01 to 0.30)	0.28
caudal middle frontal gyrus	CAUDMIDFRN	Frontal	0.14 (0.00 to 0.28)	0.35
insula	INSULA	Insula	0.14 (-0.01 to 0.28)	0.36
rostral middle frontal cortex	ROSMIDFRN	Frontal	0.14 (-0.01 to 0.28)	0.36
pars opercularus	PARAOPRCLRS	Frontal	0.13 (-0.02 to 0.27)	0.40
postcentral gyrus	POSTCNTRL	Parietal	0.12 (-0.02 to 0.26)	0.42
superior parietal cortex	SUPERPRTL	Parietal	0.12 (-0.03 to 0.26)	0.42
paracentral gyrus	PARACNTRL	Frontal	-0.11 (-0.26 to 0.03)	0.45
lateral orbitofrontal cortex	LATORBFRN	Frontal	0.10 (-0.05 to 0.24)	0.53
medial orbitalfrontal cortex	MEDORBFRN	Frontal	0.10 (-0.05 to 0.24)	0.53
thalamus	THALAMUS	Subcortical	-0.09 (-0.23 to 0.06)	0.61
pars orbitalis	PARSORBLS	Frontal	0.08 (-0.07 to 0.23)	0.62
transverse temporal gyrus	TRANSTMP	Temporal	0.08 (-0.07 to 0.23)	0.62
putamen	PUTAMEN	Subcortical	-0.07 (-0.21 to 0.08)	0.73
cuneus	CUNEUS	Occipital	0.06 (-0.08 to 0.21)	0.74
temporal pole	TMPPOLE	Temporal	0.06 (-0.09 to 0.20)	0.75
hippcampus	HIPPOCAMPUS	Subcortical	0.06 (-0.09 to 0.20)	0.74
frontal pole	FRNPOLE	Frontal	0.05 (-0.09 to 0.20)	0.75
pars triangularus	PARSTRNGLRS	Frontal	0.05 (-0.10 to 0.20)	0.76
rostral anterior cingulate	ROSANTCNG	Frontal	0.04 (-0.11 to 0.18)	0.83
caudate	CAUD	Subcortical	0.04 (-0.11 to 0.18)	0.83
caudal anterior cingulate gyrus	CAUDANTCNG	Frontal	0.03 (-0.12 to 0.18)	0.86
superior frontal cortex	SUPERFRN	Frontal	0.03 (-0.12 to 0.17)	0.86
posterior cingulate gyrus	POSTCNG	Parietal	-0.03 (-0.17 to 0.12)	0.86
precentral gyrus	PRECNTRL	Frontal	0.02 (-0.13 to 0.17)	0.86
pallidum	PALLIDUM	Subcortical	0.02 (-0.13 to 0.16)	0.89
ventral diencephalon	VENTRALDC	Subcortical	-0.02 (-0.17 to 0.13)	0.86
lingual gyrus	LINGUAL	Occipital	0.00 (-0.15 to 0.15)	1.00

Supplemental Table 19. Correlations between CSF pT205/T205 and regional brain volumes for amyloid PET positive individuals in the Knight ADRC amyloid PET cohort. The partial Spearman correlation after adjusting for age and sex are shown. All tests were two-sided and the significance of correlations was adjusted for comparisons with multiple regions using the Benjamini-Hochberg procedure.

Region of interest	Freesurfer label	Group	Partial Spearman ρ	Adjusted p-value
hippcampus	HIPPOCAMPUS	Subcortical	-0.50 (-0.57 to -0.43)	<0.0001
amygdala	AMYGDALA	Subcortical	-0.45 (-0.53 to -0.37)	<0.0001
temporal pole	TMPPOLE	Temporal	-0.40 (-0.47 to -0.31)	<0.0001
middle temporal gyrus	MIDTMP	Temporal	-0.39 (-0.47 to -0.30)	<0.0001
fusiform gyrus	FUSIFORM	Temporal	-0.38 (-0.45 to -0.29)	<0.0001
entorhinal	ENTORHINAL	Temporal	-0.37 (-0.45 to -0.28)	<0.0001
inferior temporal gyrus	INFRTMP	Temporal	-0.35 (-0.43 to -0.26)	<0.0001
parahippocampal gyrus	PARAHPCMPL	Temporal	-0.33 (-0.41 to -0.24)	<0.0001
supramarginal gyrus	SUPRAMRGNL	Parietal	-0.32 (-0.40 to -0.23)	<0.0001
inferior parietal cortex	INFRPRTL	Parietal	-0.31 (-0.39 to -0.22)	<0.0001
precuneus	PRECUNEUS	Parietal	-0.28 (-0.37 to -0.19)	<0.0001
lateral orbitofrontal cortex	LATORBFRN	Frontal	-0.27 (-0.36 to -0.18)	<0.0001
isthmus of the cingulate gyrus	ISTHMUSCNG	Parietal	-0.24 (-0.33 to -0.15)	0.0003
insula	INSULA	Insula	-0.24 (-0.33 to -0.14)	0.0003
lingual gyrus	LINGUAL	Occipital	-0.23 (-0.32 to -0.14)	0.0005
rostral middle frontal cortex	ROSMIDFRN	Frontal	-0.23 (-0.32 to -0.14)	0.0005
posterior cingulate gyrus	POSTCNG	Parietal	-0.22 (-0.31 to -0.13)	0.0007
pars orbitalis	PARSORBLS	Frontal	-0.22 (-0.31 to -0.12)	0.0009
superior parietal cortex	SUPERPRTL	Parietal	-0.21 (-0.30 to -0.12)	0.001
rostral anterior cingulate	ROSANTCNG	Frontal	-0.21 (-0.30 to -0.12)	0.001
banks of the superior temporal sulcus	SSTSBANK	Temporal	-0.21 (-0.30 to -0.12)	0.001
superior temporal cortex	SUPERTMP	Temporal	-0.20 (-0.29 to -0.11)	0.002
caudal middle frontal gyrus	CAUDMIDFRN	Frontal	-0.20 (-0.29 to -0.11)	0.002
putamen	PUTAMEN	Subcortical	-0.19 (-0.28 to -0.10)	0.004
lateral occipital cortex	LATOCC	Occipital	-0.17 (-0.26 to -0.08)	0.008
pars opercularus	PARAOPRCLRS	Frontal	-0.16 (-0.26 to -0.07)	0.01
superior frontal cortex	SUPERFRN	Frontal	-0.16 (-0.25 to -0.06)	0.02
thalamus	THALAMUS	Subcortical	-0.15 (-0.25 to -0.06)	0.02
postcentral gyrus	POSTCNTRL	Parietal	-0.15 (-0.24 to -0.05)	0.02
pars triangularus	PARSTRNGLRS	Frontal	-0.15 (-0.24 to -0.05)	0.02
caudal anterior cingulate gyrus	CAUDANTCNG	Frontal	-0.13 (-0.23 to -0.04)	0.04
medial orbitalfrontal cortex	MEDORBFRN	Frontal	-0.13 (-0.22 to -0.03)	0.05
cuneus	CUNEUS	Occipital	-0.12 (-0.21 to -0.02)	0.07
frontal pole	FRNPOLE	Frontal	-0.11 (-0.21 to -0.02)	0.09
ventral diencephalon	VENTRALDC	Subcortical	-0.11 (-0.20 to -0.01)	0.10
pericalcarine cortex	PERICLCRN	Occipital	-0.09 (-0.18 to 0.01)	0.19
precentral gyrus	PRECNTRL	Frontal	-0.07 (-0.17 to 0.02)	0.26
pallidum	PALLIDUM	Subcortical	0.05 (-0.05 to 0.14)	0.46
transverse temporal gyrus	TRANSTMP	Temporal	-0.04 (-0.14 to 0.05)	0.49
caudate	CAUD	Subcortical	-0.03 (-0.12 to 0.07)	0.67
paracentral gyrus	PARACNTRL	Frontal	0.00 (-0.09 to 0.10)	0.95

Supplemental Table 20. Correlations between CSF pT217/T217 and regional brain volumes for amyloid PET positive individuals in the Knight ADRC amyloid PET cohort. The partial Spearman correlation after adjusting for age and sex are shown. All tests were two-sided and the significance of correlations was adjusted for comparisons with multiple regions using the Benjamini-Hochberg procedure.

Region of interest	Freesurfer label	Group	Partial Spearman ρ	Adjusted p-value
hippcampus	HIPPOCAMPUS	Subcortical	-0.43 (-0.51 to -0.35)	<0.0001
entorhinal	ENTORHINAL	Temporal	-0.35 (-0.44 to -0.27)	<0.0001
amygdala	AMYGDALA	Subcortical	-0.34 (-0.43 to -0.26)	<0.0001
parahippocampal gyrus	PARAHPCMPL	Temporal	-0.33 (-0.42 to -0.25)	<0.0001
temporal pole	TMPPOLE	Temporal	-0.28 (-0.37 to -0.19)	<0.0001
middle temporal gyrus	MIDTMP	Temporal	-0.28 (-0.37 to -0.19)	<0.0001
fusiform gyrus	FUSIFORM	Temporal	-0.28 (-0.36 to -0.19)	<0.0001
inferior temporal gyrus	INFRTMP	Temporal	-0.27 (-0.36 to -0.18)	<0.0001
inferior parietal cortex	INFRPRTL	Parietal	-0.25 (-0.34 to -0.16)	0.0002
banks of the superior temporal sulcus	SSTSBANK	Temporal	-0.19 (-0.29 to -0.10)	0.006
precuneus	PRECUNEUS	Parietal	-0.16 (-0.25 to -0.06)	0.04
superior parietal cortex	SUPERPRTL	Parietal	-0.15 (-0.24 to -0.05)	0.05
lingual gyrus	LINGUAL	Occipital	-0.15 (-0.24 to -0.05)	0.06
supramarginal gyrus	SUPRAMRGNL	Parietal	-0.13 (-0.22 to -0.03)	0.12
ventral diencephalon	VENTRALDC	Subcortical	-0.11 (-0.20 to -0.01)	0.21
putamen	PUTAMEN	Subcortical	-0.11 (-0.20 to -0.01)	0.21
frontal pole	FRNPOLE	Frontal	-0.10 (-0.20 to -0.01)	0.24
isthmus of the cingulate gyrus	ISTHMUSCNG	Parietal	-0.09 (-0.18 to 0.01)	0.33
paracentral gyrus	PARACNTRL	Frontal	0.09 (-0.01 to 0.18)	0.33
lateral occipital cortex	LATOCC	Occipital	-0.09 (-0.18 to 0.01)	0.33
pars orbitalis	PARSORBLS	Frontal	-0.08 (-0.18 to 0.01)	0.35
rostral anterior cingulate	ROSANTCNG	Frontal	-0.08 (-0.18 to 0.01)	0.35
pericalcarine cortex	PERICLCRN	Occipital	-0.07 (-0.17 to 0.02)	0.44
transverse temporal gyrus	TRANSTMP	Temporal	0.07 (-0.03 to 0.16)	0.45
insula	INSULA	Insula	-0.07 (-0.16 to 0.03)	0.45
cuneus	CUNEUS	Occipital	-0.07 (-0.16 to 0.03)	0.45
caudal anterior cingulate gyrus	CAUDANTCNG	Frontal	-0.06 (-0.16 to 0.03)	0.45
posterior cingulate gyrus	POSTCNG	Parietal	-0.06 (-0.16 to 0.03)	0.45
pallidum	PALLIDUM	Subcortical	0.06 (-0.04 to 0.16)	0.46
precentral gyrus	PRECNTRL	Frontal	0.05 (-0.04 to 0.15)	0.54
lateral orbitofrontal cortex	LATORBFRN	Frontal	-0.05 (-0.14 to 0.05)	0.60
caudate	CAUD	Subcortical	-0.04 (-0.14 to 0.05)	0.62
rostral middle frontal cortex	ROSMIDFRN	Frontal	-0.04 (-0.14 to 0.05)	0.62
superior temporal cortex	SUPERTMP	Temporal	-0.04 (-0.14 to 0.06)	0.62
thalamus	THALAMUS	Subcortical	-0.04 (-0.13 to 0.06)	0.62
postcentral gyrus	POSTCNTRL	Parietal	-0.03 (-0.12 to 0.07)	0.74
caudal middle frontal gyrus	CAUDMIDFRN	Frontal	-0.03 (-0.12 to 0.07)	0.74
pars triangularus	PARSTRNGLRS	Frontal	0.02 (-0.07 to 0.12)	0.79
pars opercularus	PARAOPRCLRS	Frontal	-0.02 (-0.11 to 0.08)	0.80
superior frontal cortex	SUPERFRN	Frontal	-0.02 (-0.11 to 0.08)	0.81
medial orbitalfrontal cortex	MEDORBFRN	Frontal	0.00 (-0.09 to 0.10)	0.94

Supplemental Table 21. Correlations between CSF pT181/T181 and regional brain volumes for amyloid PET positive individuals in the Knight ADRC amyloid PET cohort. The partial Spearman correlation after adjusting for age and sex are shown. All tests were two-sided and the significance of correlations was adjusted for comparisons with multiple regions using the Benjamini-Hochberg procedure.

Region of interest	Freesurfer label	Group	Partial Spearman ρ	Adjusted p-value
hippcampus	HIPPOCAMPUS	Subcortical	-0.18 (-0.27 to -0.08)	0.15
amygdala	AMYGDALA	Subcortical	-0.15 (-0.24 to -0.05)	0.34
parahippocampal gyrus	PARAHPCMPL	Temporal	-0.13 (-0.23 to -0.04)	0.40
temporal pole	TMPPOLE	Temporal	-0.13 (-0.22 to -0.03)	0.40
frontal pole	FRNPOLE	Frontal	-0.12 (-0.21 to -0.02)	0.42
entorhinal	ENTORHINAL	Temporal	-0.11 (-0.21 to -0.02)	0.42
precentral gyrus	PRECNTRL	Frontal	0.11 (0.01 to 0.20)	0.42
fusiform gyrus	FUSIFORM	Temporal	-0.11 (-0.20 to -0.01)	0.42
thalamus	THALAMUS	Subcortical	0.10 (0.00 to 0.20)	0.44
posterior cingulate gyrus	POSTCNG	Parietal	0.10 (0.00 to 0.19)	0.44
paracentral gyrus	PARACNTRL	Frontal	0.09 (0.00 to 0.19)	0.50
medial orbitalfrontal cortex	MEDORBFRN	Frontal	0.09 (-0.01 to 0.18)	0.53
pallidum	PALLIDUM	Subcortical	0.08 (-0.02 to 0.17)	0.65
pars triangularus	PARSTRNGLRS	Frontal	0.07 (-0.02 to 0.17)	0.65
superior temporal cortex	SUPERTMP	Temporal	0.07 (-0.03 to 0.16)	0.65
caudal middle frontal gyrus	CAUDMIDFRN	Frontal	0.07 (-0.03 to 0.16)	0.65
inferior temporal gyrus	INFRTMP	Temporal	-0.07 (-0.16 to 0.03)	0.65
middle temporal gyrus	MIDTMP	Temporal	-0.07 (-0.16 to 0.03)	0.65
ventral diencephalon	VENTRALDC	Subcortical	0.06 (-0.04 to 0.16)	0.71
inferior parietal cortex	INFRPRTL	Parietal	-0.05 (-0.15 to 0.04)	0.76
lingual gyrus	LINGUAL	Occipital	-0.05 (-0.15 to 0.04)	0.76
transverse temporal gyrus	TRANSTMP	Temporal	0.05 (-0.05 to 0.15)	0.78
postcentral gyrus	POSTCNTRL	Parietal	0.05 (-0.05 to 0.14)	0.82
isthmus of the cingulate gyrus	ISTHMUSCNG	Parietal	0.04 (-0.05 to 0.14)	0.85
lateral occipital cortex	LATOCC	Occipital	0.04 (-0.06 to 0.13)	0.88
cuneus	CUNEUS	Occipital	-0.04 (-0.13 to 0.06)	0.88
insula	INSULA	Insula	0.03 (-0.07 to 0.13)	0.88
superior parietal cortex	SUPERPRTL	Parietal	-0.03 (-0.13 to 0.07)	0.88
pars opercularus	PARAOPRCLRS	Frontal	0.03 (-0.07 to 0.12)	0.88
lateral orbitofrontal cortex	LATORBFRN	Frontal	0.03 (-0.07 to 0.12)	0.88
caudal anterior cingulate gyrus	CAUDANTCNG	Frontal	0.03 (-0.07 to 0.12)	0.88
pericalcarine cortex	PERICLCRN	Occipital	-0.02 (-0.12 to 0.07)	0.88
putamen	PUTAMEN	Subcortical	-0.02 (-0.12 to 0.07)	0.88
banks of the superior temporal sulcus	SSTSBANK	Temporal	-0.02 (-0.11 to 0.08)	0.89
rostral anterior cingulate	ROSANTCNG	Frontal	0.02 (-0.08 to 0.11)	0.89
rostral middle frontal cortex	ROSMIDFRN	Frontal	0.02 (-0.08 to 0.11)	0.89
superior frontal cortex	SUPERFRN	Frontal	0.02 (-0.08 to 0.11)	0.89
caudate	CAUD	Subcortical	-0.01 (-0.11 to 0.08)	0.89
supramarginal gyrus	SUPRAMRGNL	Parietal	-0.01 (-0.11 to 0.08)	0.89
pars orbitalis	PARSORBLS	Frontal	-0.01 (-0.10 to 0.09)	0.95
precuneus	PRECUNEUS	Parietal	0.00 (-0.10 to 0.1)	0.98

Supplemental Table 22. Correlations between CSF pT231/T231 and regional brain volumes for amyloid PET positive individuals in the Knight ADRC amyloid PET cohort. The partial Spearman correlation after adjusting for age and sex are shown. All tests were two-sided and the significance of correlations was adjusted for comparisons with multiple regions using the Benjamini-Hochberg procedure.

Region of interest	Freesurfer label	Group	Partial Spearman ρ	Adjusted p-value
fusiform gyrus	FUSIFORM	Temporal	-0.19 (-0.28 to -0.09)	0.17
parahippocampal gyrus	PARAHPCMPL	Temporal	-0.14 (-0.24 to -0.04)	0.48
hippcampus	HIPPOCAMPUS	Subcortical	-0.13 (-0.23 to -0.03)	0.48
inferior parietal cortex	INFRPRTL	Parietal	-0.13 (-0.22 to -0.03)	0.48
entorhinal	ENTORHINAL	Temporal	-0.12 (-0.22 to -0.02)	0.48
temporal pole	TMPPOLE	Temporal	-0.10 (-0.20 to 0.00)	0.50
pars triangularus	PARSTRNGLRS	Frontal	0.10 (0.00 to 0.20)	0.50
cuneus	CUNEUS	Occipital	-0.10 (-0.20 to 0.00)	0.50
thalamus	THALAMUS	Subcortical	0.10 (0.00 to 0.20)	0.50
lateral orbitofrontal cortex	LATORBFRN	Frontal	0.09 (-0.01 to 0.19)	0.50
middle temporal gyrus	MIDTMP	Temporal	-0.09 (-0.19 to 0.01)	0.50
rostral anterior cingulate	ROSANTCNG	Frontal	0.09 (-0.01 to 0.19)	0.50
banks of the superior temporal sulcus	SSTSBANK	Temporal	-0.09 (-0.19 to 0.01)	0.50
isthmus of the cingulate gyrus	ISTHMUSCNG	Parietal	0.09 (-0.01 to 0.19)	0.50
insula	INSULA	Insula	0.09 (-0.01 to 0.18)	0.50
inferior temporal gyrus	INFRTMP	Temporal	-0.08 (-0.18 to 0.02)	0.50
postcentral gyrus	POSTCNTRL	Parietal	0.08 (-0.02 to 0.18)	0.50
posterior cingulate gyrus	POSTCNG	Parietal	0.08 (-0.02 to 0.18)	0.50
precentral gyrus	PRECNTRL	Frontal	0.08 (-0.02 to 0.18)	0.50
transverse temporal gyrus	TRANSTMP	Temporal	0.08 (-0.02 to 0.18)	0.50
rostral middle frontal cortex	ROSMIDFRN	Frontal	0.07 (-0.03 to 0.17)	0.51
caudal middle frontal gyrus	CAUDMIDFRN	Frontal	0.06 (-0.05 to 0.15)	0.70
supramarginal gyrus	SUPRAMRGNL	Parietal	0.05 (-0.05 to 0.15)	0.70
lingual gyrus	LINGUAL	Occipital	-0.05 (-0.15 to 0.05)	0.70
pericalcarine cortex	PERICLCRN	Occipital	-0.05 (-0.15 to 0.05)	0.71
superior parietal cortex	SUPERPRTL	Parietal	-0.05 (-0.15 to 0.05)	0.71
amygdala	AMYGDALA	Subcortical	-0.05 (-0.15 to 0.05)	0.71
putamen	PUTAMEN	Subcortical	0.04 (-0.06 to 0.14)	0.72
superior frontal cortex	SUPERFRN	Frontal	0.04 (-0.06 to 0.14)	0.72
pallidum	PALLIDUM	Subcortical	0.04 (-0.06 to 0.14)	0.74
medial orbitalfrontal cortex	MEDORBFRN	Frontal	0.04 (-0.06 to 0.14)	0.76
superior temporal cortex	SUPERTMP	Temporal	0.03 (-0.07 to 0.14)	0.76
ventral diencephalon	VENTRALDC	Subcortical	-0.03 (-0.13 to 0.07)	0.79
lateral occipital cortex	LATOCC	Occipital	0.03 (-0.07 to 0.13)	0.79
caudate	CAUD	Subcortical	0.02 (-0.08 to 0.12)	0.83
caudal anterior cingulate gyrus	CAUDANTCNG	Frontal	-0.02 (-0.12 to 0.08)	0.83
precuneus	PRECUNEUS	Parietal	-0.02 (-0.12 to 0.08)	0.83
paracentral gyrus	PARACNTRL	Frontal	0.02 (-0.08 to 0.12)	0.84
pars orbitalis	PARSORBLS	Frontal	0.02 (-0.08 to 0.12)	0.84
pars opercularus	PARAOPRCLRS	Frontal	0.01 (-0.09 to 0.12)	0.84
frontal pole	FRNPOLE	Frontal	0.00 (-0.10 to 0.10)	0.98

Supplemental Table 23. Correspondence of CSF measures with clinical status in the Knight ADRC amyloid PET cohort. The receiver operating characteristic area under the curve for clinical status (cognitively unimpaired [CDR=0] or cognitively impaired [CDR>0] as predicted by biomarkers without and with covariates of age, sex, and years of education. CSF measures are shown in order of the best prediction of individuals who were cognitively impaired, stratified by measure type.

Analyte	AUC without covariates (95% CI)	AUC with covariates (95% CI)
**CSF Lumipulse measures**		
p-tau181 (pg/ml)	0.81 (0.76 to 0.85)	0.83 (0.79 to 0.87)
Aβ42/Aβ40	0.81 (0.77 to 0.85)	0.82 (0.78 to 0.86)
Total tau (pg/ml)	0.79 (0.74 to 0.83)	0.80 (0.76 to 0.84)
Aβ42 (pg/ml)	0.77 (0.72 to 0.82)	0.79 (0.75 to 0.84)
Aβ40 (pg/ml)	0.51 (0.46 to 0.57)	0.68 (0.64 to 0.73)
**Amyloid and tau PET measures**		
Tau PET summary measure	0.85 (0.78 to 0.91)	0.91 (0.86 to 0.95)
Amyloid PET Centiloid	0.83 (0.78 to 0.87)	0.86 (0.82 to 0.90)
**Phosphorylation occupancies by mass spectrometry**		
pT217/T217 (%)	0.84 (0.80 to 0.89)	0.87 (0.83 to 0.91)
pT111/T111 (%)	0.82 (0.78 to 0.86)	0.84 (0.80 to 0.88)
pT205/T205 (%)	0.81 (0.77 to 0.86)	0.85 (0.81 to 0.88)
pT153/T153 (%)	0.81 (0.77 to 0.85)	0.83 (0.79 to 0.87)
pS208/S208 (%)	0.80 (0.76 to 0.85)	0.83 (0.80 to 0.87)
pT181/T181 (%)	0.80 (0.75 to 0.85)	0.83 (0.79 to 0.87)
pT231/T231 (%)	0.79 (0.74 to 0.84)	0.81 (0.77 to 0.85)
pS202/S202 (%)	0.64 (0.59 to 0.70)	0.73 (0.68 to 0.78)
pS199/S199 (%)	0.57 (0.51 to 0.62)	0.70 (0.66 to 0.75)
pT175/T175 (%)	0.55 (0.49 to 0.60)	0.70 (0.65 to 0.74)
**Phosphorylated Non-phosphorylated tau concentrations by mass spectrometry**		
p-tau205 (pg/ml)	0.84 (0.80 to 0.88)	0.86 (0.83 to 0.90)
p-tau217 (pg/ml)	0.83 (0.79 to 0.88)	0.86 (0.82 to 0.90)
p-tau153 (pg/ml)	0.82 (0.78 to 0.86)	0.83 (0.79 to 0.86)
p-tau208 (pg/ml)	0.82 (0.77 to 0.86)	0.85 (0.81 to 0.88)
p-tau231 (pg/ml)	0.81 (0.76 to 0.85)	0.82 (0.78 to 0.86)
p-tau181 (pg/ml)	0.78 (0.73 to 0.82)	0.81 (0.77 to 0.85)
p-tau199 (pg/ml)	0.73 (0.68 to 0.78)	0.78 (0.74 to 0.82)
p-tau202 (pg/ml)	0.72 (0.67 to 0.77)	0.77 (0.73 to 0.81)
p-tau175 (pg/ml)	0.65 (0.60 to 0.71)	0.72 (0.68 to 0.77)
**Non-phosphorylated tau concentrations by mass spectrometry**		
Tau195-210 (ng/ml)	0.75 (0.70 to 0.80)	0.79 (0.75 to 0.84)
Tau226-230 (ng/ml)	0.75 (0.70 to 0.79)	0.78 (0.73 to 0.82)
Tau151-155 (ng/ml)	0.74 (0.70 to 0.79)	0.78 (0.74 to 0.83)
Tau181-190 (ng/ml)	0.73 (0.68 to 0.78)	0.78 (0.74 to 0.82)
Tau212-221 (ng/ml)	0.72 (0.67 to 0.77)	0.77 (0.73 to 0.81)

Supplemental Table 24. Correspondence of CSF measures with clinical status in the amyloid PET positive individuals in the Knight ADRC amyloid PET cohort. The receiver operating characteristic area under the curve for clinical status (cognitively unimpaired [CDR=0] or cognitively impaired [CDR>0] as predicted by biomarkers without and with covariates of age, sex, and years of education. CSF measures are shown in order of the best prediction of individuals who were cognitively impaired, stratified by measure type.

Analyte	AUC without covariates (95% CI)	AUC with covariates (95% CI)
**CSF Lumipulse measures**		
p-tau181 (pg/ml)	0.69 (0.62 to 0.75)	0.71 (0.65 to 0.78)
Aβ42 (pg/ml)	0.68 (0.61 to 0.74)	0.71 (0.64 to 0.77)
Total tau (pg/ml)	0.66 (0.59 to 0.73)	0.70 (0.63 to 0.76)
Aβ42/Aβ40	0.64 (0.57 to 0.71)	0.68 (0.62 to 0.75)
Aβ40 (pg/ml)	0.60 (0.53 to 0.67)	0.66 (0.59 to 0.72)
**Amyloid and tau PET measures**		
Tau PET summary measure	0.86 (0.78 to 0.94)	0.89 (0.82 to 0.95)
Amyloid PET Centiloid	0.77 (0.71 to 0.83)	0.79 (0.74 to 0.85)
**Phosphorylation occupancies by mass spectrometry**		
pT217/T217 (%)	0.80 (0.74 to 0.85)	0.82 (0.76 to 0.87)
pT205/T205 (%)	0.79 (0.73 to 0.84)	0.81 (0.75 to 0.86)
pT111/T111 (%)	0.69 (0.62 to 0.75)	0.71 (0.64 to 0.77)
pS208/S208 (%)	0.68 (0.61 to 0.74)	0.71 (0.65 to 0.77)
pT181/T181 (%)	0.67 (0.61 to 0.74)	0.69 (0.63 to 0.76)
pT153/T153 (%)	0.64 (0.58 to 0.71)	0.68 (0.62 to 0.75)
pT231/T231 (%)	0.61 (0.54 to 0.68)	0.64 (0.57 to 0.71)
pS202/S202 (%)	0.57 (0.50 to 0.64)	0.63 (0.56 to 0.69)
pT175/T175 (%)	0.56 (0.48 to 0.63)	0.61 (0.54 to 0.68)
pS199/S199 (%)	0.52 (0.45 to 0.59)	0.62 (0.55 to 0.69)
**Phosphorylated Non-phosphorylated tau concentrations by mass spectrometry**		
p-tau205 (pg/ml)	0.78 (0.72 to 0.84)	0.80 (0.74 to 0.85)
p-tau217 (pg/ml)	0.75 (0.69 to 0.81)	0.76 (0.70 to 0.82)
p-tau208 (pg/ml)	0.71 (0.65 to 0.78)	0.73 (0.66 to 0.79)
p-tau231 (pg/ml)	0.66 (0.59 to 0.73)	0.66 (0.59 to 0.73)
p-tau153 (pg/ml)	0.65 (0.58 to 0.72)	0.68 (0.61 to 0.74)
p-tau181 (pg/ml)	0.65 (0.58 to 0.71)	0.68 (0.61 to 0.74)
p-tau199 (pg/ml)	0.63 (0.56 to 0.70)	0.67 (0.60 to 0.73)
p-tau202 (pg/ml)	0.63 (0.56 to 0.70)	0.67 (0.60 to 0.73)
p-tau175 (pg/ml)	0.55 (0.48 to 0.62)	0.62 (0.55 to 0.69)
**Non-phosphorylated tau concentrations by mass spectrometry**		
Tau195-210 (ng/ml)	0.64 (0.57 to 0.70)	0.68 (0.62 to 0.75)
Tau226-230 (ng/ml)	0.62 (0.55 to 0.69)	0.67 (0.60 to 0.73)
Tau151-155 (ng/ml)	0.61 (0.54 to 0.68)	0.66 (0.59 to 0.72)
Tau181-190 (ng/ml)	0.61 (0.54 to 0.68)	0.66 (0.60 to 0.73)
Tau212-221 (ng/ml)	0.60 (0.54 to 0.67)	0.66 (0.59 to 0.72)

Supplemental Table 25. Correlations between biomarker measures and Clinical Dementia Rating Sum of Boxes for the Knight ADRC amyloid PET cohort. The Spearman correlation and partial Spearman correlation after adjusting for age, sex, and years of education are shown. CSF measures are shown in order of the absolute value of their correlation with Clinical Dementia Rating Sum of Boxes, stratified by measure type. All tests were two-sided and were not adjusted for multiple comparisons.

Measure	n=	Spearman ρ	p=	Partial Spearman ρ	p=
**CSF Lumipulse measures**					
p-tau181 (pg/ml)	750	0.39 (0.33 to 0.45)	<0.0001	0.34 (0.28 to 0.40)	<0.0001
Aβ42/Aβ40	750	-0.38 (-0.44 to -0.32)	<0.0001	-0.34 (-0.40 to -0.28)	<0.0001
Total tau (pg/ml)	750	0.36 (0.30 to 0.42)	<0.0001	0.32 (0.25 to 0.38)	<0.0001
Aβ42 (pg/ml)	750	-0.34 (-0.40 to -0.27)	<0.0001	-0.31 (-0.37 to -0.24)	<0.0001
Aβ40 (pg/ml)	750	-0.02 (-0.09 to 0.05)	0.60	-0.05 (-0.12 to 0.03)	0.21
**Amyloid and tau PET measures**					
Tau PET summary measure	361	0.43 (0.35 to 0.51)	<0.0001	0.42 (0.33 to 0.50)	<0.0001
Amyloid PET Centiloid	750	0.39 (0.33 to 0.45)	<0.0001	0.36 (0.29 to 0.42)	<0.0001
**Phosphorylation occupancies by mass spectrometry**					
pT217/T217 (%)	750	0.43 (0.37 to 0.48)	<0.0001	0.38 (0.31 to 0.44)	<0.0001
pT111/T111 (%)	746	0.40 (0.33 to 0.46)	<0.0001	0.35 (0.29 to 0.41)	<0.0001
pT153/T153 (%)	660	0.39 (0.33 to 0.46)	<0.0001	0.35 (0.29 to 0.42)	<0.0001
pT205/T205 (%)	749	0.39 (0.33 to 0.45)	<0.0001	0.34 (0.28 to 0.40)	<0.0001
pS208/S208 (%)	685	0.38 (0.32 to 0.45)	<0.0001	0.34 (0.27 to 0.40)	<0.0001
pT231/T231 (%)	620	0.38 (0.31 to 0.44)	<0.0001	0.34 (0.26 to 0.40)	<0.0001
pT181/T181 (%)	747	0.36 (0.30 to 0.42)	<0.0001	0.32 (0.25 to 0.38)	<0.0001
pS202/S202 (%)	750	-0.18 (-0.25 to -0.11)	<0.0001	-0.16 (-0.23 to -0.09)	<0.0001
pT175/T175 (%)	734	-0.08 (-0.15 to -0.01)	0.03	-0.08 (-0.15 to -0.01)	0.03
pS199/S199 (%)	743	0.08 (0.00 to 0.15)	0.04	0.04 (-0.04 to 0.11)	0.31
**Phosphorylated tau concentrations by mass spectrometry**					
p-tau205 (pg/ml)	749	0.43 (0.37 to 0.49)	<0.0001	0.38 (0.32 to 0.44)	<0.0001
p-tau217 (pg/ml)	750	0.42 (0.36 to 0.48)	<0.0001	0.38 (0.31 to 0.44)	<0.0001
p-tau153 (pg/ml)	660	0.41 (0.34 to 0.47)	<0.0001	0.37 (0.30 to 0.43)	<0.0001
p-tau208 (pg/ml)	685	0.40 (0.34 to 0.46)	<0.0001	0.35 (0.29 to 0.42)	<0.0001
p-tau231 (pg/ml)	619	0.40 (0.33 to 0.46)	<0.0001	0.36 (0.29 to 0.43)	<0.0001
p-tau181 (pg/ml)	747	0.35 (0.29 to 0.42)	<0.0001	0.31 (0.24 to 0.37)	<0.0001
p-tau199 (pg/ml)	743	0.29 (0.22 to 0.35)	<0.0001	0.23 (0.16 to 0.30)	<0.0001
p-tau202 (pg/ml)	750	0.28 (0.22 to 0.35)	<0.0001	0.23 (0.16 to 0.30)	<0.0001
p-tau175 (pg/ml)	734	0.19 (0.12 to 0.26)	<0.0001	0.15 (0.08 to 0.22)	<0.0001
**Non-phosphorylated tau concentrations by mass spectrometry**					
Tau195-210 (ng/ml)	750	0.33 (0.26 to 0.39)	<0.0001	0.28 (0.21 to 0.35)	<0.0001
Tau151-155 (ng/ml)	747	0.32 (0.25 to 0.38)	<0.0001	0.27 (0.20 to 0.34)	<0.0001
Tau226-230 (ng/ml)	719	0.31 (0.24 to 0.37)	<0.0001	0.26 (0.19 to 0.33)	<0.0001
Tau181-190 (ng/ml)	750	0.30 (0.23 to 0.36)	<0.0001	0.25 (0.19 to 0.32)	<0.0001
Tau212-221 (ng/ml)	750	0.29 (0.22 to 0.36)	<0.0001	0.24 (0.17 to 0.31)	<0.0001

Supplemental Table 26. Correlations between biomarker measures and Clinical Dementia Rating Sum of Boxes for amyloid PET positive individuals in the Knight ADRC amyloid PET cohort. The Spearman correlation and partial Spearman correlation after adjusting for age, sex, and years of education are shown. CSF measures are shown in order of the absolute value of their correlation with Clinical Dementia Rating Sum of Boxes, stratified by measure type. All tests were two-sided and were not adjusted for multiple comparisons.

Measure	n=	Spearman ρ	p=	Partial Spearman ρ	p=
**CSF Lumipulse measures**					
p-tau181 (pg/ml)	263	0.33 (0.22 to 0.43)	<0.0001	0.32 (0.21 to 0.42)	<0.0001
Aβ42 (pg/ml)	263	-0.30 (-0.40 to -0.18)	<0.0001	-0.28 (-0.38 to -0.16)	<0.0001
Total tau (pg/ml)	263	0.29 (0.18 to 0.40)	<0.0001	0.28 (0.17 to 0.39)	<0.0001
Aβ42/Aβ40	263	-0.23 (-0.34 to -0.12)	0.0001	-0.22 (-0.33 to -0.10)	0.0004
Aβ40 (pg/ml)	263	-0.17 (-0.28 to -0.05)	0.006	-0.16 (-0.27 to -0.03)	0.01
**Amyloid and tau PET measures**					
Tau PET summary measure	124	0.65 (0.54 to 0.74)	<0.0001	0.65 (0.54 to 0.74)	<0.0001
Amyloid PET Centiloid	263	0.42 (0.31 to 0.51)	<0.0001	0.42 (0.32 to 0.52)	<0.0001
**Phosphorylation occupancies by mass spectrometry**					
pT217/T217 (%)	263	0.50 (0.41 to 0.59)	<0.0001	0.49 (0.39 to 0.58)	<0.0001
pT205/T205 (%)	263	0.50 (0.40 to 0.59)	<0.0001	0.48 (0.38 to 0.57)	<0.0001
pT111/T111 (%)	263	0.32 (0.21 to 0.43)	<0.0001	0.30 (0.18 to 0.40)	<0.0001
pS208/S208 (%)	260	0.31 (0.20 to 0.42)	<0.0001	0.28 (0.16 to 0.39)	<0.0001
pT181/T181 (%)	262	0.28 (0.16 to 0.39)	<0.0001	0.26 (0.14 to 0.37)	<0.0001
pT153/T153 (%)	261	0.23 (0.11 to 0.34)	0.0002	0.22 (0.11 to 0.34)	0.0003
pT231/T231 (%)	240	0.20 (0.08 to 0.32)	0.0015	0.20 (0.07 to 0.32)	0.002
pT175/T175 (%)	257	-0.13 (-0.25 to -0.01)	0.04	-0.12 (-0.24 to 0.01)	0.06
pS202/S202 (%)	263	-0.12 (-0.23 to 0.00)	0.06	-0.13 (-0.25 to -0.01)	0.03
pS199/S199 (%)	263	0.06 (-0.06 to 0.18)	0.33	0.02 (-0.10 to 0.15)	0.69
**Phosphorylated tau concentrations by mass spectrometry**					
p-tau205 (pg/ml)	263	0.49 (0.40 to 0.58)	<0.0001	0.47 (0.37 to 0.56)	<0.0001
p-tau217 (pg/ml)	263	0.44 (0.34 to 0.53)	<0.0001	0.43 (0.32 to 0.52)	<0.0001
p-tau208 (pg/ml)	260	0.38 (0.27 to 0.48)	<0.0001	0.35 (0.24 to 0.46)	<0.0001
p-tau231 (pg/ml)	239	0.29 (0.17 to 0.40)	<0.0001	0.28 (0.16 to 0.39)	<0.0001
p-tau181 (pg/ml)	262	0.26 (0.15 to 0.37)	<0.0001	0.25 (0.13 to 0.36)	<0.0001
p-tau153 (pg/ml)	261	0.25 (0.14 to 0.36)	<0.0001	0.25 (0.13 to 0.36)	<0.0001
p-tau199 (pg/ml)	263	0.24 (0.12 to 0.35)	<0.0001	0.21 (0.10 to 0.33)	0.0005
p-tau202 (pg/ml)	263	0.24 (0.12 to 0.35)	<0.0001	0.22 (0.10 to 0.33)	0.0004
p-tau175 (pg/ml)	257	0.08 (-0.04 to 0.20)	0.19	0.09 (-0.03 to 0.21)	0.15
**Non-phosphorylated tau concentrations by mass spectrometry**					
Tau195-210 (ng/ml)	263	0.25 (0.13 to 0.36)	<0.0001	0.25 (0.13 to 0.36)	<0.0001
Tau226-230 (ng/ml)	259	0.23 (0.11 to 0.34)	0.0002	0.22 (0.10 to 0.33)	0.0004
Tau181-190 (ng/ml)	263	0.21 (0.09 to 0.32)	0.0007	0.20 (0.08 to 0.32)	0.0009
Tau212-221 (ng/ml)	263	0.20 (0.09 to 0.32)	0.0008	0.20 (0.08 to 0.31)	0.001
Tau151-155 (ng/ml)	261	0.20 (0.08 to 0.31)	0.001	0.19 (0.07 to 0.31)	0.002

Supplemental Table 27. CSF tau measures for cognitively impaired individuals in the Knight ADRC tau PET cohort. Continuous values are presented as the median with the interquartile range. The significance of differences by amyloid PET status were evaluated with Wilcoxon ranked sum tests for continuous variables and Chi-Square or Fisher exact tests for categorical variables. The fold difference is the median biomarker value in the tau PET positive group divided by the median value in the tau PET negative group. All tests were two-sided and were not adjusted for multiple comparisons.

Characteristic	All (n=55)	Tau PET negative (n=20)	Tau PET positive (n=35)	Fold Change	p=
**Phosphorylation occupancies by mass spectrometry**					
pT111/T111 (%)	8.66 (6.29-10.5)	4.93 (3.56-8.4)	9.73 (8.13-11.1)	1.97	0.0002
pT153/T153 (%)	0.144 (0.0934-0.209)	0.0889 (0.0613-0.111)	0.175 (0.142-0.211)	1.97	0.0003
pT175/T175 (%)	0.427 (0.367-0.501)	0.458 (0.395-0.557)	0.426 (0.34-0.458)	0.93	0.08
pT181/T181 (%)	36.2 (32-37.9)	32.0 (27.9-34.2)	36.8 (35.3-40.2)	1.15	0.0004
pS199/S199 (%)	0.695 (0.557-0.893)	0.613 (0.465-0.893)	0.726 (0.617-0.891)	1.18	0.30
pS202/S202 (%)	4.76 (4.28-5.95)	5.65 (4.45-6.92)	4.62 (3.92-5.19)	0.82	0.02
pT205/T205 (%)	1.40 (1.19-1.7)	1.06 (0.804-1.36)	1.60 (1.38-1.81)	1.51	<0.0001
pS208/S208 (%)	0.272 (0.209-0.331)	0.196 (0.112-0.283)	0.282 (0.259-0.36)	1.44	0.001
pT217/T217 (%)	11.9 (7.69-13.3)	5.69 (3.54-7.96)	12.8 (11.8-13.9)	2.25	<0.0001
pT231/T231 (%)	26.9 (17.7-31.5)	13.6 (6.99-26.7)	27.4 (24.3-35.4)	2.01	0.0005
**Phosphorylated tau concentrations by mass spectrometry**					
p-tau153 (pg/ml)	4.49 (2.53-7.11)	2.22 (1.14-4.83)	4.78 (4.15-7.67)	2.15	0.002
p-tau175 (pg/ml)	13.4 (10.1-17.2)	13.0 (7.57-19.6)	13.4 (10.2-15.9)	1.03	0.96
p-tau181 (pg/ml)	1160 (789-1470)	789 (643-1180)	1220 (944-1530)	1.55	0.009
p-tau199 (pg/ml)	25.5 (16.1-34.5)	21.1 (14.8-27.9)	27.2 (21.8-34.9)	1.29	0.03
p-tau202 (pg/ml)	176 (139-214)	148 (121-190)	176 (141-214)	1.19	0.38
p-tau205 (pg/ml)	50.0 (35.3-69.0)	30.5 (21.3-43.5)	63.8 (45.4-74.3)	2.09	<0.0001
p-tau208 (pg/ml)	10.0 (6.18-13.8)	5.94 (3.19-9.52)	11.3 (9.04-15.2)	1.9	0.0003
p-tau217 (pg/ml)	254 (178-333)	136 (64.6-188)	300 (231-385)	2.21	<0.0001
p-tau231 (pg/ml)	88.4 (53.5-127)	33.8 (15.5-71.9)	103 (84.2-147)	3.05	<0.0001
**Non-phosphorylated tau concentrations by mass spectrometry**					
Tau151-155 (ng/ml)	2.80 (2.27-3.59)	2.49 (1.93-3.21)	3.21 (2.29-3.67)	1.29	0.09
Tau181-190 (ng/ml)	3.18 (2.34-3.92)	2.85 (1.91-3.21)	3.40 (2.42-3.95)	1.19	0.08
Tau195-210 (ng/ml)	3.44 (2.76-4.44)	3.05 (2.3-3.44)	3.91 (2.95-4.53)	1.28	0.02
Tau212-221 (ng/ml)	2.33 (1.74-2.78)	2.06 (1.47-2.36)	2.51 (1.78-2.81)	1.22	0.14
Tau226-230 (ng/ml)	0.325 (0.239-0.477)	0.243 (0.216-0.31)	0.363 (0.289-0.51)	1.49	0.006

Supplemental Table 28. Correlations between CSF biomarkers and amyloid PET Centiloid for cognitively impaired individuals in the Knight ADRC tau PET cohort. The Spearman correlation and partial Spearman correlation after adjusting for age and sex are shown. CSF measures are shown in order of the absolute value of their correlation with amyloid PET Centiloid, stratified by measure type. All tests were two-sided and were not adjusted for multiple comparisons.

Measure	Spearman ρ	p=	Partial Spearman ρ	p=
**CSF Lumipulse measures**				
Aβ42 (pg/ml)	-0.61 (-0.75 to -0.41)	<0.0001	-0.61 (-0.76 to -0.41)	<0.0001
Aβ42/Aβ40	-0.58 (-0.73 to -0.37)	<0.0001	-0.57 (-0.73 to -0.35)	<0.0001
p-tau181 (pg/ml)	0.47 (0.24 to 0.65)	0.0002	0.46 (0.22 to 0.65)	0.0004
Total tau (pg/ml)	0.26 (-0.01 to 0.49)	0.05	0.24 (-0.03 to 0.48)	0.08
Aβ40 (pg/ml)	-0.24 (-0.48 to 0.02)	0.07	-0.27 (-0.50 to 0.00)	0.05
**Phosphorylation occupancies by mass spectrometry**				
pT217/T217 (%)	0.77 (0.63 to 0.86)	<0.0001	0.78 (0.65 to 0.87)	<0.0001
pT111/T111 (%)	0.66 (0.47 to 0.78)	<0.0001	0.66 (0.47 to 0.79)	<0.0001
pS208/S208 (%)	0.62 (0.42 to 0.76)	<0.0001	0.62 (0.42 to 0.76)	<0.0001
pT181/T181 (%)	0.59 (0.38 to 0.74)	<0.0001	0.58 (0.37 to 0.74)	<0.0001
pT231/T231 (%)	0.59 (0.37 to 0.75)	<0.0001	0.58 (0.35 to 0.75)	<0.0001
pT205/T205 (%)	0.57 (0.36 to 0.73)	<0.0001	0.62 (0.42 to 0.76)	<0.0001
pT153/T153 (%)	0.39 (0.13 to 0.60)	0.004	0.40 (0.14 to 0.61)	0.004
pT175/T175 (%)	-0.36 (-0.57 to -0.10)	0.006	-0.38 (-0.59 to -0.12)	0.004
pS202/S202 (%)	-0.19 (-0.43 to 0.08)	0.16	-0.19 (-0.44 to 0.09)	0.17
pS199/S199 (%)	0.09 (-0.18 to 0.35)	0.49	0.1 (-0.17 to 0.36)	0.46
**Phosphorylated tau concentrations by mass spectrometry**				
p-tau217 (pg/ml)	0.62 (0.42 to 0.76)	<0.0001	0.61 (0.41 to 0.76)	<0.0001
p-tau205 (pg/ml)	0.61 (0.41 to 0.75)	<0.0001	0.61 (0.4 to 0.75)	<0.0001
p-tau208 (pg/ml)	0.55 (0.34 to 0.71)	<0.0001	0.54 (0.32 to 0.71)	<0.0001
p-tau231 (pg/ml)	0.53 (0.29 to 0.71)	<0.0001	0.52 (0.27 to 0.71)	0.0001
p-tau181 (pg/ml)	0.30 (0.04 to 0.53)	0.02	0.28 (0.01 to 0.51)	0.04
p-tau153 (pg/ml)	0.30 (0.03 to 0.53)	0.03	0.30 (0.02 to 0.53)	0.03
p-tau202 (pg/ml)	0.23 (-0.03 to 0.47)	0.08	0.21 (-0.06 to 0.45)	0.13
p-tau199 (pg/ml)	0.20 (-0.07 to 0.44)	0.14	0.19 (-0.09 to 0.43)	0.18
**Non-phosphorylated tau concentrations by mass spectrometry**				
Tau195-209 (ng/ml)	0.27 (0.01 to 0.5)	0.04	0.25 (-0.02 to 0.49)	0.07
Tau226-230 (ng/ml)	0.27 (0.01 to 0.5)	0.04	0.26 (-0.01 to 0.5)	0.06
Tau151-155 (ng/ml)	0.18 (-0.09 to 0.43)	0.18	0.15 (-0.12 to 0.41)	0.27
Tau181-190 (ng/ml)	0.16 (-0.11 to 0.41)	0.23	0.14 (-0.14 to 0.39)	0.33
Tau212-221 (ng/ml)	0.15 (-0.12 to 0.4)	0.28	0.12 (-0.15 to 0.38)	0.38

Supplemental Table 29. Correlations between CSF biomarkers and the tau PET summary measure for cognitively impaired individuals in the Knight ADRC tau PET cohort. The Spearman correlation and partial Spearman correlation after adjusting for age and sex are shown. CSF measures are shown in order of the absolute value of their correlation with the Tau PET Summary Measure, stratified by measure type. All tests were two-sided and were not adjusted for multiple comparisons.

Measure	Spearman ρ	p=	Partial Spearman ρ	p=
**CSF Lumipulse measures**				
Aβ42 (pg/ml)	-0.59 (-0.74 to -0.39)	<0.0001	-0.61 (-0.76 to -0.41)	<0.0001
Aβ42/Aβ40	-0.48 (-0.66 to -0.25)	0.0001	-0.53 (-0.70 to -0.30)	<0.0001
p-tau181 (pg/ml)	0.44 (0.20 to 0.63)	0.0005	0.50 (0.26 to 0.68)	0.0001
Aβ40 (pg/ml)	-0.33 (-0.54 to -0.07)	0.01	-0.33 (-0.55 to -0.06)	0.01
Total tau (pg/ml)	0.32 (0.06 to 0.54)	0.01	0.39 (0.13 to 0.60)	0.003
**Phosphorylation occupancies by mass spectrometry**				
pT217/T217 (%)	0.71 (0.55 to 0.82)	<0.0001	0.73 (0.58 to 0.84)	<0.0001
pT205/T205 (%)	0.67 (0.50 to 0.80)	<0.0001	0.66 (0.47 to 0.79)	<0.0001
pS208/S208 (%)	0.49 (0.26 to 0.67)	0.0001	0.52 (0.29 to 0.69)	<0.0001
pT231/T231 (%)	0.48 (0.22 to 0.67)	0.0004	0.52 (0.27 to 0.7)	0.0001
pT153/T153 (%)	0.46 (0.21 to 0.65)	0.0004	0.51 (0.27 to 0.69)	0.0001
pT111/T111 (%)	0.45 (0.21 to 0.64)	0.0004	0.48 (0.25 to 0.67)	0.0002
pT181/T181 (%)	0.42 (0.18 to 0.62)	0.001	0.47 (0.23 to 0.66)	0.0003
pT175/175 (%)	-0.26 (-0.49 to 0.01)	0.05	-0.22 (-0.47 to 0.05)	0.10
pS202/S202 (%)	-0.20 (-0.44 to 0.07)	0.14	-0.26 (-0.5 to 0.01)	0.06
pS199/S199 (%)	0.15 (-0.12 to 0.4)	0.26	0.15 (-0.12 to 0.4)	0.28
**Phosphorylated tau concentrations by mass spectrometry**				
p-tau205 (pg/ml)	0.62 (0.42 to 0.76)	<0.0001	0.64 (0.44 to 0.77)	<0.0001
p-tau217 (pg/ml)	0.59 (0.38 to 0.74)	<0.0001	0.64 (0.44 to 0.77)	<0.0001
p-tau231 (pg/ml)	0.53 (0.29 to 0.71)	<0.0001	0.57 (0.33 to 0.74)	<0.0001
p-tau208 (pg/ml)	0.47 (0.23 to 0.65)	0.0002	0.54 (0.31 to 0.7)	<0.0001
p-tau153 (pg/ml)	0.36 (0.09 to 0.58)	0.008	0.42 (0.16 to 0.63)	0.002
p-tau181 (pg/ml)	0.30 (0.03 to 0.52)	0.03	0.38 (0.12 to 0.59)	0.004
p-tau199 (pg/ml)	0.26 (-0.01 to 0.49)	0.05	0.28 (0.01 to 0.51)	0.04
p-tau202 (pg/ml)	0.13 (-0.14 to 0.38)	0.34	0.16 (-0.11 to 0.42)	0.24
p-tau175 (pg/ml)	-0.04 (-0.3 to 0.23)	0.79	0.03 (-0.24 to 0.3)	0.84
**Non-phosphorylated tau concentrations by mass spectrometry**				
Tau226-230 (ng/ml)	0.31 (0.04 to 0.53)	0.02	0.33 (0.07 to 0.56)	0.01
Tau195-209 (ng/ml)	0.26 (-0.01 to 0.49)	0.06	0.32 (0.06 to 0.55)	0.02
Tau181-190 (ng/ml)	0.16 (-0.11 to 0.41)	0.23	0.23 (-0.04 to 0.47)	0.10
Tau151-155 (ng/ml)	0.15 (-0.12 to 0.4)	0.28	0.20 (-0.07 to 0.45)	0.14
Tau212-221 (ng/ml)	0.14 (-0.13 to 0.39)	0.29	0.20 (-0.07 to 0.45)	0.14

Supplemental Table 30. Correlations between CSF biomarkers and amyloid PET Centiloid for individuals in the BioFINDER-2 cohort. The Spearman correlation and partial Spearman correlation after adjusting for age and sex are shown. CSF measures are shown in order of the absolute value of their correlation with amyloid PET Centiloid, stratified by measure type. All tests were two-sided and were not adjusted for multiple comparisons.

Measure	Spearman ρ	p=	Partial Spearman ρ	p=
**CSF NeuroTool Kit measures**				
p-tau181 (pg/ml)	0.24 (0.03 to 0.42)	0.02	0.21 (0.00 to 0.40)	0.05
Aβ42/Aβ40	-0.19 (-0.38 to 0.02)	0.08	-0.18 (-0.37 to 0.03)	0.10
Total tau (pg/ml)	0.16 (-0.05 to 0.35)	0.14	0.11 (-0.10 to 0.32)	0.29
Aβ42 (pg/ml)	-0.13 (-0.33 to 0.08)	0.21	-0.18 (-0.37 to 0.03)	0.10
Aβ40 (pg/ml)	-0.06 (-0.27 to 0.15)	0.56	-0.12 (-0.32 to 0.09)	0.26
**Phosphorylation occupancies by mass spectrometry**				
pT217/T217 (%)	0.55 (0.38 to 0.68)	<0.0001	0.58 (0.42 to 0.70)	<0.0001
pT181/T181 (%)	0.50 (0.33 to 0.64)	<0.0001	0.52 (0.34 to 0.65)	<0.0001
pT153/T153 (%)	0.49 (0.32 to 0.63)	<0.0001	0.53 (0.36 to 0.67)	<0.0001
pT111/T111 (%)	0.46 (0.28 to 0.61)	<0.0001	0.47 (0.28 to 0.61)	<0.0001
pT205/T205 (%)	0.44 (0.26 to 0.60)	<0.0001	0.51 (0.33 to 0.65)	<0.0001
pS208/S208 (%)	0.43 (0.25 to 0.59)	<0.0001	0.47 (0.29 to 0.62)	<0.0001
pT231/T231 (%)	0.43 (0.25 to 0.59)	<0.0001	0.46 (0.27 to 0.61)	<0.0001
pS199/S199 (%)	0.11 (-0.10 to 0.31)	0.31	0.15 (-0.06 to 0.35)	0.17
pS202/S202 (%)	-0.09 (-0.29 to 0.12)	0.38	-0.07 (-0.27 to 0.14)	0.52
**Phosphorylated tau concentrations by mass spectrometry**				
p-tau205 (pg/ml)	0.47 (0.29 to 0.62)	<0.0001	0.48 (0.31 to 0.63)	<0.0001
p-tau217 (pg/ml)	0.45 (0.26 to 0.60)	<0.0001	0.45 (0.26 to 0.60)	<0.0001
p-tau208 (pg/ml)	0.44 (0.26 to 0.60)	<0.0001	0.44 (0.26 to 0.60)	<0.0001
p-tau153 (pg/ml)	0.43 (0.25 to 0.59)	<0.0001	0.43 (0.24 to 0.59)	<0.0001
p-tau231 (pg/ml)	0.36 (0.16 to 0.53)	0.0004	0.35 (0.15 to 0.52)	0.0008
p-tau181 (pg/ml)	0.31 (0.11 to 0.49)	0.003	0.29 (0.09 to 0.47)	0.006
p-tau199 (pg/ml)	0.28 (0.07 to 0.46)	0.008	0.26 (0.06 to 0.45)	0.01
p-tau202 (pg/ml)	0.20 (0.00 to 0.39)	0.05	0.18 (-0.03 to 0.37)	0.10
**Non-phosphorylated tau concentrations by mass spectrometry**				
Tau195-210 (ng/ml)	0.22 (0.02 to 0.41)	0.03	0.19 (-0.02 to 0.38)	0.08
Tau151-155 (ng/ml)	0.18 (-0.02 to 0.38)	0.08	0.15 (-0.06 to 0.35)	0.16
Tau181-190 (ng/ml)	0.17 (-0.04 to 0.37)	0.10	0.13 (-0.08 to 0.33)	0.21
Tau226-230 (ng/ml)	0.17 (-0.04 to 0.36)	0.11	0.13 (-0.08 to 0.33)	0.23
Tau212-221 (ng/ml)	0.16 (-0.05 to 0.36)	0.12	0.11 (-0.10 to 0.32)	0.29

Supplemental Table 31. Correlations between CSF biomarkers and tau PET for Braak I-IV regions for individuals in BioFINDER-2 cohort. The Spearman correlation and partial Spearman correlation after adjusting for age and sex are shown. CSF measures are shown in order of the absolute value of their correlation with tau PET, stratified by measure type. All tests were two-sided and were not adjusted for multiple comparisons.

Measure	Spearman ρ	p=	Partial Spearman ρ	p=
**CSF NeuroTool Kit measures**				
p-tau181 (pg/ml)	0.42 (0.23 to 0.57)	<0.0001	0.44 (0.26 to 0.60)	<0.0001
Aβ42/Aβ40	-0.33 (-0.50 to -0.13)	0.002	-0.33 (-0.50 to -0.13)	0.002
Total tau (pg/ml)	0.32 (0.13 to 0.50)	0.002	0.36 (0.16 to 0.53)	0.0005
Aβ42 (pg/ml)	-0.22 (-0.41 to -0.02)	0.03	-0.23 (-0.42 to -0.02)	0.03
Aβ40 (pg/ml)	-0.03 (-0.24 to 0.18)	0.77	-0.02 (-0.23 to 0.19)	0.83
**Phosphorylation occupancies by mass spectrometry**				
pT217/T217 (%)	0.76 (0.65 to 0.83)	<0.0001	0.76 (0.65 to 0.83)	<0.0001
pT205/T205 (%)	0.72 (0.60 to 0.81)	<0.0001	0.72 (0.60 to 0.81)	<0.0001
pT181/T181 (%)	0.59 (0.43 to 0.71)	<0.0001	0.59 (0.43 to 0.71)	<0.0001
pS208/S208 (%)	0.57 (0.41 to 0.70)	<0.0001	0.57 (0.40 to 0.69)	<0.0001
pT231/T231 (%)	0.48 (0.31 to 0.63)	<0.0001	0.48 (0.30 to 0.63)	<0.0001
pT153/T153 (%)	0.42 (0.24 to 0.58)	<0.0001	0.42 (0.23 to 0.58)	<0.0001
pT111/T111 (%)	0.33 (0.13 to 0.50)	0.001	0.33 (0.13 to 0.50)	0.002
pS199/S199 (%)	0.14 (-0.07 to 0.33)	0.20	0.13 (-0.08 to 0.33)	0.22
pS202/S202 (%)	-0.12 (-0.32 to 0.09)	0.25	-0.13 (-0.33 to 0.09)	0.24
**Phosphorylated tau concentrations by mass spectrometry**				
p-tau205 (pg/ml)	0.77 (0.67 to 0.84)	<0.0001	0.78 (0.68 to 0.85)	<0.0001
p-tau208 (pg/ml)	0.65 (0.52 to 0.76)	<0.0001	0.66 (0.52 to 0.76)	<0.0001
p-tau217 (pg/ml)	0.65 (0.52 to 0.76)	<0.0001	0.66 (0.53 to 0.77)	<0.0001
p-tau199 (pg/ml)	0.56 (0.39 to 0.68)	<0.0001	0.57 (0.41 to 0.69)	<0.0001
p-tau153 (pg/ml)	0.52 (0.35 to 0.66)	<0.0001	0.53 (0.35 to 0.66)	<0.0001
p-tau231 (pg/ml)	0.50 (0.32 to 0.64)	<0.0001	0.51 (0.33 to 0.65)	<0.0001
p-tau181 (pg/ml)	0.49 (0.31 to 0.63)	<0.0001	0.51 (0.34 to 0.65)	<0.0001
p-tau202 (pg/ml)	0.46 (0.27 to 0.61)	<0.0001	0.47 (0.29 to 0.62)	<0.0001
**Non-phosphorylated tau concentrations by mass spectrometry**				
Tau195-210 (ng/ml)	0.43 (0.24 to 0.58)	<0.0001	0.46 (0.28 to 0.61)	<0.0001
Tau151-155 (ng/ml)	0.38 (0.19 to 0.55)	0.0002	0.41 (0.22 to 0.57)	<0.0001
Tau181-190 (ng/ml)	0.37 (0.18 to 0.54)	0.0003	0.40 (0.21 to 0.57)	<0.0001
Tau226-230 (ng/ml)	0.37 (0.18 to 0.54)	0.0003	0.40 (0.21 to 0.56)	<0.0001
Tau212-221 (ng/ml)	0.32 (0.13 to 0.50)	0.002	0.37 (0.17 to 0.54)	0.0004

Supplemental Table 32. Correlations between CSF biomarkers and tau PET for Braak V-VI regions for individuals in BioFINDER-2 cohort. The Spearman correlation and partial Spearman correlation after adjusting for age and sex are shown. CSF measures are shown in order of the absolute value of their correlation with tau PET, stratified by measure type. All tests were two-sided and were not adjusted for multiple comparisons.

Measure	Spearman ρ	p=	Partial Spearman ρ	p=
**CSF NeuroTool Kit measures**				
p-tau181 (pg/ml)	0.23 (0.02 to 0.42)	0.03	0.28 (0.08 to 0.47)	0.007
Aβ42 (pg/ml)	-0.23 (-0.42 to -0.02)	0.03	-0.2 (-0.4 to 0.01)	0.06
Aβ42/Aβ40	-0.20 (-0.39 to 0.01)	0.05	-0.22 (-0.41 to -0.01)	0.04
Total tau (pg/ml)	0.15 (-0.06 to 0.35)	0.15	0.22 (0.01 to 0.41)	0.04
Aβ40 (pg/ml)	-0.11 (-0.31 to 0.1)	0.32	-0.06 (-0.27 to 0.15)	0.56
**Phosphorylation occupancies by mass spectrometry**				
pT205/T205 (%)	0.66 (0.52 to 0.76)	<0.0001	0.64 (0.50 to 0.75)	<0.0001
pT217/T217 (%)	0.59 (0.43 to 0.71)	<0.0001	0.58 (0.43 to 0.71)	<0.0001
pS208/S208 (%)	0.49 (0.32 to 0.63)	<0.0001	0.48 (0.30 to 0.62)	<0.0001
pT181/T181 (%)	0.44 (0.25 to 0.59)	<0.0001	0.44 (0.25 to 0.59)	<0.0001
pT231/T231 (%)	0.42 (0.23 to 0.58)	<0.0001	0.41 (0.22 to 0.57)	<0.0001
pT153/T153 (%)	0.38 (0.19 to 0.55)	0.0002	0.37 (0.17 to 0.53)	0.0004
pT111/T111 (%)	0.21 (0.01 to 0.40)	0.04	0.22 (0.01 to 0.41)	0.04
pS199/S199 (%)	0.16 (-0.05 to 0.36)	0.13	0.13 (-0.08 to 0.33)	0.22
pS202/S202 (%)	-0.05 (-0.25 to 0.16)	0.66	-0.07 (-0.28 to 0.14)	0.50
**Phosphorylated tau concentrations by mass spectrometry**				
p-tau205 (pg/ml)	0.58 (0.43 to 0.70)	<0.0001	0.60 (0.45 to 0.72)	<0.0001
p-tau208 (pg/ml)	0.46 (0.28 to 0.61)	<0.0001	0.48 (0.30 to 0.63)	<0.0001
p-tau217 (pg/ml)	0.44 (0.26 to 0.60)	<0.0001	0.47 (0.29 to 0.62)	<0.0001
p-tau199 (pg/ml)	0.36 (0.17 to 0.53)	0.0004	0.40 (0.20 to 0.56)	0.0001
p-tau153 (pg/ml)	0.35 (0.15 to 0.52)	0.0006	0.37 (0.17 to 0.54)	0.0003
p-tau231 (pg/ml)	0.33 (0.14 to 0.51)	0.001	0.36 (0.17 to 0.53)	0.0004
p-tau202 (pg/ml)	0.29 (0.09 to 0.47)	0.005	0.34 (0.14 to 0.51)	0.001
p-tau181 (pg/ml)	0.29 (0.09 to 0.47)	0.005	0.34 (0.14 to 0.52)	0.0009
**Non-phosphorylated tau concentrations by mass spectrometry**				
Tau195-210 (ng/ml)	0.24 (0.03 to 0.42)	0.02	0.30 (0.10 to 0.48)	0.004
Tau151-155 (ng/ml)	0.21 (0.00 to 0.40)	0.05	0.27 (0.06 to 0.45)	0.01
Tau181-190 (ng/ml)	0.19 (-0.02 to 0.38)	0.07	0.26 (0.05 to 0.44)	0.01
Tau226-230 (ng/ml)	0.18 (-0.02 to 0.38)	0.08	0.25 (0.04 to 0.44)	0.02
Tau212-221 (ng/ml)	0.15 (-0.06 to 0.35)	0.15	0.23 (0.02 to 0.42)	0.03

Appendix 1. Assay methodology, quality control, and performance.

Assay methodology

CSF tau was immunopurified then digested as previously described and multiple tau phosphorylated peptides and their corresponding non-phosphorylated peptides were quantified using high resolution mass spectrometry (MS) [1]. Prior to immunopurification, 2.5 ng of fully ^{15}N-labeled 441(2N4R) tau internal standard was mixed with 450 µl of CSF sample in 0.5% NP40, 2.5 mM guanidine and protease inhibitors. Tau was immunopurified by incubating CSF with Tau1 (provided by Drs. Nicholas Kanaan and Lester Binder) and HJ8.5 (provided by Dr. David Holtzman) antibodies at room temperature for 4 hours (3 mg antibody per g of beads) [2]. Immunopurified tau was digested for 16 hours at 37°C with 400 ng of trypsin (Promega). AQUA peptides (Life Technologies, Carlsbad, CA) were added to a final sample concentration of 5 fmol per labeled phosphorylated peptide and 50 fmol per labeled unmodified peptide. The peptide mixture was purified by solid phase extraction on C18 TopTip (Glygen Corp, Columbia, MD). Eluates were dried, resuspended and transferred in MS vials. Samples were subjected to liquid chromatography and tandem high resolution mass spectrometry (LC-MS/HRMS) analysis on a nanoAcquity UPLC system (Waters, Mildford, Massachusetts) coupled to an Orbitrap Tribrid Eclipse MS (Thermo Scientific, San Jose, California) operating in PRM mode. CSF samples were run in batches of approximately 80 samples.

MS/HRMS transitions were extracted using Skyline version 22.2.2.278 (MacCoss lab, University of Washington). Signals not meeting quality criteria (peak not detected, intensity below limit of quantitation, interference on the transition used for quantitation) were not included in further analyses. LC-MS data were aggregated using Tableau version 2022.2.2 (Tableau Software, Seattle, Washington) to calculate concentrations and phosphorylation occupancies. All data extraction steps were performed by operators blinded to any clinical or biomarker information regarding the samples.

CSF tau concentrations were calculated using measured ratios between MS/HRMS transitions of endogenous non-phosphorylated peptides and ^{15}N labeled peptides from the protein internal standard on peptides 151-155, 181-190, 195-210, 212-221 and 226-230. Phosphorylation occupancies at the T111, T153, T175, T181, S202, T205, S208, T217 and T231 sites were measured using the ratio of the MS/HRMS transitions from phosphorylated peptides (103-126 for pT111; 151-155 for pT153; 171-180 for pT175; 175-190 for pT181; 195-209 for pS202, pT205 and pS208; 212-221 for pT217; 226-234 for pT231) and corresponding non-phosphorylated peptides (103-126 for T111; 151-155 for T153; 181-190 for T181 and T175, 195-209 for S202, T205 and S208; 212-221 for T217; 226-230 for T231). Each phosphorylated/non-phosphorylated peptide endogenous ratio was normalized using the ratio measured on the MS/HRMS transitions of the corresponding AQUA phosphorylated/non-phosphorylated peptide internal standards. The concentration of each p-tau isoform was calculated by multiplying the phosphorylation occupancy and corresponding non-phosphorylated tau concentration. The concentration of p-tau111 was not calculated due to the absence of corresponding 0N specific peptide in the 2N4R ^{15}N tau internal standard.

To estimate the variance of each CSF tau measure, three pools of CSF were formed that were expected to represent low, intermediate, and high values of p-tau217. Aliquots from these pools were included in each of the 26 batches run over approximately 8 weeks. Notably, pooled samples typically have higher variance than aliquots from single samples. The mean value and the coefficient of variance (mean divided by the standard deviation) for the 26 different batches are shown. The variance was typically higher for the pooled sample representing a low value of p-tau217 compared to the pooled samples representing intermediate or high values of p-tau217. For the intermediate CSF pool, pT231/T231 had higher variance (17.5%) compared to pT217/T217 (3.0%), pT181/T181 (5.8%), and pT205/T205 (9.1%).

Average values and associated coefficient of variation for three CSF pooled samples. Three CSF pooled samples designed to have low (normal), intermediate and high (abnormal) p-tau217 concentrations were included in each of 26 sample batches.

	Low pool		Intermediate pool		High pool	
	Mean	CV (%)	Mean	CV (%)	Mean	CV (%)
**Phosphorylated tau concentrations by mass spectrometry**						
p-tau217 (pg/ml)	160	9.2	690	4.7	1220	7.0
p-tau181 (pg/ml)	1710	11.1	3400	7.5	4720	7.2
p-tau202 (pg/ml)	250	11.7	350	8.2	390	10.5
p-tau205 (pg/ml)	40	12.7	100	9.5	150	10.0
p-tau208 (pg/ml)	10	32.8	20	14.7	40	15.8
p-tau199 (pg/ml)	20	35.0	60	19.0	80	16.4
p-tau231 (pg/ml)	40	45.6	240	21.4	530	28.7
p-tau153 (pg/ml)	3	53.4	13	22.9	25	20.4
p-tau175 (pg/ml)	30	32.4	40	27.6	60	24.8
**Phosphorylation occupancies by mass spectrometry**						
pT217/T217 (%)	3.8	5.3	11.3	3.0	15.2	5.3
pT181/T181 (%)	29.1	7.8	40.2	5.8	42.2	4.9
pS202/S202 (%)	4.0	10.2	3.7	8.3	3.1	8.8
pT111/T111 (%)	3.5	10.8	8.6	7.8	10.3	7.7
pT205/T205 (%)	0.65	11	1.09	9.1	1.17	8.7
pS208/S208 (%)	0.09	29.6	0.23	16.4	0.28	16.3
pS199/S199 (%)	0.71	36.7	0.59	18.4	0.59	16.1
pT231/T231 (%)	14.2	43.7	25.3	17.5	39.8	18.8
pT153/T153 (%)	0.08	49.7	0.17	19.9	0.23	20.8
pT175/T175 (%)	0.5	31.2	0.5	26.8	0.5	23.9
**Tau concentrations by mass spectrometry**						
Tau151-155 (ng/ml)	4.9	8.6	8.0	6.7	11.2	7.0
Tau181-190 (ng/ml)	5.9	7.5	8.4	5.0	11.2	6.2
Tau195-210 (ng/ml)	6.2	5.9	9.5	3.2	12.7	4.0
Tau212-221 (ng/ml)	4.4	6.8	6.1	4.3	8.01	5.6
Tau226-230 (ng/ml)	0.7	32.1	1.1	22.7	1.51	16.9

References:

1. Barthelemy NR, Toth B, Manser PT, et al. Site-Specific Cerebrospinal Fluid Tau Hyperphosphorylation in Response to Alzheimer's Disease Brain Pathology: Not All Tau Phospho-Sites are Hyperphosphorylated. *Journal of Alzheimer's disease : JAD* 2022; **85**(1): 415-29.
2. Sato C, Barthelemy NR, Mawuenyega KG, et al. Tau Kinetics in Neurons and the Human Central Nervous System. *Neuron* 2018; **98**(4): 861-4.
