# Supplementary material for: CSF tau phosphorylation occupancies at T217 and T205 represent improved biomarkers of amyloid and tau pathology in Alzheimer’s disease
Source: Nat Aging. 2023 Mar 13;3(4):391–401. doi: 10.1038/s43587-023-00380-7 (PMC10154225; doi:10.1038/s43587-023-00380-7)
Supplement: Supplementary file 2 — Reporting Summary [file 43587_2023_380_MOESM2_ESM.pdf]

nature portfolio

Corresponding author(s): Suzanne E. Schindler

Last updated by author(s): Jan 28, 2023

Reporting Summary

Nature Portfolio wishes to improve the reproducibility of the work that we publish. This form provides structure for consistency and transparency in reporting. For further information on Nature Portfolio policies, see our Editorial Policies and the Editorial Policy Checklist.

Statistics

For all statistical analyses, confirm that the following items are present in the figure legend, table legend, main text, or Methods section.

n/a	Confirmed	
☐	☒	The exact sample size (*n*) for each experimental group/condition, given as a discrete number and unit of measurement
☐	☒	A statement on whether measurements were taken from distinct samples or whether the same sample was measured repeatedly
☐	☒	The statistical test(s) used AND whether they are one- or two-sided<br>*Only common tests should be described solely by name; describe more complex techniques in the Methods section.*
☐	☒	A description of all covariates tested
☐	☒	A description of any assumptions or corrections, such as tests of normality and adjustment for multiple comparisons
☐	☒	A full description of the statistical parameters including central tendency (e.g. means) or other basic estimates (e.g. regression coefficient) AND variation (e.g. standard deviation) or associated estimates of uncertainty (e.g. confidence intervals)
☐	☒	For null hypothesis testing, the test statistic (e.g. F, t, r) with confidence intervals, effect sizes, degrees of freedom and P value noted<br>*Give P values as exact values whenever suitable.*
☒	☐	For Bayesian analysis, information on the choice of priors and Markov chain Monte Carlo settings
☒	☐	For hierarchical and complex designs, identification of the appropriate level for tests and full reporting of outcomes
☐	☒	Estimates of effect sizes (e.g. Cohen's *d*, Pearson's *r*), indicating how they were calculated

Our web collection on statistics for biologists contains articles on many of the points above.

Software and code

Policy information about availability of computer code

Data collection	MS/HRMS transitions were extracted using Skyline version 22.2.2.278 (MacCoss lab, University of Washington). Data aggregated using Tableau version 2022.2.2 (Tableau Software, Seattle, Washington) to calculate concentrations and phosphorylation occupancies.
Data analysis	Statistical analyses were implemented using SAS 9.4 (SAS Institute Inc., Cary, NC, USA). Plots were created with GraphPad Prism version 9.2.0 (GraphPad Software, La Jolla, CA, USA). For visualization of the associations between CSF measures and regional tau PET or brain volumes, partial Spearman correlations including age and sex were calculated with partial.r from the R psych toolbox version 2.1.9. The ggseg package version 1.6.5 was used to visualize correlations and results from the left hemisphere are shown. The significance of correlations was adjusted for multiple comparisons using the Benjamini-Hochberg procedure 50.

For manuscripts utilizing custom algorithms or software that are central to the research but not yet described in published literature, software must be made available to editors and reviewers. We strongly encourage code deposition in a community repository (e.g. GitHub). See the Nature Portfolio guidelines for submitting code & software for further information.

Data

Policy information about availability of data

All manuscripts must include a data availability statement. This statement should provide the following information, where applicable:

- Accession codes, unique identifiers, or web links for publicly available datasets
- A description of any restrictions on data availability
- For clinical datasets or third party data, please ensure that the statement adheres to our policy

Knight ADRC data are available to qualified investigators who have a proposal approved by an institutional committee (https://knightadrc.wustl.edu/Research/ResourceRequest.htm) that meets monthly. The study must be approved by an institutional review board to ensure ethical research practices and investigators must agree to the terms and conditions of the data use agreement, which includes not distributing the data without permission. For BioFINDER-2 data, anonymized data will be shared by request from a qualified academic investigator for the sole purpose of replicating procedures and results presented in the article and as long as data transfer is in agreement with EU legislation on the general data protection regulation and decisions by the Ethical Review Board of Sweden and Region Skåne, which should be regulated in a material transfer agreement.

Human research participants

Policy information about studies involving human research participants and Sex and Gender in Research.

Reporting on sex and gender	Sex and race were self-identified.
Population characteristics	The primary cohorts for the study were from the Knight Alzheimer Disease Research Center (Knight ADRC) at Washington University in St. Louis, MO, USA. The Knight ADRC amyloid PET cohort included 750 individuals with a median age of 71.2 years (interquartile range [IQR] 65.3 to 76.1 years); 55% were female, 90% self-identified as White, 39% carried at least one APOE ε4 allele (Extended Data Table 1), and 16% were cognitively impaired as defined by a Clinical Dementia Rating (CDR) of 0.5 or greater, which includes mild cognitive (MCI) impairment and AD dementia. The overlapping Knight ADRC tau PET cohort included 371 individuals (Extended Data Table 2). Individuals in the tau PET cohort who were cognitively impaired (CDR of 0.5 or greater) were included in Knight ADRC tau PET symptomatic AD sub-cohort (n=55), (Supplemental Table 1). The validation cohort included 90 individuals enrolled in the BioFINDER-2 cohort at Skåne University Hospital in Sweden with a median age of 72 years (IQR 67 to 76 years); 47% were female, 71% carried at least one APOE ε4 allele, 83 were diagnosed with MCI and 7 were diagnosed with AD dementia (Extended Data Table 3).
Recruitment	Participants in the Knight Alzheimer Disease Research Center (ADRC) at Washington University in St. Louis were community-dwelling volunteers enrolled in studies of memory and aging. Most participants were recruited from memory clinics or self-referred due to interest in dementia. Individuals were excluded from enrollment if they were diagnosed with a non-AD dementia at their initial assessment (e.g., Parkinson disease), had conditions that might interfere with study procedures (e.g., a pacemaker that would make the participant ineligible for MRI), or had medical issues that might significantly affect long-term participation (e.g., metastatic cancer). Participants in the Swedish BioFINDER-2 confirmatory cohort (NCT NCT03174938) included individuals diagnosed with MCI due to AD or AD dementia. Details on recruitment, exclusion and inclusion criteria have previously been described 12.<br><br>Note that the limitations section of the discussion says the following: "The study cohorts consisted of participants enrolled in research studies, and therefore the findings are most relevant to research studies and clinical trials and less generalizable to clinical populations. The cohorts were relatively young (early to mid-70s). Older cohorts, especially clinical cohorts, would be expected to have a higher prevalence of neurological comorbidities, which could complicate biomarker relationships. The cohorts included relatively few individuals with symptomatic AD and/or moderate or high levels of tau pathology. Additionally, minoritized populations were not well represented in the study cohorts, and studies have found racial differences in CSF t-tau and p-tau181 concentrations 35,36. It is unknown whether other CSF tau measures such as pT217/T217 or pT205/T205 perform similarly across racial and ethnic groups, and further studies of these measures in diverse and clinically relevant cohorts are needed."
Ethics oversight	Washington University Human Research Protection Office (HRPO) and written informed consent was obtained from each participant or their legally authorized representative when appropriate (protocol #201109100). Participants in the Swedish BioFINDER-2 confirmatory cohort (NCT NCT03174938) included individuals diagnosed with MCI due to AD or AD dementia.

Note that full information on the approval of the study protocol must also be provided in the manuscript.

Field-specific reporting

Please select the one below that is the best fit for your research. If you are not sure, read the appropriate sections before making your selection.

☒ Life sciences ☐ Behavioural & social sciences ☐ Ecological, evolutionary & environmental sciences

For a reference copy of the document with all sections, see nature.com/documents/nr-reporting-summary-flat.pdf

Life sciences study design

All studies must disclose on these points even when the disclosure is negative.

Sample size	No statistical methods were used to pre-determine sample sizes but our sample sizes are similar or larger to those used for similar studies 16,26,31.
Data exclusions	If more than one CSF sample from a participant met criteria, the most recently obtained sample was included. No data points were excluded from analyses; outliers were not removed.
Replication	Biomarker measures in a confirmatory symptomatic AD cohort<br>The CSF tau measures were further examined in participants with symptomatic AD (MCI or AD dementia) from the BioFINDER-2 cohort (n=90, Extended Data Table 5).
Randomization	Individuals were included in groups based on the procedures they underwent (i.e., CSF collection and amyloid PET and/or tau PET). All samples from an individual were run in the same batch of assays.
Blinding	All assays and data extraction steps were performed by operators blinded to any clinical or biomarker information.

Reporting for specific materials, systems and methods

We require information from authors about some types of materials, experimental systems and methods used in many studies. Here, indicate whether each material, system or method listed is relevant to your study. If you are not sure if a list item applies to your research, read the appropriate section before selecting a response.

Materials & experimental systems

n/a	Involved in the study	
☐	☒	Antibodies
☐	☐	Eukaryotic cell lines
☐	☐	Palaeontology and archaeology
☐	☐	Animals and other organisms
☐	☒	Clinical data
☐	☐	Dual use research of concern

Methods

n/a	Involved in the study	
☐	☐	ChIP-seq
☐	☐	Flow cytometry
☐	☒	MRI-based neuroimaging

Antibodies

Antibodies used	Tau was immunopurified by incubating CSF with Tau1 (provided by Drs. Nicholas Kanaan and Lester Binder) and HJ8.5 antibodies (provided by Dr. David Holtzman) at room temperature for 4 hours (3 mg antibody per g of beads) 2<br>(see Appendix 1 with more detailed assay methods and quality control metrics).
Validation	1. Barthelemy NR, Toth B, Manser PT, et al. Site-Specific Cerebrospinal Fluid Tau Hyperphosphorylation in Response to Alzheimer's Disease Brain Pathology: Not All Tau Phospho-Sites are Hyperphosphorylated. Journal of Alzheimer's disease : JAD 2022; 85(1): 415-29.<br>2. Sato C, Barthelemy NR, Mawuenyega KG, et al. Tau Kinetics in Neurons and the Human Central Nervous System. Neuron 2018; 98(4): 861-4.

Eukaryotic cell lines

Policy information about cell lines and Sex and Gender in Research

Cell line source(s)	*State the source of each cell line used and the sex of all primary cell lines and cells derived from human participants or vertebrate models.*
Authentication	*Describe the authentication procedures for each cell line used OR declare that none of the cell lines used were authenticated.*
Mycoplasma contamination	*Confirm that all cell lines tested negative for mycoplasma contamination OR describe the results of the testing for mycoplasma contamination OR declare that the cell lines were not tested for mycoplasma contamination.*
Commonly misidentified lines<br>(See ICLAC register)	*Name any commonly misidentified cell lines used in the study and provide a rationale for their use.*

Palaeontology and Archaeology

Specimen provenance	*Provide provenance information for specimens and describe permits that were obtained for the work (including the name of the issuing authority, the date of issue, and any identifying information). Permits should encompass collection and, where applicable, export.*
Specimen deposition	*Indicate where the specimens have been deposited to permit free access by other researchers.*
Dating methods	*If new dates are provided, describe how they were obtained (e.g. collection, storage, sample pretreatment and measurement), where they were obtained (i.e. lab name), the calibration program and the protocol for quality assurance OR state that no new dates are provided.*

☐ Tick this box to confirm that the raw and calibrated dates are available in the paper or in Supplementary Information.

Ethics oversight	*Identify the organization(s) that approved or provided guidance on the study protocol, OR state that no ethical approval or guidance was required and explain why not.*

Note that full information on the approval of the study protocol must also be provided in the manuscript.

Animals and other research organisms

Policy information about studies involving animals; ARRIVE guidelines recommended for reporting animal research, and Sex and Gender in Research

Laboratory animals	*For laboratory animals, report species, strain and age OR state that the study did not involve laboratory animals.*
Wild animals	*Provide details on animals observed in or captured in the field; report species and age where possible. Describe how animals were caught and transported and what happened to captive animals after the study (if killed, explain why and describe method; if released, say where and when) OR state that the study did not involve wild animals.*
Reporting on sex	*Indicate if findings apply to only one sex; describe whether sex was considered in study design, methods used for assigning sex. Provide data disaggregated for sex where this information has been collected in the source data as appropriate; provide overall numbers in this Reporting Summary. Please state if this information has not been collected. Report sex-based analyses where performed, justify reasons for lack of sex-based analysis.*
Field-collected samples	*For laboratory work with field-collected samples, describe all relevant parameters such as housing, maintenance, temperature, photoperiod and end-of-experiment protocol OR state that the study did not involve samples collected from the field.*
Ethics oversight	*Identify the organization(s) that approved or provided guidance on the study protocol, OR state that no ethical approval or guidance was required and explain why not.*

Note that full information on the approval of the study protocol must also be provided in the manuscript.

Clinical data

Policy information about clinical studies

All manuscripts should comply with the ICMJE guidelines for publication of clinical research and a completed CONSORT checklist must be included with all submissions.

Clinical trial registration	N/A--the study is NOT a clinical trial
Study protocol	N/A--the study is NOT a clinical trial
Data collection	Participants in the Knight Alzheimer Disease Research Center (ADRC) at Washington University in St. Louis were community-dwelling volunteers enrolled in studies of memory and aging. Participants in the Swedish BioFINDER-2 confirmatory cohort (NCT NCT03174938) included individuals diagnosed with MCI due to AD or AD dementia.
Outcomes	Amyloid PET positivity was previously defined as a mean cortical SUVR > 1.42 for PiB and > 1.19 for Florbetapir 45. Tau PET positivity was defined as a tau PET summary measure > 1.52 based on Gaussian mixture modeling (Supplemental Figure 1). Participants in BioFINDER-2 underwent amyloid PET using 18F-flutemetamol as previously described 12. Participants underwent tau PET using 18F-RO948 as previously described 12. For tau PET, SUVRs for the brain regions with early change (Braak I-IV) and later change (Braak V-VI) were calculated. The cut-off for positivity in the Braak I-IV region was SUVR>1.32 48.

Dual use research of concern

Policy information about dual use research of concern

Hazards

Could the accidental, deliberate or reckless misuse of agents or technologies generated in the work, or the application of information presented in the manuscript, pose a threat to:

No	Yes	
☒	☐	Public health
☒	☐	National security
☒	☐	Crops and/or livestock
☒	☐	Ecosystems
☒	☐	Any other significant area

Experiments of concern

Does the work involve any of these experiments of concern:

No	Yes	
☒	☐	Demonstrate how to render a vaccine ineffective
☒	☐	Confer resistance to therapeutically useful antibiotics or antiviral agents
☒	☐	Enhance the virulence of a pathogen or render a nonpathogen virulent
☒	☐	Increase transmissibility of a pathogen
☒	☐	Alter the host range of a pathogen
☒	☐	Enable evasion of diagnostic/detection modalities
☒	☐	Enable the weaponization of a biological agent or toxin
☒	☐	Any other potentially harmful combination of experiments and agents

ChIP-seq

Data deposition

☐ Confirm that both raw and final processed data have been deposited in a public database such as GEO.

☐ Confirm that you have deposited or provided access to graph files (e.g. BED files) for the called peaks.

Data access links<br>*May remain private before publication.*	*For "Initial submission" or "Revised version" documents, provide reviewer access links. For your "Final submission" document, provide a link to the deposited data.*
Files in database submission	*Provide a list of all files available in the database submission.*
Genome browser session<br>(e.g. UCSC)	*Provide a link to an anonymized genome browser session for "Initial submission" and "Revised version" documents only, to enable peer review. Write "no longer applicable" for "Final submission" documents.*

Methodology

Replicates	*Describe the experimental replicates, specifying number, type and replicate agreement.*
Sequencing depth	*Describe the sequencing depth for each experiment, providing the total number of reads, uniquely mapped reads, length of reads and whether they were paired- or single-end.*
Antibodies	*Describe the antibodies used for the ChIP-seq experiments; as applicable, provide supplier name, catalog number, clone name, and lot number.*
Peak calling parameters	*Specify the command line program and parameters used for read mapping and peak calling, including the ChIP, control and index files used.*
Data quality	*Describe the methods used to ensure data quality in full detail, including how many peaks are at FDR 5% and above 5-fold enrichment.*
Software	*Describe the software used to collect and analyze the ChIP-seq data. For custom code that has been deposited into a community repository, provide accession details.*

Flow Cytometry

Plots

Confirm that:

- [ ] The axis labels state the marker and fluorochrome used (e.g. CD4-FITC).
- [ ] The axis scales are clearly visible. Include numbers along axes only for bottom left plot of group (a 'group' is an analysis of identical markers).
- [ ] All plots are contour plots with outliers or pseudocolor plots.
- [ ] A numerical value for number of cells or percentage (with statistics) is provided.

Methodology

Sample preparation	*Describe the sample preparation, detailing the biological source of the cells and any tissue processing steps used.*
Instrument	*Identify the instrument used for data collection, specifying make and model number.*
Software	*Describe the software used to collect and analyze the flow cytometry data. For custom code that has been deposited into a community repository, provide accession details.*
Cell population abundance	*Describe the abundance of the relevant cell populations within post-sort fractions, providing details on the purity of the samples and how it was determined.*
Gating strategy	*Describe the gating strategy used for all relevant experiments, specifying the preliminary FSC/SSC gates of the starting cell population, indicating where boundaries between "positive" and "negative" staining cell populations are defined.*

- [ ] Tick this box to confirm that a figure exemplifying the gating strategy is provided in the Supplementary Information.

Magnetic resonance imaging

Experimental design

Design type	Structural MRI
Design specifications	N/A
Behavioral performance measures	N/A

Acquisition

Imaging type(s)	Structural MRI
Field strength	3T
Sequence & imaging parameters	Both amyloid PET and tau PET scans were performed in coordination with a 3 Tesla structural MRI scan. T1-weighted MRIs were processed using Freesurfer 5.3 to generate regions of interest used for the processing of PET data. Estimates of regional volumes were adjusted for intracranial volume using a regression approach. Data from the 30-60 minute post-injection window for PiB, the 50-70 minute window for Florbetapir, or the 80-100 minute window for Flortaucipir were converted to standardized uptake value ratios (SUVRs) using the cerebellar grey as a reference and partial volume corrected using a geometric transfer matrix 39.
Area of acquisition	Values from the following regions were averaged together to represent mean cortical SUVR for Florbetapir or PiB: bilateral orbitofrontal, medial orbitofrontal, rostral middle frontal, superior frontal, superior temporal, middle temporal, and precuneus. Amyloid PET positivity was previously defined as a mean cortical SUVR > 1.42 for PiB and > 1.19 for Florbetapir 40. Mean cortical SUVRs were converted to Centiloid units to combine data from the two tracers 40,41. Values from the bilateral entorhinal cortex, amygdala, lateral occipital cortex, and inferior temporal cortex regions were averaged together as a summary measure of tau PET42.

Diffusion MRI - [ ] Used - [x] Not used

Preprocessing

Preprocessing software	T1-weighted MRIs were processed using Freesurfer 5.3 to generate regions of interest used for the processing of PET data.
Normalization	Estimates of regional volumes were adjusted for intracranial volume using a regression approach. Data from the 30-60 minute post-injection window for PiB, the 50-70 minute window for Florbetapir, or the 80-100 minute window for Flortaucipir were converted to standardized uptake value ratios (SUVRs) using the cerebellar grey as a reference and partial volume corrected using a geometric transfer matrix 39.

Normalization template

Estimates of regional volumes were adjusted for intracranial volume using a regression approach. Data from the 30-60 minute post-injection window for PiB, the 50-70 minute window for Florbetapir, or the 80-100 minute window for Flortaucipir were converted to standardized uptake value ratios (SUVRs) using the cerebellar grey as a reference and partial volume corrected using a geometric transfer matrix 39.

Noise and artifact removal

N/A

Volume censoring

partial volume corrected using a geometric transfer matrix

Statistical modeling & inference

Model type and settings

The significance of differences by biomarker status (amyloid PET or tau PET status) were evaluated with Wilcoxon ranked sum tests for continuous variables and Chi-Square or Fisher exact tests for categorical variables. Receiver Operating Characteristic (ROC) analyses were used to evaluate the correspondence of CSF biomarker measures with amyloid PET status, tau PET status, or clinical status (cognitively unimpaired [CDR=0] or cognitively impaired [CDR>0]). Cut-offs that best distinguished amyloid PET or tau PET status were found based on the highest combined sensitivity and specificity (Youden Index). Differences between ROC areas under the curves (AUCs) were evaluated using DeLong tests 49. Spearman correlations were used to evaluate the continuous relationships of CSF biomarker measures with amyloid PET Centiloid, the tau PET summary measure, or the CDR-SB. For partial Spearman correlations with amyloid PET, tau PET, and brain volumes, analyses included covariates of age and sex; for correlations with CDR-SB, analyses included covariates of age, sex, and years of education. Comparisons between Spearman correlations were performed by bootstrapping. When multiple measures were compared, the significance was adjusted using the Benjamini-Hochberg procedure 50. Analyses were replicated in the sub-cohort of individuals with no missing data. Statistical analyses were implemented using SAS 9.4 (SAS Institute Inc., Cary, NC, USA). Plots were created with GraphPad Prism version 9.2.0 (GraphPad Software, La Jolla, CA, USA). All p values were from two-sided tests, and results were deemed statistically significant at $p < 0.05$.

For visualization of the associations between CSF measures and regional tau PET or brain volumes, partial Spearman correlations including age and sex were calculated with partial.r from the R psych toolbox. The ggseg package was used to visualize correlations and results from the left hemisphere are shown. The significance of correlations was adjusted for multiple comparisons using the Benjamini-Hochberg procedure 50.

Effect(s) tested

Differences between ROC areas under the curves (AUCs) were evaluated using DeLong tests 49. Comparisons between Spearman correlations were performed by bootstrapping.

Specify type of analysis: ☐ Whole brain ☒ ROI-based ☐ Both

Anatomical location(s)

Values from the following regions were averaged together to represent mean cortical SUVR for Florbetapir or PiB: bilateral orbitofrontal, medial orbitofrontal, rostral middle frontal, superior frontal, superior temporal, middle temporal, and precuneus. Amyloid PET positivity was previously defined as a mean cortical SUVR > 1.42 for PiB and > 1.19 for Florbetapir 40. Mean cortical SUVRs were converted to Centiloid units to combine data from the two tracers 40,41. Values from the bilateral entorhinal cortex, amygdala, lateral occipital cortex, and inferior temporal cortex regions were averaged together as a summary measure of tau PET42.

Statistic type for inference
(See Eklund et al. 2016)

N/A

Correction

When multiple measures were compared, the significance was adjusted using the Benjamini-Hochberg procedure.

Models & analysis

n/a	Involved in the study
☐	☐ Functional and/or effective connectivity
☐	☐ Graph analysis
☐	☒ Multivariate modeling or predictive analysis

Functional and/or effective connectivity

Spearman correlations were used to evaluate the continuous relationships of CSF biomarker measures with amyloid PET Centiloid, the tau PET summary measure, or the CDR-SB. For partial Spearman correlations with amyloid PET, tau PET, and brain volumes, analyses included covariates of age and sex; for correlations with CDR-SB, analyses included covariates of age, sex, and years of education. Comparisons between Spearman correlations were performed by bootstrapping.

Graph analysis

N/A

Multivariate modeling and predictive analysis

Receiver Operating Characteristic (ROC) analyses were used to evaluate the correspondence of CSF biomarker measures with amyloid PET status, tau PET status, or clinical status (cognitively unimpaired [CDR=0] or cognitively impaired [CDR>0]). Cut-offs that best distinguished amyloid PET or tau PET status were found based on the highest combined sensitivity and specificity (Youden Index).
